# Automated glycan assembly of a *S. pneumoniae* serotype 3 CPS antigen

**DOI:** 10.3762/bjoc.12.139

**Published:** 2016-07-12

**Authors:** Markus W Weishaupt, Stefan Matthies, Mattan Hurevich, Claney L Pereira, Heung Sik Hahm, Peter H Seeberger

**Affiliations:** 1 Department of Biomolecular Systems, Max Planck Institute of Colloids and Interfaces, Am Mühlenberg 1, 14476 Potsdam, Germany and Department of Chemistry and Biochemistry, Freie Universität Berlin, Arnimallee 22, 14195 Berlin, Germany

**Keywords:** automation, glycosylation, protecting groups, oligosaccharides, solid-phase synthesis, *Streptococcus pneumoniae*

## Abstract

Vaccines against *S. pneumoniae*, one of the most prevalent bacterial infections causing severe disease, rely on isolated capsular polysaccharide (CPS) that are conjugated to proteins. Such isolates contain a heterogeneous oligosaccharide mixture of different chain lengths and frame shifts. Access to defined synthetic *S. pneumoniae* CPS structures is desirable. Known syntheses of *S. pneumoniae* serotype 3 CPS rely on a time-consuming and low-yielding late-stage oxidation step, or use disaccharide building blocks which limits variability. Herein, we report the first iterative automated glycan assembly (AGA) of a conjugation-ready *S. pneumoniae* serotype 3 CPS trisaccharide. This oligosaccharide was assembled using a novel glucuronic acid building block to circumvent the need for a late-stage oxidation. The introduction of a washing step with the activator prior to each glycosylation cycle greatly increased the yields by neutralizing any residual base from deprotection steps in the synthetic cycle. This process improvement is applicable to AGA of many other oligosaccharides.

## Introduction

**Figure 1:**
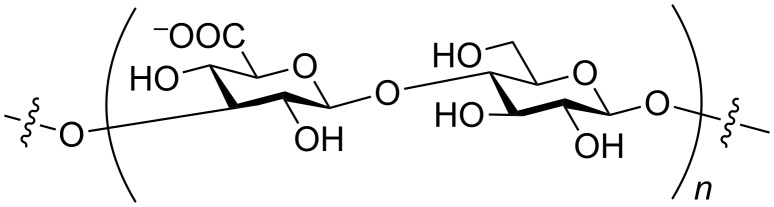
Disaccharide repeating unit of the *S. pneumoniae* serotype 3 CPS.

The Gram-positive encapsulated commensal bacterium *Streptococcus pneumoniae* [1–3] can cause serious medical conditions like pneumonia, meningitis, endocarditis and sepsis [4]. *S. pneumoniae* is the leading cause of vaccine-preventable deaths in children under five years worldwide [5]. Over 90 different serotypes of *S. pneumoniae* have been identified, each of which expresses a unique capsular polysaccharide (CPS) [6–9]. The *S. pneumoniae* serotype 3 CPS was first isolated in 1924 [10] and its exact chemical structure was finally elucidated in 1941 [11], as being composed of repeating units of β-(1,3)-linked cellobiuronic acid (Figure 1).

CPS plays a major role in *S. pneumoniae* virulence [12]. A commercial 17-valent polysaccharide vaccine was introduced in 1977, followed by a 23-valent vaccine in 1983 [13–15]. Serotype 3 of *S. pneumoniae* is one of the most prevalent serotypes causing acute otitis media [16] and is one of the thirteen serotypes included in the blockbuster pneumococcal conjugate vaccine Prevnar 13^®^ [17–18].

Vaccines against *S. pneumoniae* are usually manufactured using isolated CPS structures containing oligosaccharides of different lengths and frame shifts [19]. Synthetic oligosaccharide antigens enable structure–activity relationship (SAR) studies of bacterial antigens [20] to better understand antibody binding and help to improve existing vaccine formulations.

Two synthetic routes to prepare serotype 3 oligosaccharides have been developed and were applied to other uronic acid containing carbohydrate structures [21–22]. The first method uses only glucose building blocks to assemble oligosaccharides and introduces the C6 carboxylic acid moieties via a late-stage oxidation. Using this method, serotype 3 di-, tri- and tetrasaccharides were synthesized [23]. The other approach directly uses glucuronic acid building blocks as glycosylating agents. Due to the electron-withdrawing C6 carboxyl group, uronic acids exhibit a decreased reactivity both as glycosylating agents and as nucleophiles. Disaccharide building blocks containing glucuronic acid were used by de Jong et al. to prepare two different SP 3 trisaccharides [24]. In general, the late-stage-oxidation approach is often preferred since it circumvents the inherent reactivity issues associated with uronic acid building blocks [25–27].

Automated glycan assembly builds on monomeric building blocks that are incorporated during iterative glycosylations [28–29]. Here, a set of building blocks was identified that can be employed interchangeably in the automated syntheses of a wide variety of biologically relevant glycans. To minimize the post-automation chemical modifications and the loss of product, we assembled pneumococcal serotype 3 CPS structures utilizing glucose and glucuronic acid monosaccharide building blocks and thus avoided late-stage oxidations.

## Results and Discussion

**Figure 2:**
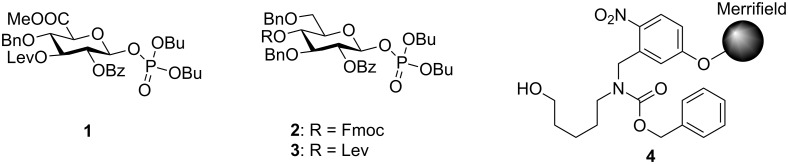
Building blocks and solid support for the automated solid-phase synthesis of *S. pneumoniae* serotype 3 CPS structures.

Mindful of this strategic framework, glucuronic acid building block **1** was designed (Figure 2). A levulinoyl (Lev) ester was chosen as temporary protecting group (TPG) since the Fmoc (fluorenylmethoxycarbonyl) group led to a loss of stereocontrol during glycosylations with this glucuronic acid (GlcA) building block (data not shown). Glucose building blocks **2** and **3** were equipped with two benzyl ethers to account for the low reactivity of glucuronic acids as glycosylating agents and carried either Fmoc or Lev groups. As solid support, we chose photolabile-linker-functionalized Merrifield resin **4** for its compatibility with the activation conditions for glycosyl phosphates, its mild cleavage conditions and the possibility to directly conjugate the product after global deprotection via the amine functional group [28]. The presence of glucuronic acids in the oligosaccharide sequence precludes the use of a base-labile linker due to the risk of elimination reactions [30].

The building blocks were synthesized in high yields using standard protecting group chemistry (see Supporting Information File 1). Solid support **4** was prepared according to an established procedure [28].

**Scheme 1:**
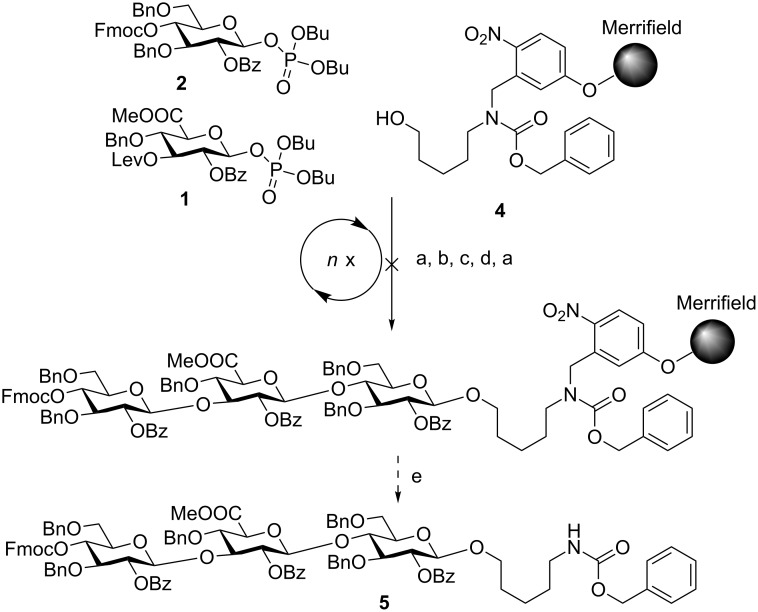
Attempted assembly of SP3 trisaccharide **5** using glycosyl phosphate building blocks **1** and **2**. Reagents and conditions: a) **2** (3 equiv), TMSOTf, CH_2_Cl_2_, −30 °C (30 min) to −15 °C (30 min), *n* = 3; b) Et_3_N in DMF (10% v/v), 25 °C (15 min), *n* = 3; c) **1** (3 equiv), TMSOTf, CH_2_Cl_2_, −30 °C (30 min) to −15 °C (30 min), *n* = 3; d) N_2_H_4_·H_2_O, pyridine/AcOH (3:2 v/v), CH_2_Cl_2_, 30 min, n = 3; e) *h*ν.

The automated glycosylation protocol employed three times three equivalents of building block to ensure complete glycosylation of the nucleophile (Scheme 1). The glycosyl phosphate building blocks **1** and **2** were activated by stoichiometric amounts of TMSOTf (trimethylsilyl trifluoromethanesulfonate) at −30 °C and reacted at this temperature for 30 min. Then the temperature was raised to −15 °C and maintained for 30 min. The temporary Fmoc protecting group was cleaved with triethylamine in DMF (*N,N*-dimethylformamide; 10% v/v). The Lev protecting group was removed using hydrazine monohydrate in pyridine/acetic acid (3:2 v/v).

**Figure 3:**
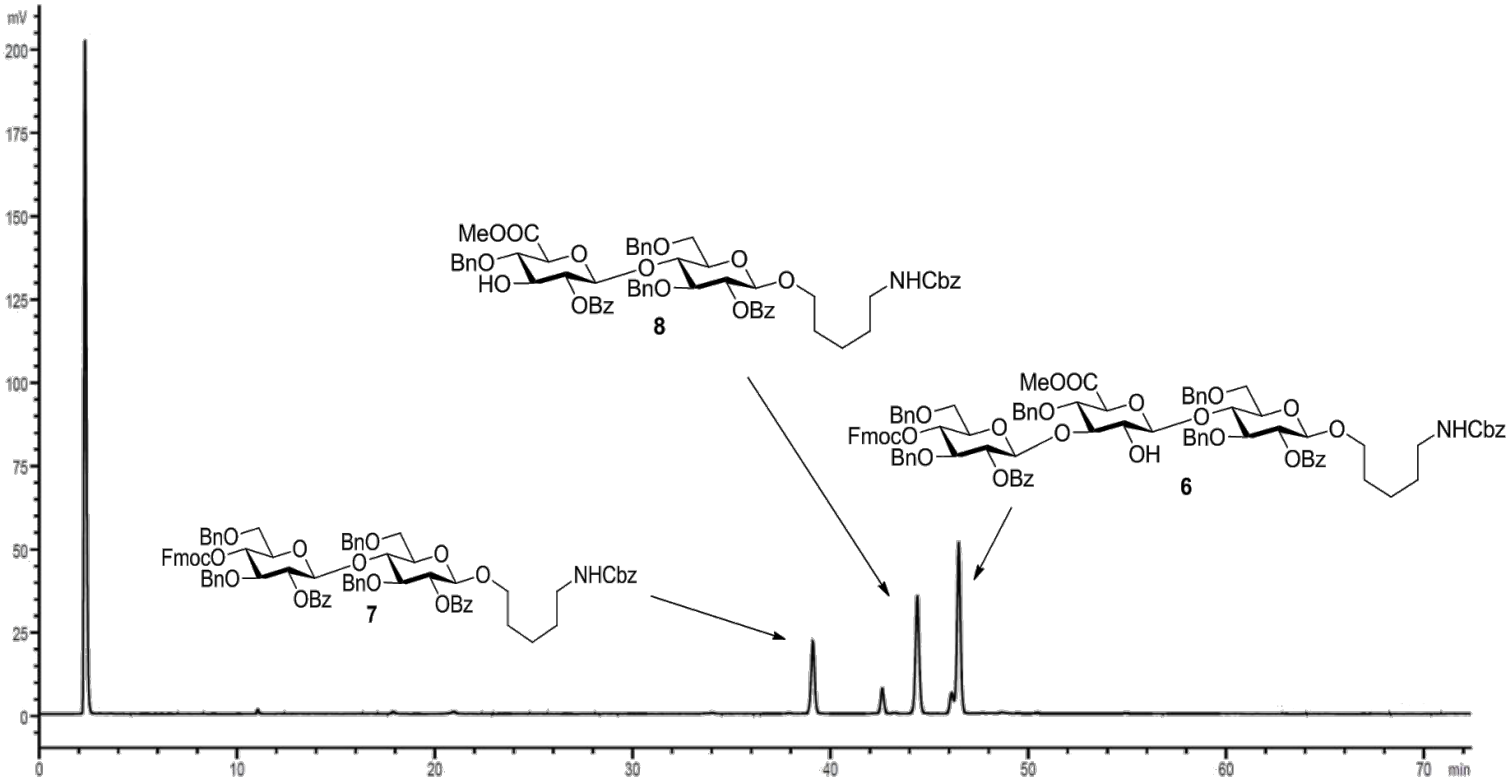
HPLC chromatogram of the crude products of the attempted AGA of SP3 trisaccharide **5**; conditions: YMC Diol 300, H/EtOAc, 0% EtOAc (5 min) to 55% EtOAc (70 min), ELSD.

The crude oligosaccharide products were cleaved from the solid support by irradiation with UV light in a flow reactor [28] and analyzed by normal-phase HPLC (Figure 3).

**Scheme 2:**
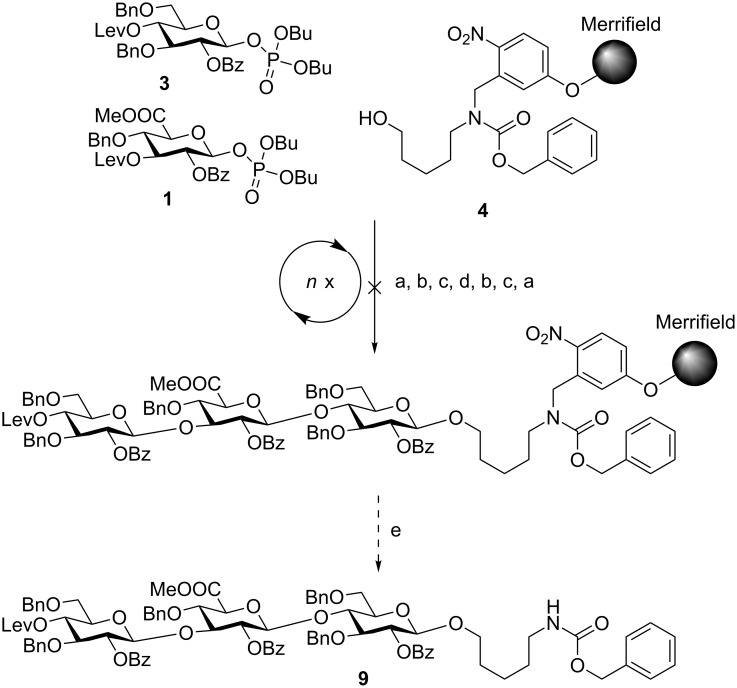
Attempted AGA of SP3 trisaccharide **9** using glycosyl phosphate building blocks **1** and **3**. Reagents and conditions: a) **3** (3 equiv), TMSOTf, CH_2_Cl_2_, −30 °C (30 min) to −15 °C (30 min), *n* = 3; b) N_2_H_4_·H_2_O, pyridine/AcOH (3:2 v/v), CH_2_Cl_2_, 30 min, *n* = 3; c) TMSOTf, CH_2_Cl_2_, −30 °C (1 min), *n* = 1; d) **1** (3 equiv), TMSOTf, CH_2_Cl_2_, −30 °C (30 min) to −15 °C (30 min), *n* = 3; e) *h*ν.

Trisaccharide **6** lacking one C2-benzoate ester protecting group was identified as the main product. The unexpected side reaction was attributed to the basicity of the Fmoc deprotection solution. In addition, two deletion sequences (**7** and **8**) were also detected. Glycosylations mediated by the strongly acidic activator TMSOTf were found to be neutral when exiting the reaction vessel. An incomplete removal of the strongly basic deprotection solutions would result in quenching of the next glycosylations. Indeed, test runs on the automated synthesis instrument illustrated regular washing steps following each deprotection failed to completely remove the deprotection solution. Therefore, an activator wash step was introduced between deprotection and glycosylation steps. In this step, the resin was washed with activator solution at −30 °C for one minute in order to neutralize any residual base. Remaining traces of water that would hydrolyze the glycosylating agent in the following glycosylation cycle are also effectively removed hereby. Furthermore, Fmoc-protected glucose building block **2** was replaced with Lev-protected **3**. The use of the buffered hydrazine solution for the cleavage of Lev TPGs was expected to prevent any undesired benzoyl ester cleavage. The trisaccharide synthesis was repeated using the same glycosylation conditions as in the previous synthesis (Scheme 2).

**Figure 4:**
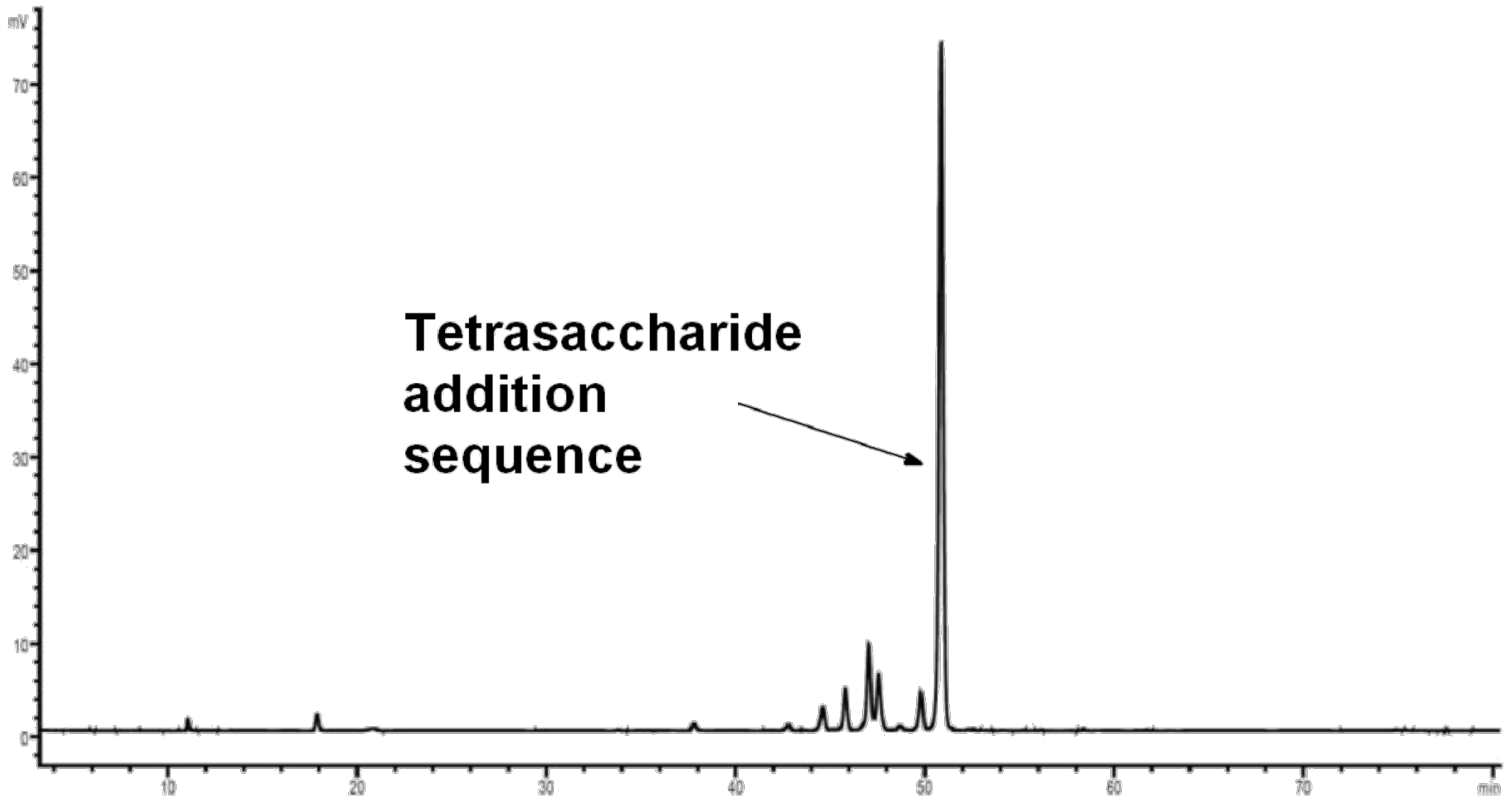
HPLC chromatogram of the crude products of the attempted AGA of SP3 trisaccharide **9**; conditions: YMC Diol 300, H/EtOAc, 0% EtOAc (5 min) to 70% EtOAc (70 min), ELSD.

After each glycosylation step, the pH of the glycosylation solutions exiting the reaction chamber was tested and found to be strongly acidic. After cleavage from the solid support, HPLC analysis of the crude product showed one major product (Figure 4). However, MALDI–TOF MS analysis indicated that this fraction corresponded to a tetrasaccharide addition sequence, resulting from benzoyl ester cleavage and a double glycosylation in the last step (see Supporting Information File 1). This result was not expected as the buffered hydrazine deprotection protocol had never favored the formation of side products in our hands. However, this finding also highlighted the efficiency of glycosylating agent **3** that can effectively glycosylate two free hydroxy groups in one step with nine equivalents of glycosylating agent **3**.

**Scheme 3:**
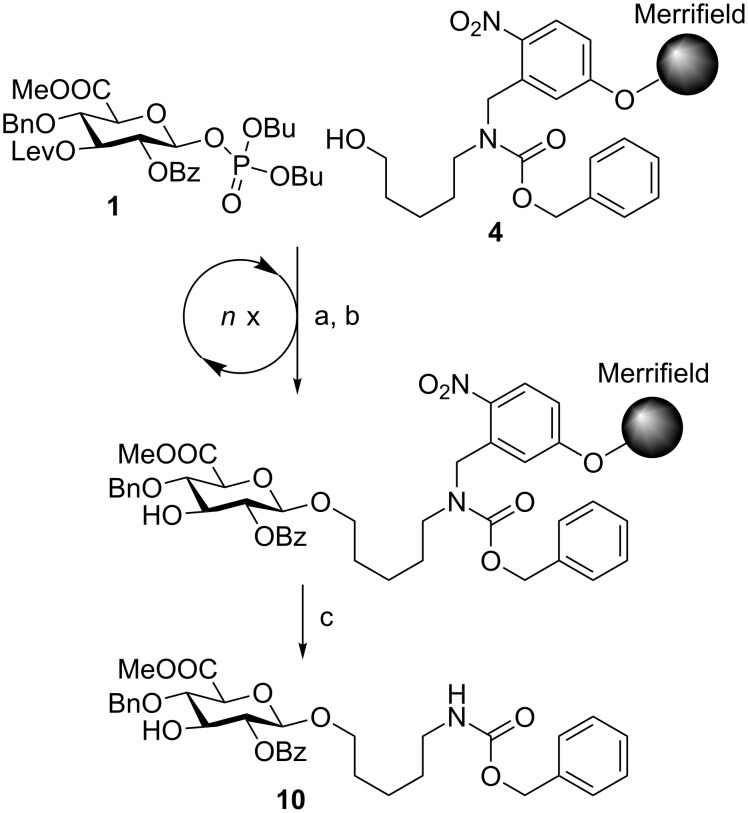
Automated synthesis of linker-bound glucuronic acid **10** using glycosyl phosphate building block **1**. Reagents and conditions: a) **1** (3 equiv), TMSOTf, CH_2_Cl_2_, −30 °C (30 min) to −15 °C (30 min), *n* = 3; b) N_2_H_4_·OAc, pyridine/AcOH (4:1 v/v), 40 °C, 10 min, *n* = 2; c) *h*ν.

Different conditions for the cleavage of the Lev protecting group on solid support had been developed previously [24,31]. Performing the reaction at elevated temperature (40 °C), it is possible to use less hydrazine acetate (7.8 equivalents). Adapting these conditions to the automated synthesizer, each Lev deprotection was followed by an activator washing step. In order to test the modified deprotection conditions, glucuronic acid **1** was reacted with the linker, and the temporary Lev protecting group was removed using the adapted deprotection protocol (Scheme 3).

The HPLC analysis showed quantitative cleavage of the Lev protecting group without loss of the benzoyl ester to afford **10** (not shown).

**Scheme 4:**
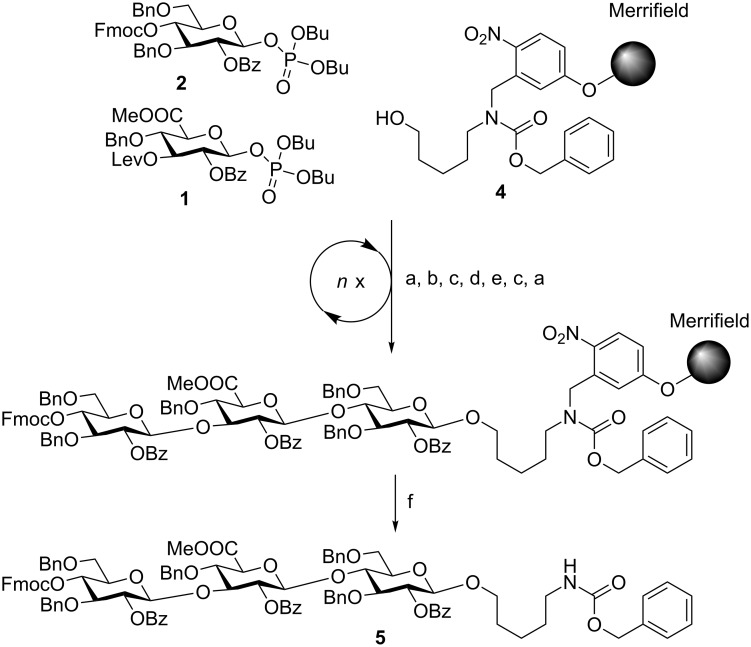
Automated synthesis of SP3 trisaccharide **5** using glycosyl phosphate building blocks **1** and **2**. Reagents and conditions: a) **2** (3 equiv), TMSOTf, CH_2_Cl_2_, −30 °C (30 min) to −15 °C (30 min), *n* = 3; b) Et_3_N in DMF (10% v/v), 25 °C (15 min), *n* = 3; c) TMSOTf, CH_2_Cl_2_, −30 °C (1 min), *n* = 1; d) **1** (3 equiv), TMSOTf, CH_2_Cl_2_, −30 °C (30 min) to −15 °C (30 min), *n* = 3; e) N_2_H_4_·OAc, pyridine/AcOH (4:1 v/v), 40 °C, 10 min, *n* = 2; f) *h*ν, 69% over 6 steps.

With this encouraging result in hand, the synthesis of *S. pneumoniae* serotype 3 CPS trisaccharide **5** was attempted utilizing the new protocol for the removal of the Lev group (Scheme 4). In order to minimize the number of Lev deprotection steps, we returned to the initial strategy using Fmoc-protected glycosyl phosphate **2** as the glucose building block. This monomer did not suffer from a loss of stereocontrol as was observed in the case of the similarly protected GlcA building block.

**Figure 5:**
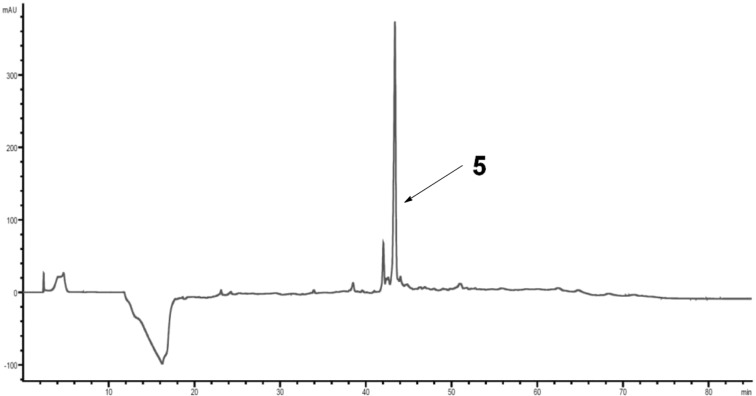
HPLC chromatogram of the crude products of the automated solid-phase SP3 trisaccharide **5** synthesis; conditions: YMC Diol 300, H/EtOAc, 0% EtOAc (5 min) to 60% EtOAc (60 min), 254 nm.

The desired trisaccharide **5** was observed as the main product from the automated synthesis by HPLC analysis (Figure 5). The Lev protecting group had been removed quantitatively while no benzoyl ester cleavage was observed. None of the byproducts could be identified by either ESIMS or NMR.

**Scheme 5:**
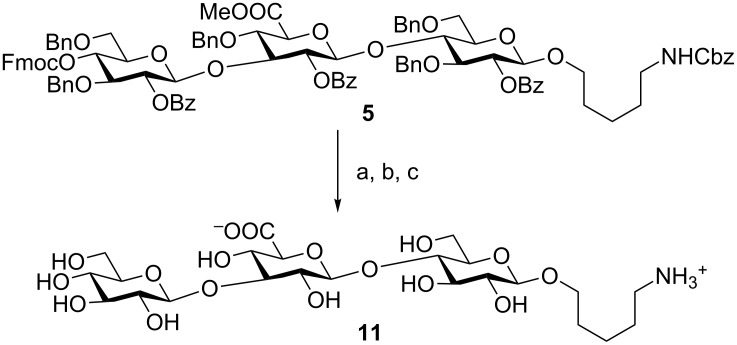
Global deprotection of SP3 trisaccharide **5**. Reagents and conditions: a) LiOH, H_2_O_2_, THF, −5 °C to rt; b) NaOH, MeOH, 0 °C to rt; c) Pd(OH)_2_/C, MeOH/H_2_O/AcOH (50:25:1 v/v/v), 71% over 3 steps.

The *S. pneumoniae* serotype 3 trisaccharide **5** was isolated in 69% yield and deprotected in three steps. First, the methyl ester was removed under mild conditions using a mixture of lithium hydroxide and hydrogen peroxide to avoid elimination reactions which are common for uronic acid methyl esters under strongly basic conditions [30,32]. In the next step, the remaining esters were removed employing sodium hydroxide in methanol. Finally, catalytic hydrogenation using Pd(OH)_2_/C in methanol/water/acetic acid (50:25:1 v/v/v) afforded the fully deprotected *S. pneumoniae* serotype 3 CPS antigen **11** in 71% yield over three steps (Scheme 5).

## Conclusion

The first automated glycan assembly of a conjugation-ready *S. pneumoniae* serotype 3 trisaccharide **11** using glucuronic acid building blocks was achieved. The need for a late-stage oxidation was circumvented by using a novel glucuronic acid building block, thereby shortening the synthetic route by two steps. Selective C6-OH deprotection/oxidation steps on oligosaccharides are usually not very efficient (53% over two steps for a trisaccharide), and are characterized by decreasing yields with increasing chain length [23]. The GlcA building block proved to be an efficient glycosylating agent, that is expected to serve well in the synthesis of other oligosaccharide antigens. Liberation of the C3-OH group of glucuronic acid **1** for chain elongation proved delicate. Standard hydrazine cleavage conditions for the Lev protecting group also removed a benzoyl ester and lead to the formation of unwanted products. Using hydrazine acetate at slightly elevated temperatures (40 °C) [24,31] cleaved the levulinoyl groups on mono- and trisaccharides while retaining all benzoyl esters. The introduction of an activator washing step prior to each glycosylation greatly increased the reproducibility of the automated syntheses and is envisioned to increase efficacy of AGA for many other biologically relevant glycans in the future.

In conclusion, we have developed an efficient method for the synthesis of *S. pneumoniae* serotype 3 CPS structures. The products of these syntheses are currently used in the development of synthetic carbohydrate conjugate vaccines.

## Supporting Information

File 1 Experimental details as well as full characterization of all new compounds.
