# Supplementary material for: Automated glycan assembly of a S. pneumoniae serotype 3 CPS antigen
Source: Beilstein J Org Chem. 2016 Jul 12;12:1440–6. doi: 10.3762/bjoc.12.139 (PMC4979738; doi:10.3762/bjoc.12.139)

Supporting Information

for

Automated glycan assembly of a *S. pneumoniae* serotype 3 CPS antigen

Markus W. Weishaupt , Stefan Matthies , Mattan Hurevich, Claney L. Pereira, Heung Sik Hahm and Peter H. Seeberger *

Address: Department of Biomolecular Systems, Max Planck Institute of Colloids and Interfaces, Am Mühlenberg 1, 14476 Potsdam, Germany and Department of Chemistry and Biochemistry, Freie Universität Berlin, Arnimallee 22, 14195 Berlin, Germany

Email: Peter H. Seeberger - peter.seeberger@mpikg.mpg.de

*Corresponding author

Experimental details as well as full characterization of all new compounds

General experimental methods

All chemicals used were reagent grade and used as supplied except where noted. Unless otherwise noted, all reactions were performed in dried glassware under an argon atmosphere. Pyridine and triethylamine (NEt_3) were distilled over CaH_2 prior to use. Dichloromethane (CH_2Cl_2), toluene, diethyl ether (Et_2O), tetrahydrofuran (THF) and *N,N*-dimethylformamide (DMF) were purchased from JT Baker or VWR International and purified by a Cycle-Tainer Solvent Delivery System unless noted otherwise. All solvents used on the automated synthesizer were extra dry grade without molecular sieves, purchased from Acros in AcroSeal® bottles. Molecular sieves 4 Å were purchased from Roth. Analytical thin-layer chromatography was performed on Macherey-Nagel silica gel SIL G-25 UV_{254} plates (0.25 mm). Compounds were visualized by UV light at 254 nm and by dipping the plates in a cerium sulfate ammonium molybdate (CAM) solution followed by heating. Liquid chromatography was performed using forced flow of the indicated solvent on Fluka silica gel 60 (230–400 mesh). Purification by reversed phase HPLC was performed using an Agilent 1200 series instrument on columns as indicated with a flow rate of 1 mL/min. ^{1}H NMR spectra were obtained on a Varian MR-400 (400 MHz) or on a Varian PremiumCOMPACT 600 (600 MHz) spectrometer and are reported in parts per million (δ) relative to the resonance of the solvent. Coupling constants (*J*) are reported in Hertz (Hz). ^{13}C NMR spectra were obtained on a Varian MR-400 (100 MHz) or on a Varian PremiumCOMPACT 600 (125 MHz) spectrometer and are reported in parts per million (δ) relative to the resonance of the solvent. MALDI-TOF spectra were measured on a Bruker autoflex speed. IR Spectra were measured neat on a Perkin-Elmer-100 FT-IR spectrometer. Specific α_D values were determined on a Schmidt+Haensch Unipol L1000. High-resolution mass spectra (ESI-TOF) were performed by the MS service at the Institute of Organic Chemistry, FU Berlin and are given in *m*/*z*.

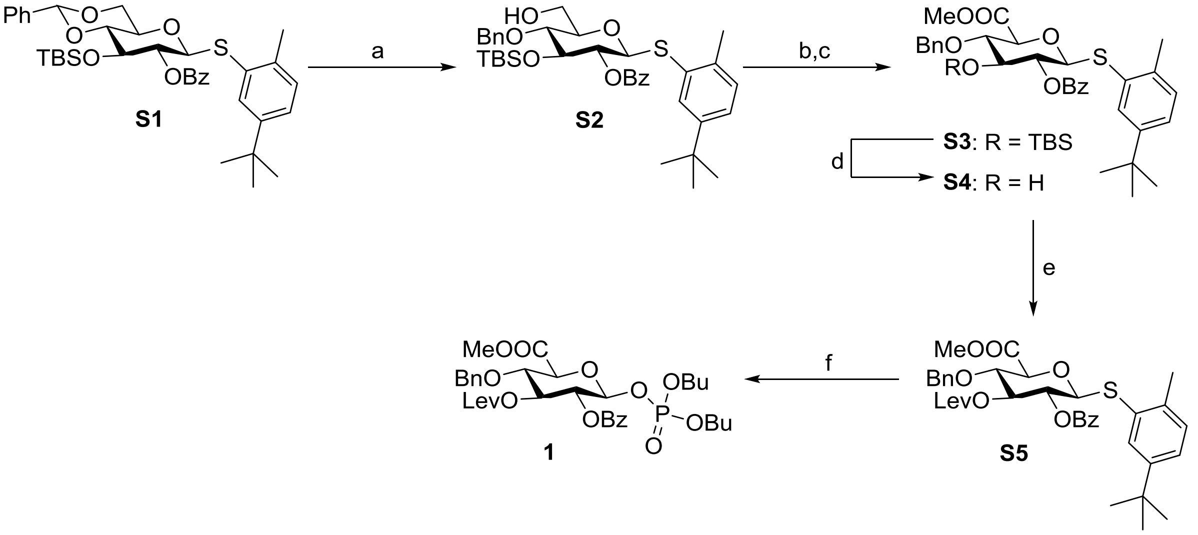

Scheme S1: Synthesis of building block **1**. Reagents and conditions: a) BH_3·THF, TMSOTf, CH_2Cl_2, 92%; b) PhI$(OAc)_2$, TEMPO, CH_2Cl_2, H_2O; c) trimethylsilyl diazomethane, DMF, 94% **S3** over two steps; d) TBAF·3 H_2O, AcOH, DMF, 91%; e) LevOH, DIC, DMAP, CH_2Cl_2, 89%; f) dibutyl phosphate, 4 Å MS, NIS, TfOH, CH_2Cl_2, 88%.

Compound **S1** was prepared according to published procedures [1].

(2-Methyl-5-*tert*-butylphenyl) 4-*O*-benzyl-2-*O*-benzoyl-3-*O*-*tert*-butyldimethylsilyl-1-thio-β-D-glucopyranoside (S2).

Compound **S1** (50 mg, 77 µmol) was coevaporated with toluene and dissolved in CH_2Cl_2 (770 µL) under argon atmosphere. BH_3·THF (460 µL, 0.462 mmol) was added and the solution was stirred at 0 °C for 15 min. Then TMSOTf (7 µL, 39 µmol) was added and the reaction mixture was warmed to room temperature and stirred overnight. After completion, the reaction was cooled to 0 °C, quenched with MeOH (31 µL) and neutralized with Et_3N (5 drops). The solvents were evaporated in vacuo, the residue dissolved in CH_2Cl_2, extracted with H_2O, dried over $MgSO_4$ and filtered. The solvent was evaporated and the crude product was purified by flash column chromatography (silica gel, cyclohexane/ethyl acetate) to afford **S2** (46 mg, 71 µmol). R_f = 0.23 (cyclohexane/ethyl acetate, 3:1); $[\alpha]_D^{20}$ = +42.63 (c = 1.0, CH_2Cl_2); IR (thin film, cm^{-1}): 3464, 2957, 2929, 2858, 1732, 1261, 1070, 1028, 838, 778, 708; ^{1}H NMR (400 MHz, $CDCl_3$) δ 8.10 – 8.05 (m, 2H), 7.61 – 7.55 (m, 1H), 7.51 (d, *J* = 2.0 Hz, 1H), 7.45 (t, *J* = 7.7 Hz, 2H), 7.38 – 7.27 (m,

5H), 7.19 (dd, *J* = 8.0, 2.0 Hz, 1H), 7.07 (d, *J* = 8.0 Hz, 1H), 5.30 (dd, *J* = 10.0, 9.0 Hz, 1H), 4.89 (d, *J* = 11.5 Hz, 1H), 4.77 (d, *J* = 10.2 Hz, 1H), 4.67 (d, *J* = 11.5 Hz, 1H), 3.99 (t, *J* = 8.8 Hz, 1H), 3.88 (d, *J* = 12.0 Hz, 1H), 3.75 – 3.60 (m, 2H), 3.47 (ddd, *J* = 9.7, 4.5, 2.5 Hz, 1H), 2.16 (s, 3H), 1.28 (s, 9H), 0.80 (s, 9H), 0.03 (s, 3H), -0.14 (s, 3H); ^{13}C NMR (101 MHz, $CDCl_3$) δ 165.6, 149.8, 138.0, 136.5, 133.3, 133.2, 130.3, 130.1, 130.0, 129.0, 128.6, 128.5, 127.9, 127.8, 125.1, 87.7, 79.7, 78.5, 76.8, 75.3, 73.4, 62.2, 31.4, 25.8, 20.3, -3.9, -4.1. HRMS calcd for $C_{37}H_{50}O_6SSiNa$ $[M + Na]^+$, 673.2990; found, 673.3011.

Methyl [(2-methyl-5-*tert*-butylphenyl) 2-*O*-benzoyl-4-*O*-benzyl-3-*O*-*tert*-butyldimethylsilyl-1-thio-β-D-glucopyranosyl]uronate (S3).

Starting material **S2** (3.56 g, 5.5 mmol) was dissolved in CH_2Cl_2/H_2O (27 mL, 2:1 v/v) and cooled to 0 °C. TEMPO (171 mg, 1.1 mmol) was added, followed by the addition of BAIB (3.7 g, 11.5 mmol). The mixture was stirred for 30 min at 0 °C and for 4.5 h at room temperature (TLC: hexanes/ethyl acetate, 7:1). The reaction mixture was quenched by the addition of saturated aqueous $Na_2S_2O_3$, diluted with CH_2Cl_2 and extracted with saturated aqueous $Na_2S_2O_3$ (2×). The organic phase was dried over $MgSO_4$ and the solvents were evaporated in vacuo. The colorless solid was dried in high vacuum and used for the next reaction step without further purification. (2-Methyl-5-*tert*-butylphenyl) 2-*O*-benzoyl-4-*O*-benzyl-3-*O*-*tert*-butyldimethylsilyl-1-thio-β-D-glucopyranosyluronate (3.64 g, 5.5 mmol) was dissolved in anhydrous DMF (61 mL) under argon, and a 2 M solution of trimethylsilyldiazomethane in hexanes (5.5 mL, 10.9 mmol) was added dropwise. Once the evolution of gas ceased, the reaction mixture was stirred overnight at room temperature. The reaction was quenched by the addition of acetic acid, concentrated in vacuo and coevaporated with toluene (3 x). The crude product was purified using flash column chromatography (silica gel, hexanes/ethyl acetate) to afford glucuronic acid methyl ester **S3** (3.5 g, 94%). R_f = 0.32 (hexanes/ethyl acetate, 9.5:0.5); $[\alpha]_D^{20}$ = +20.79 (c = 1.0, $CHCl_3$); IR (thin film, cm^{-1}): 2954, 2929, 2858, 1751, 1733, 1233, 1154, 1027, 1091, 1070, 839, 779, 709; ^{1}H NMR (400 MHz, $CDCl_3$) δ 8.06 (dd, *J* = 8.3, 1.2 Hz, 2H), 7.60 – 7.55 (m, 2H), 7.45 (t, *J* = 7.7 Hz, 2H), 7.36 – 7.24 (m, 5H), 7.19 (dd, *J* = 8.0, 2.1 Hz, 1H), 7.05 (d, *J* = 8.0 Hz, 1H), 5.32 (dd, *J* = 10.1, 8.4 Hz, 1H), 4.80 (d, *J* = 11.1 Hz, 1H), 4.75 (d, *J* = 10.1 Hz, 1H), 4.62 (d, *J* = 11.1 Hz, 1H), 4.02 – 3.89 (m, 3H), 3.69 (s, 3H), 2.13 (s, 3H), 1.28 (s, 9H), 0.79 (s, 9H), -0.00 (s, 3H), -0.17 (s, 3H); ^{13}C NMR (101 MHz, $CDCl_3$) δ 168.5, 165.4, 149.8, 137.9, 136.7, 133.3, 133.0, 130.2, 130.1, 130.1, 129.9, 129.7, 128.6,

128.5, 128.5, 127.8, 127.8, 125.2, 88.4, 80.2, 78.4, 76.4, 75.3, 72.9, 52.6, 31.3, 25.8, 20.3, -3.9, -4.2; HRMS calcd for $C_{38}H_{50}O_7SSiNa$ $[M + Na]^+$, 701.2939; found, 701.2959.

Methyl [(2-methyl-5-*tert*-butylphenyl) 2-*O*-benzoyl-4-*O*-benzyl-1-thio-β-D-glucopyranosyl]uronate (S4).

Glucuronic acid methyl ester **S3** (190 mg, 0.3 mmol) was dissolved in DMF (1.5 mL) and cooled to 0 °C. A solution TBAF·3 H_2O (530 mg, 1.7 mmol) and glacial acetic acid (130 µL, 2.2 mmol) in DMF (1.5 mL) was added dropwise, and the reaction mixture was warmed to 35 °C and stirred overnight. Upon completion, the reaction was cooled to room temperature, diluted with Et_2O and extracted with 1 M HCl (aq) and saturated aqueous $NaHCO_3$. The organic layer was dried over $MgSO_4$ and filtered. The solvent was evaporated in vacuo and the crude material was purified by flash column chromatography (silica gel, hexanes/ethyl acetate) to afford **S4** (155 mg, 0.29 mmol). R_f = 0.33 (hexanes/ethyl acetate, 4:1); $[\alpha]_D^{20}$ = -10.51 (c = 2.1, CH_2Cl_2); IR (thin film, cm^{-1}): 3494, 2957, 1731, 1263, 1092, 710; ^1H NMR (400 MHz, $CDCl_3$) δ 8.11 – 8.05 (m, 1H), 7.60 (dd, *J* = 10.8, 4.6 Hz, 2H), 7.46 (t, *J* = 7.7 Hz, 2H), 7.37 – 7.20 (m, 6H), 7.09 (d, *J* = 8.0 Hz, 1H), 5.15 – 5.07 (m, 1H), 4.83 – 4.71 (m, 3H), 4.01 – 3.86 (m, 3H), 3.78 (s, 3H), 2.21 (s, 3H), 1.28 (s, 9H); ^{13}C NMR (101 MHz, $CDCl_3$) δ 168.3, 166.4, 149.8, 137.9, 137.2, 133.7, 131.8, 130.3, 130.2, 130.1, 130.0, 129.5, 128.7, 128.6, 128.4, 128.3, 128.2, 128.1, 128.0, 125.5, 87.2, 79.3, 78.0, 77.1, 75.2, 73.1, 52.7, 31.3, 20.4; HRMS calcd for $C_{32}H_{36}O_7SNa$ $[M + Na]^+$, 587.2074; found, 587.2060.

Methyl [(2-methyl-5-*tert*-butylphenyl) 2-*O*-benzoyl-4-*O*-benzyl-3-*O*-levulinoyl-1-thio-β-D-glucopyranosyl]uronate (S5).

Thioglycoside **S4** (145 mg, 0.26 mmol) was dissolved in CH_2Cl_2 (2.6 mL) under argon. Levulinic acid (100 µL, 0.97 mmol) was added dropwise with stirring, followed by the dropwise addition of DIC (100 µL, 0.64 mmol). DMAP (3 mg, 26 µmol) was added to the reaction mixture, and the reaction was stirred overnight at room temperature. Upon completion (TLC: hexane/ethyl acetate, 3:1), the reaction mixture was diluted with CH_2Cl_2 and washed with 1 M HCl (aq) and saturated aqueous $NaHCO_3$. The organic layer was dried over $MgSO_4$, filtered and the solvents were evaporated in vacuo. The crude material was purified by flash column chromatography (silica gel, hexane/ethyl acetate) to afford compound **S5** (152 mg, 0.23 mmol) as a white solid. R_f = 0.32 (hexane/ethyl acetate, 3:1); $[\alpha]_D^{20}$ = +22.77 (c = 1.0, $CHCl_3$); IR (thin film, cm^{-1}): 2957, 1747, 1724, 1264, 1092, 1070, 711; ^1H NMR (400 MHz,

$CDCl_3$) δ 8.05 – 7.99 (m, 2H), 7.62 – 7.53 (m, 2H), 7.48 – 7.41 (m, 2H), 7.37 – 7.19 (m, 6H), 7.07 (d, *J* = 8.0 Hz, 1H), 5.45 (ddd, *J* = 9.2, 7.4, 1.6 Hz, 1H), 5.29 – 5.23 (m, 1H), 4.83 (d, *J* = 10.1 Hz, 1H), 4.65 (d, *J* = 1.6 Hz, 2H), 4.10 – 4.01 (m, 2H), 3.76 (s, 3H), 2.58 – 2.28 (m, 3H), 2.18 (s, 3H), 2.01 (s, 3H), 1.28 (s, 9H); ^{13}C NMR (101 MHz, $CDCl_3$) δ 205.8, 171.9, 168.0, 165.4, 149.9, 137.6, 137.1, 133.5, 131.9, 130.2, 130.1, 129.4, 128.6, 128.5, 128.2, 128.1, 125.5, 87.8, 78.2, 77.1, 75.7, 75.0, 70.7, 52.8, 37.9, 34.6, 31.3, 29.7, 28.1, 20.3; HRMS calcd for $C_{37}H_{42}O_9SNa$ $[M + Na]^+$, 685.2442; found, 685.2431.

Dibutyl [methyl(2-*O*-benzoyl-4-*O*-benzyl-3-*O*-levulinoyl-β-D-glucopyranosyl)uronate]phosphate (1).

Thioglycoside **S5** (85 mg, 0.128 mmol), freshly activated 4 Å molecular sieves and dibutyl phosphate (76 µL, 0.385 mmol) were suspended in CH_2Cl_2 (1.4 mL) under argon and stirred for 30 min at room temperature. The suspension was cooled to 0 °C, NIS (38 mg, 0.167 mmol) was added, and the mixture was stirred for an additional 30 min at 0 °C. TfOH (1 µL, 13 µmol) was added with vigorous stirring, and the reaction was stirred at 0 °C for 30 min. Upon complete conversion of the starting material (TLC: toluene/acetone, 9:1), the reaction was quenched by the addition of triethylamine and washed with sat. $NaHCO_3$ (aq) and sat. $Na_2S_2O_3$ (aq). The organic layer was dried over $MgSO_4$ and filtered, and the volatiles were removed in vacuo. The crude material was purified by flash column chromatography (silica gel, toluene/acetone) to afford glycosyl phosphate **1** (78 mg, 0.113 mmol) as a colorless solid. R_f = 0.21 (toluene/acetone, 9:1); $[\alpha]_D^{20}$ = +7.07 (c = 1.0, CH_2Cl_2); IR (thin film, cm^{-1}): 2961, 1748, 1721, 1265, 1026, 713; ^{1}H NMR (600 MHz, $CDCl_3$) δ 8.00 (dd, *J* = 8.4, 1.2 Hz, 2H), 7.59 – 7.55 (m, 1H), 7.45 – 7.41 (m, 2H), 7.35 – 7.30 (m, 2H), 7.30 – 7.23 (m, 3H), 5.45 (dt, *J* = 16.1, 8.5 Hz, 2H), 5.31 (dd, *J* = 9.6, 7.9 Hz, 1H), 4.63 (q, *J* = 11.3 Hz, 2H), 4.17 (d, *J* = 9.6 Hz, 1H), 4.07 – 3.97 (m, 3H), 3.79 – 3.73 (m, 4H), 3.72 – 3.66 (m, 1H), 2.58 – 2.32 (m, 4H), 2.01 (s, 3H), 1.63 – 1.58 (m, 2H), 1.39 – 1.24 (m, 4H), 1.04 (dd, *J* = 15.1, 7.5 Hz, 2H), 0.89 (t, *J* = 7.4 Hz, 3H), 0.69 (t, *J* = 7.4 Hz, 3H); ^{13}C NMR (151 MHz, $CDCl_3$) δ 205.8, 171.7, 167.9, 165.2, 137.4, 133.7, 130.1, 129.1, 128.7, 128.6, 128.2, 128.2, 96.5, 96.4, 74.9, 74.8, 73.9, 73.9, 71.9, 71.9, 68.3, 68.3, 68.1, 68.1, 52.8, 37.8, 32.2, 32.1, 31.9, 31.9, 29.7, 28.0, 18.7, 18.4, 13.7, 13.5; ^{31}P NMR (243 MHz, $CDCl_3$) δ -3.03; HRMS calcd for $C_{34}H_{45}O_{13}PNa$ $[M + Na]^+$, 715.2490; found, 715.2497.

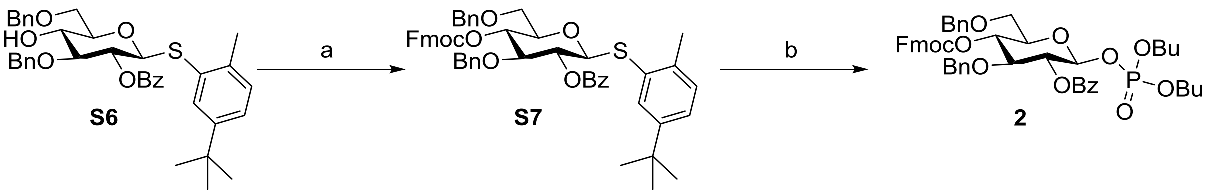

Scheme S2: Synthesis of building block **2**. Reagents and conditions: a) FmocCl, pyridine, CH_2Cl_2, 95%; b) dibutyl phosphate, 4 Å MS, NIS, TfOH, CH_2Cl_2, 72%.

Compound **S6** was prepared according to published procedures [2].

(2-Methyl-5-*tert*-butylphenyl) 2-*O*-Benzoyl-3,6-di-*O*-benzyl-4-*O*-fluorenylmethoxycarbonyl-1-thio-β-D-glucopyranoside (S7).

Thioglycoside **S6** (250 mg, 0.4 mmol) and FmocCl (155 mg, 0.6 mmol) were dissolved in CH_2Cl_2 (8 mL) under argon, and pyridine (100 µl, 1.2 mmol) was added dropwise. The reaction was stirred at room temperature overnight (TLC: hexanes/ethyl acetate, 9:1). The mixture was diluted with CH_2Cl_2 and extracted with 1 M HCl (aq) and saturated aqeous $NaHCO_3$. The organic layer was dried over $MgSO_4$ and filtered. The solvent was evaporated in vacuo and the crude material was purified by flash column chromatography (silica gel, hexanes/ethyl acetate) to afford compound **S7** (322 mg, 0.38 mmol). R_f = 0.30 (cyclohexane/ethyl acetate, 9:1); $[\alpha]_D^{20}$ = +41.91 (c = 1.0, $CHCl_3$); IR (thin film, cm^{-1}): 2960, 1753, 1733, 1451, 1251, 1070; ^{1}H NMR (400 MHz, $CDCl_3$) δ 8.03 (dd, *J* = 8.4, 1.3 Hz, 2H), 7.75 (dd, *J* = 7.5, 3.7 Hz, 2H), 7.63 – 7.50 (m, 4H), 7.49 – 7.42 (m, 2H), 7.42 – 7.35 (m, 2H), 7.33 – 7.16 (m, 8H), 7.09 – 7.01 (m, 6H), 5.38 (dd, *J* = 10.1, 9.1 Hz, 1H), 5.08 – 4.97 (m, 1H), 4.75 (d, *J* = 10.1 Hz, 1H), 4.62 – 4.48 (m, 4H), 4.32 (d, *J* = 7.2 Hz, 2H), 4.11 (t, *J* = 7.2 Hz, 1H), 3.92 (t, *J* = 9.1 Hz, 1H), 3.79 – 3.72 (m, 1H), 3.67 (d, *J* = 4.7 Hz, 2H), 2.20 (s, 3H), 1.23 (s, 9H); ^{13}C NMR (101 MHz, $CDCl_3$) δ 165.2, 154.3, 149.8, 143.4, 143.2, 141.4, 141.4, 137.9, 137.4, 137.0, 133.4, 132.7, 130.0, 130.0, 129.8, 128.6, 128.5, 128.3, 128.0, 127.9, 127.8, 127.3, 125.2, 125.2, 125.1, 120.2, 87.7, 81.2, 77.4, 75.5, 74.4, 73.7, 72.1, 70.2, 69.7, 46.8, 34.5, 31.4, 20.4; HRMS calcd for $C_{53}H_{52}O_8SNa$ [M + Na]$^+$, 871.3275; found, 871.3289.

Dibutyl 2-*O*-Benzoyl-3,6-di-*O*-benzyl-4-*O*-fluorenylmethoxycarbonyl-β-D-glucopyranosyl phosphate (2).

Thioglycoside **S7** (100 mg, 0.118 mmol), 4 Å molecular sieves and dibutyl phosphate (70 µL, 0.353 mmol) were suspended in CH_2Cl_2 (1.3 mL) under argon and stirred for 30 min at rt. The suspension was cooled to 0 °C, NIS (34 mg, 0.153 mmol) was added, and the mixture was stirred for an additional 30 min at 0 °C. TfOH (1 µL, 12 µmol) was added with vigorous stirring, and the reaction was stirred at 0 °C for 30 min. Upon complete conversion of the starting material (TLC: hexane/ethyl acetate, 2:1), the reaction was quenched by the addition of pyridine and washed with saturated aqueous $NaHCO_3$ and saturated aqueous $Na_2S_2O_3$. The organic layer was dried over $MgSO_4$ and filtered, and the volatiles were removed in vacuo. The crude material was purified by flash column chromatography (silica gel, toluene/acetone) to afford glycosyl phosphate **2** (75 mg, 0.085 mmol) as a colorless solid. R_f = 0.28 (hexane/ethyl acetate, 2:1); $[\alpha]_D^{20}$ = +31.0 (c = 1.0, $CHCl_3$); IR (thin film, cm^{-1}): 2961, 2874, 1753, 1733, 1249, 1028, 738; ^1H NMR (400 MHz, $CDCl_3$) δ 8.04 – 7.99 (m, 2H), 7.79 – 7.72 (m, 2H), 7.61 – 7.52 (m, 3H), 7.48 – 7.36 (m, 4H), 7.34 – 7.20 (m, 7H), 7.13 – 7.02 (m, 5H), 5.44 – 5.37 (m, 2H), 5.14 – 5.06 (m, 1H), 4.63 – 4.49 (m, 4H), 4.41 – 4.27 (m, 2H), 4.13 (t, *J* = 7.1 Hz, 1H), 4.08 – 3.98 (m, 2H), 3.94 – 3.82 (m, 2H), 3.78 – 3.61 (m, 4H), 1.64 – 1.51 (m, 2H), 1.38 – 1.22 (m, 4H), 1.08 – 0.92 (m, 2H), 0.87 (t, *J* = 7.4 Hz, 3H), 0.68 (t, *J* = 7.4 Hz, 3H); ^{13}C NMR (101 MHz, $CDCl_3$) δ 165.0, 154.2, 143.4, 143.2, 141.4, 141.4, 137.8, 137.2, 133.6, 130.0, 129.4, 128.6, 128.5, 128.3, 128.1, 128.1, 127.8, 127.8, 127.7, 127.3, 125.2, 125.1, 120.2, 96.7, 96.6, 79.2, 75.1, 74.4, 73.8, 73.7, 73.0, 72.9, 70.2, 69.2, 68.2, 68.1, 68.1, 68.0, 46.8, 32.2, 32.1, 31.9, 31.8, 18.7, 18.3, 13.7, 13.5; ^{31}P NMR (162 MHz, $CDCl_3$) δ -2.81; HRMS calcd for $C_{50}H_{55}O_{12}PNa$ $[M + Na]^+$, 901.3323; found, 901.3379.

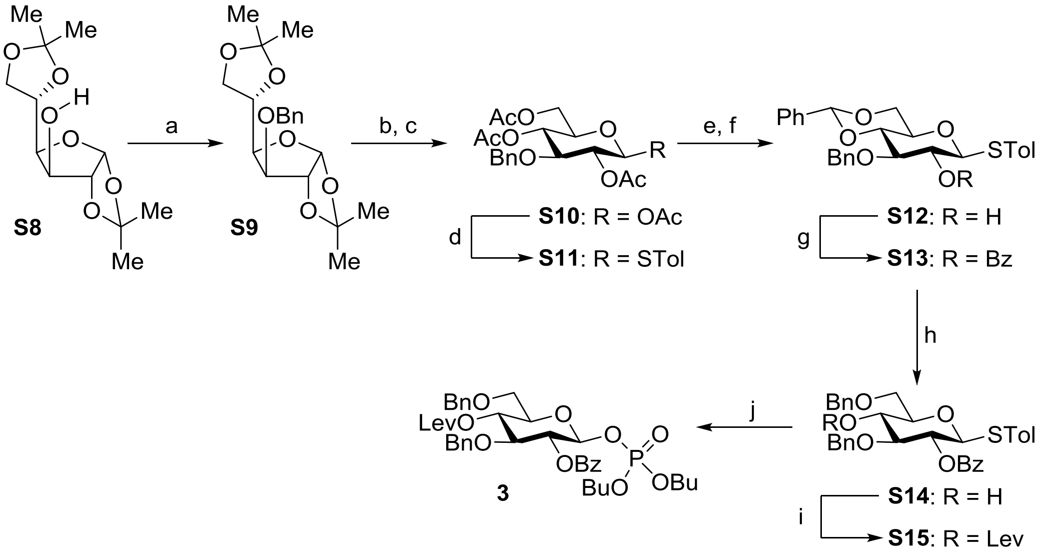

Scheme S3: Synthesis of building block **3**. Reagents and conditions: a) BnBr, NaH, THF/DMF (9:1 v/v), quant.; b) Amberlite IR-120 (H^+ form); c) Ac_2O/pyridine, 53% over two steps; d) *p*-toluenethiol, $BF_3 \cdot OEt_2$, CH_2Cl_2, 76%; e) NaOMe, MeOH; f) benzaldehyde dimethyl acetal, *p*-TsOH·H_2O, CH_3CN, 79% over two steps; g) benzoic acid, DIC, DMAP, CH_2Cl_2, 66%; h) Et_3SiH, TFA, TFAA, CH_2Cl_2, 87%; i) LevOH, DIC, DMAP, CH_2Cl_2, 90%; j) dibutyl phosphate, 4 Å MS, NIS, TfOH, CH_2Cl_2, 79%.

3-*O*-Benzyl-1,2;5,6-di-*O*-isopropylidene-α-D-glucofuranoside (S9).

1,2;5,6-di-*O*-isopropylidene-α-D-glucofuranose (41.4 g, 159 mmol) **S8** was dissolved in THF/DMF (9:1 v/v, 883 mL) under argon. Sodium hydride (60% in mineral oil, 8.9 g, 223 mmol) was added, and the suspension was cooled to 0 °C. Benzyl bromide (24.6 mL, 207 mmol) was added dropwise. The solution was warmed to room temperature and stirred for 18 h. Upon completion, the reaction was cooled to 0 °C and quenched by the dropwise addition of MeOH, diluted with ether and washed with brine. The organic layer was dried over $MgSO_4$ and filtered. The solvent was evaporated in vacuo, and the crude material was used in the next reaction step without further purification. R_f = 0.71 (cyclohexane/ethyl acetate, 1:1).

1,2,4,6-Tetra-*O*-acetyl-3-*O*-benzyl-β-D-glucopyranoside (S10).

To compound **S9**, H_2O (500 mL) and of Amberlite IR-120 (H^+ form, 58 g) were added. The mixture was heated to 70 °C and stirred for 15 h. After cooling the reaction mixture to room temperature, the resin was filtered off and washed with water. The aqueous layer was washed with a mixture of ether and EtOAc (1:1 v/v). The aqueous layer was then evaporated to obtain a white solid which was coevaporated with toluene and dried in vacuo. The crude material was taken up in pyridine/acetic anhydride (350 mL, 4:3 v/v) and stirred at room temperature. After completion, the solvents were removed in vacuo and the resulting oil was coevaporated with toluene two times. The brown oil was recrystallized from ethanol, filtered, and repeatedly washed with cyclohexane. The resulting solid was dried in vacuo to afford beta anomer **S10** (36.9 g, 84.3 mmol). R_f = 0.59 (cyclohexane/ethyl acetate, 1:1); ^1H NMR (400 MHz, $CDCl_3$) δ 7.44 – 7.13 (m, 5H), 5.65 (d, *J* = 8.2 Hz, 1H), 5.23 – 5.08 (m, 2H), 4.61 (s, 2H), 4.22 (dd, *J* = 12.4, 4.9 Hz, 1H), 4.09 (dd, *J* = 12.4, 2.3 Hz, 1H), 3.73 (m, 2H), 2.10 (s, 3H), 2.08 (s, 3H), 1.98 (s, 6H).

All spectroscopic data are in good accordance with the literature [3-6].

4-Methylphenyl 2,4,6-tri-*O*-acetyl-3-*O*-benzyl-1-thio-β-D-glucopyranoside (S11).

Compound **S10** (20.4 g, 46.5 mmol) was dissolved in CH_2Cl_2 (172 mL) and the solution was cooled to 0 °C. *p*-Toluenethiol (6.94 g, 55.8 mmol) and $BF_3{\cdot}OEt_2$ (17.69 ml, 140 mmol) were added. The mixture was kept at 0 °C for 20 h. The mixture was diluted with CH_2Cl_2 and quenched by the addition of saturated aqueous $NaHCO_3$, and diluted with H_2O. The aqueous phase was extracted with CH_2Cl_2 (3x 200 mL). The organic layer was washed with H_2O and brine, dried over $MgSO_4$, and filtered. The solvents were removed in vacuo and the residue was purified by flash column chromatography (silica gel, cyclohexane/ethyl acetate) to afford **S11** (17.7g, 35.2 mmol). R_f = 0.44 (hexanes/ethyl acetate, 3:1); ^1H NMR (400 MHz, $CDCl_3$) δ 7.43 – 7.06 (m, 9H), 5.10 – 4.97 (m, 2H), 4.57 (3H), 4.19 – 4.13 (m, 2H), 3.70 (t, *J* = 9.2 Hz, 1H), 3.60 (ddd, *J* = 10.0, 4.7, 3.4 Hz, 1H), 2.34 (s, 3H), 2.07 (s, 3H), 2.05 (s, 3H), 1.96 (s, 3H).

All spectroscopic data are in good accordance with the literature [7].

4-Methylphenyl 4,6-*O*-benzylidene-3-*O*-benzyl-1-thio-β-D-glucopyranoside (S12).

Compound **S11** (6 g, 11.9 mmol) was suspended in MeOH (47.8 mL), and NaOMe (0.5 M in MeOH, 4.78 mL) was added. The reaction mixture was stirred for 5 d and quenched by the addition of Amberlite IR-120 (H^+ form). The mixture was filtered, and the solvent was evaporated in vacuo. The resulting solid was used in the next reaction step without further purification. *p*-Methylphenyl 3-*O*-benzyl-1-thio-β-D-glucopyranose (4.48 g, 11.9 mmol) was taken up in CH_3CN (119 mL), and benzaldehyde dimethyl acetal (3.6 mL, 23.8 mmol) and *p*-toluenesulfonic acid monohydrate (113 mg, 0.6 mmol) were added. A white precipitate was forming instantly. The mixture was stirred for 2 h, and then quenched by the addition of Et_3N. The solvent was removed in vacuo, and the crude product was purified by flash column chromatography (silica gel, hexanes/ethyl acetate) to give **S12** (4.37 g, 9.4 mmol) as a white solid. R_f = 0.49 (hexanes/ethyl acetate, 3:1). ^{1}H NMR (400 MHz, $CDCl_3$) δ 7.50 – 7.27 (m, 12H), 7.13 (d, *J* = 8.2 Hz, 2H), 5.56 (s, 1H), 4.94 (d, *J* = 11.5 Hz, 1H), 4.79 (d, *J* = 11.5 Hz, 1H), 4.56 (d, *J* = 9.7 Hz, 1H), 4.38 (dd, *J* = 10.5, 4.9 Hz, 1H), 3.78 (t, *J* = 10.3 Hz, 1H), 3.66 (m, 2H), 3.55 – 3.44 (m, 2H), 2.35 (s, 3H).

All spectroscopic data are in good accordance with the literature [8,9].

4-Methylphenyl 2-*O*-benzoyl-4,6-*O*-benzylidene-3-*O*-benzyl-1-thio-β-D-glucopyranoside (S13).

Compound **S12** (512 mg, 1.1 mmol) was dissolved in CH_2Cl_2 (5.5 ml), and DIC (429 µl, 2,8 mmol), benzoic acid (202 mg, 1.7 mmol) and DMAP (26,9 mg, 0.22 mmol) were added. The mixture was stirred for 4 d. The organic layer was washed with 1 M HCl, saturated aqueous $NaHCO_3$, dried over $MgSO_4$, filtered and concentrated in vacuo. The crude material was purified by flash column chromatography (silica gel, hexanes/ethyl acetate) to give **S13** (411 mg, 0.72 mmol) as a white solid. R_f = 0.56 (hexanes/ethyl acetate, 3:1); ^{1}H NMR (400 MHz, $CDCl_3$) δ 8.12 – 7.97 (m, 2H), 7.66 – 7.59 (m, 1H), 7.54 – 7.33 (m, 9H), 7.17 – 7.05 (m, 7H), 5.61 (s, 1H), 5.28 (dd, *J* = 10.0, 8.6 Hz, 1H), 4.86 – 4.77 (m, 2H), 4.68 (d, *J* = 11.9 Hz, 1H), 4.43 (dd, *J* = 10.5, 5.0 Hz, 1H), 3.93 – 3.78 (m, 3H), 3.56 (td, *J* = 9.9, 5.0 Hz, 1H), 2.34 (s, 3H).

All spectroscopic data are in good accordance with the literature [8,9].

(4-Methylphenyl) 2-*O*-benzoyl-3,6-di-*O*-benzyl-1-thio-β-D-glucopyranoside (S14).

Compound **S13** (298 mg, 0.52 mmol) was coevaporated with toluene and dissolved in CH_2Cl_2 (10.3 mL) under argon. Et_3SiH (502 µl, 3,1 mmol) was added, and the reaction mixture was cooled to 0 °C. TFAA (81 µl, 0,58 mmol) and TFA (161 µl, 2,1 mmol) were added dropwise, and the reaction was warmed to room temperature and stirred overnight. Upon completion, the mixture was cooled to 0 °C and neutralized with saturated aqueous $NaHCO_3$, extracted with CH_2Cl_2, dried over $MgSO_4$ and filtered. The solvent was evaporated in vacuo and the crude material was purified by flash column chromatography (silica gel, hexanes/ethyl acetate) to afford **S14** (261 mg, 0.46 mmol). R_f = 0.36 (hexanes/ethyl acetate, 3:1); ^{1}H NMR (400 MHz, $CDCl_3$) δ 8.07 (dd, *J* = 8.2, 1.1 Hz, 2H), 7.63 – 7.57 (m, 1H), 7.47 (dd, *J* = 10.6, 4.8 Hz, 2H), 7.40 – 7.29 (m, 7H), 7.18 (s, 5H), 7.03 (d, *J* = 7.9 Hz, 2H), 5.24 (dd, *J* = 9.9, 9.1 Hz, 1H), 4.77 – 4.55 (m, 5H), 3.85 – 3.74 (m, 3H), 3.69 (t, *J* = 8.9 Hz, 1H), 3.58 (dt, *J* = 9.4, 4.7 Hz, 1H), 2.29 (s, 3H). ^{13}C NMR (101 MHz, $CDCl_3$) δ 165.3, 138.3, 137.9, 137.9, 133.4, 133.3, 130.0, 130.0, 130.0, 129.7, 129.0, 128.6, 128.5, 128.2, 128.0, 127.9, 127.9, 86.7, 83.8, 78.5, 74.8, 73.9, 72.3, 71.9, 70.5, 21.3.

All spectroscopic data are in good accordance with the literature [8,9].

4-Methylphenyl 2-*O*-benzoyl-3,6-di-*O*-benzyl-4-*O*-levulinoyl-1-thio-β-D-glucopyranoside (S15).

Compound **S14** (233 mg, 0.41 mmol) was dissolved in CH_2Cl_2 (8.2 mL), and DIC (127 µl, 0.82 mmol), levulinic acid (41 µL, 0.41 mmol) and DMAP (10 mg, 0,08 mmol) were added. The mixture was stirred for 2 d. Upon completion, the organic layer was washed with 1 M HCl, saturated aqueous $NaHCO_3$, dried over $MgSO_4$, filtered and concentrated in vacuo. The crude product was purified by flash column chromatography (silica gel, hexanes/ethyl acetate) to afford **S15** (247 mg, 0.37 mmol) as a white solid. R_f = 0.20 (hexanes/ethyl acetate, 3:1); ^{1}H NMR (400 MHz, $CDCl_3$) δ 8.06 – 8.01 (m, 2H), 7.63 – 7.57 (m, 1H), 7.50 – 7.43 (m, 2H), 7.39 – 7.27 (m, 7H), 7.16 – 7.07 (m, 5H), 7.00 (d, *J* = 7.9 Hz, 2H), 5.27 (dd, *J* = 10.0, 9.1 Hz, 1H), 5.09 (t, *J* = 9.6 Hz, 1H), 4.76 (d, *J* = 10.0 Hz, 1H), 4.58 – 4.52 (m, 4H), 3.87 (t, *J* = 9.2 Hz, 1H), 3.73 – 3.67 (m, 1H), 3.65 – 3.62 (m, 2H), 2.69 – 2.52 (m, 2H), 2.48 – 2.30 (m, 2H), 2.29 (s, 3H), 2.13 (s, 3H). ^{13}C NMR (101 MHz, $CDCl_3$) δ 171.6, 165.1, 138.3, 138.3, 137.8,

133.4, 133.2, 130.0, 129.9, 129.8, 129.0, 128.6, 128.5, 128.3, 128.0, 127.9, 127.8, 127.7, 86.7, 81.6, 78.0, 77.4, 74.3, 73.7, 72.3, 71.1, 69.9, 37.9, 29.9, 28.0, 21.3.

All spectroscopic data are in good accordance with the literature [10].

Dibutyl 2-*O*-Benzoyl-3,6-di-*O*-benzyl-4-*O*-levulinoyl-β-D-glucopyranosyl phosphate (3).

Compound **S15** (92 mg, 0.14 mmol)) was dissolved in CH_2Cl_2 (1.5 mL). 4 Å MS was added, and the mixture was stirred at room temperature for 10 min. Dibutyl hydrogen phosphate (82 µL, 0.41 mmol) was added, the mixture was stirred for 10 min and then cooled to 0 °C. NIS (37.1 mg, 0.17 mmol) was added at 0 °C and the mixture was stirred for 20 min. A suspension of TfOH in CH_2Cl_2 was added dropwise until the color changed to dark red. The reaction mixture was stirred for 90 min. Upon completion, the reaction was quenched by the addition of saturated aqueous $NaHCO_3$ and saturated aqueous $Na_2S_2O_3$ and extracted with CH_2Cl_2. The organic layer was dried over $MgSO_4$, filtered and concentrated in vacuo. The crude product was purified by flash column chromatography (silica gel, toluene/acetone) to afford **3** (82 mg, 0.11 mmol) as a white solid. R_f = 0.30 (hexanes/ethyl acetate, 1:1); $[\alpha]_D^{20}$ = 29.70 (c = 0.5, $CHCl_3$); IR (thin film, cm^{-1}): 2963.3, 1731.5, 1720.5, 1451.2, 1267.1, 1030.9; ^1H NMR (400 MHz, $CDCl_3$) δ 8.05 – 7.99 (m, 2H), 7.61 – 7.55 (m, 1H), 7.47 – 7.41 (m, 2H), 7.34 – 7.26 (m, 5H), 7.16 – 7.10 (m, 5H), 5.43 – 5.36 (m, 2H), 5.29 – 5.20 (m, 1H), 4.65 – 4.45 (m, 4H), 4.06 – 3.95 (m, 2H), 3.90 – 3.82 (m, 1H), 3.80 – 3.56 (m, 5H), 2.67 – 2.52 (m, 2H), 2.48 – 2.28 (m, 2H), 2.13 (s, 3H), 1.60 – 1.49 (m, 2H), 1.38 – 1.19 (m, 4H), 1.07 – 0.94 (m, 2H), 0.85 (t, *J* = 7.4 Hz, 3H), 0.66 (t, *J* = 7.4 Hz, 3H); ^{13}C NMR (101 MHz, $CDCl_3$) δ 206.3, 171.5, 165.0, 138.0, 137.6, 133.5, 130.0, 129.4, 128.6, 128.4, 128.4, 128.1, 127.9, 127.8, 127.8, 96.7 (d, *J* = 4.9 Hz), 79.5, 77.4, 74.2, 74.1, 73.7, 73.1, 73.1, 70.7, 69.2, 68.2, 68.1, 68.0, 68.0, 37.8, 32.2, 32.1, 31.9, 31.8, 29.9, 29.7, 28.0, 18.7, 18.4, 13.7, 13.5; ^{31}P NMR (162 MHz, $CDCl_3$) δ -2.77. HRMS calcd for $C_{40}H_{51}O_{12}PNa$ $[M + Na]^+$, 777.3010 ; found, 777.3028.

Automated solid-phase synthesis of SP-3 CPS antigens:

Solution A, building block **2** (3 × 3 equiv per glycosylation cycle)

Solution B, building block **1** (3 × 3 equiv per glycosylation cycle)

Solution C, activator (3 × 3 equiv per glycosylation cycle): TMSOTf in CH_2Cl_2

Solution D, Fmoc deprotection: Et_3N (10% v/v) in DMF

Solution E, Lev deprotection: N_2H_4·OAc in pyridine/acetic acid (4:1 v/v), 7.8 equiv in 2.5 mL

Module A: Glycosylation. **Solution A** (1 mL) is added to the reaction vessel, and the suspension is cooled to −30 °C while the resin is being agitated by an argon flow. Upon reaching the set temperature, **solution C** (1 mL) is added to the reaction vessel. The temperature is maintained for 30 min and then raised to −15 °C for 30 min. The reaction vessel is then drained by argon pressure and the resin is washed with CH_2Cl_2 (6×).

Module B: Glycosylation. **Solution B** (1 mL) is added to the reaction vessel, and the suspension is cooled to −30 °C while the resin is being agitated by an argon flow. Upon reaching the set temperature, **solution C** (1 mL) is added to the reaction vessel. The temperature is maintained for 30 min and then raised to −15 °C for 30 min. The reaction vessel is then drained by argon pressure and the resin is washed with CH_2Cl_2 (6×).

Module C: Swelling of resin prior to synthesis. The reaction vessel is charged with functionalized resin, and CH_2Cl_2 (2 mL) is added. The resin is swelled in CH_2Cl_2 for 2 h. At the beginning of the synthesis, the reaction vessel is drained by argon pressure.

Module D: Washing before glycosylation. The resin is washed with THF (6×) and CH_2Cl_2 (6×) at room temperature.

Module E: Activator wash. **Solution C** is added at −30 °C for 1 min while the suspension is agitated by an argon flow. The reaction vessel is then drained by argon pressure, and the resin is washed with CH_2Cl_2 (6×).

Module F: Washing after glycosylation cycle. The temperature is raised to 25 °C, and the resin is washed with THF (6×) and CH_2Cl_2 (6×).

Module G: Fmoc deprotection. The resin is washed with DMF (3×). **Solution C** (2 mL) is added to the reaction vessel, and the suspension is agitated by an argon flow for 15 min at 25 °C. The reaction vessel is then drained and the solvents are transferred to a fraction collector via argon pressure.

Module H: Washing after Fmoc deprotection. The temperature is set to 25 °C, and the resin is washed with DMF (3×), CH_2Cl_2 (6×), AcOH in CH_2Cl_2 (6×), THF (6×), and CH_2Cl_2 (6×).

Module I: Lev deprotection. The resin is washed with DMF (3×). **Solution E** (2.5 mL) is added to the reaction vessel, and the temperature is increased to 40 °C. The suspension is agitated by argon bubbling for 10 min at 40 °C, then the reaction vessel is drained by argon pressure. The resin is washed with DMF (3×).

Module J: Washing after Lev deprotection. The temperature is set to 25 °C, and the resin is washed with CH_2Cl_2 (3×), THF (3×), acetone in CH_2Cl_2 (20% v/v, 6 x), CH_2Cl_2 (6×), AcOH in CH_2Cl_2 (6×), THF (6×), and CH_2Cl_2 (6×).

***N*-Benzylcarbamoylaminopentyl 2-*O*-benzoyl-3,6-di-*O*-benzyl-4-*O*-fluorenylmethoxycarbonyl-β-D-glucopyranosyl-(1→3)-methyl (2-*O*-benzoyl-4-*O*-benzyl-β-D-glucuropyranosyl)-(1→4)-2-*O*-benzoyl-3,6-di-*O*-benzyl-β-D-glucopyranoside (5).**

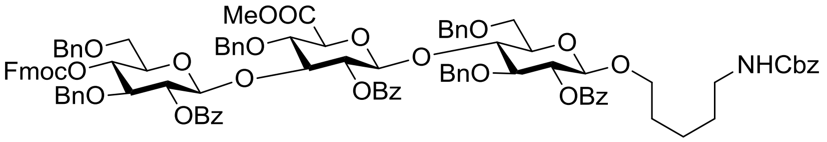

Glucose Unit	Modules (Iterations)
**1**	**D**(1), **A** (3), **F** (1), **G** (3), **H** (1), **E** (1)
**2**	**D**(1), **B** (3), **F** (1), **I** (2), **J** (1), **E** (1)
**3**	**D**(1), **A** (3), **F** (1)

Table S1. Program modules for the synthesis of SP-3 trisaccharide **5**.

The reaction vessel of the synthesizer was charged with functionalized Merrifield resin **4** (22 mg, loading: 0.27 mmol/g). Program module **C** was executed once. Then the program described in Table S1 was executed by the synthesizer. The resin was removed from the reaction vessel and swelled in CH_2Cl_2. The suspension was irradiated with UV light by

delivering the suspension via syringe pump through a FEP tubing (inner diameter: 0.03 inch; volume: 12 mL) wrapped around a UV light source (medium pressure Hg lamp with arc lengths of 27.9 cm and power of 450 W, surrounded by a Pyrex UV filter with 50% transmittance at 305 nm). The resin was slowly injected from a disposable syringe (2 mL) and pushed through the tubing with 15 mL CH_2Cl_2 (flow rate: 300 μL·min^{-1}). The tubing was washed with 15 mL CH_2Cl_2/MeOH (1:1 v/v, flow rate: 300 μL·min^{-1} for 8 mL and 4 mL·min^{-1} for 7 mL), and finally with 15 mL MeOH (flow rate: 4 mL·min^{-1}). The suspension leaving the reactor was directed into a filter where the resin was filtered off and washed with CH_2Cl_2/MeOH (1:1 v/v), MeOH and CH_2Cl_2, while the filtrate was collected. The tubing was re-equilibrated with 15 mL CH_2Cl_2 using a flow rate of 4 mL·min^{-1}. The entire cleavage procedure was repeated three times. The resulting solution was concentrated in vacuo and purified by flash column chromatography (silica gel, hexane/ethyl acetate) to afford **5** (7.1 mg, 4.10 μmol) as a colorless solid. $[\alpha]_D^{20}$ = +24.30 (c = 1.0, $CHCl_3$); IR (thin film, cm^{-1}): 2924, 2865, 1729, 1602, 1452, 1249, 1069, 740; ^{1}H NMR (400 MHz, $CDCl_3$) δ 7.86 (d, *J* = 8.3 Hz, 2H), 7.78 – 7.64 (m, 7H), 7.61 – 7.46 (m, 7H), 7.43 – 7.15 (m, 27H), 7.13 – 6.87 (m, 10H), 5.26 (t, *J* = 8.7 Hz, 1H), 5.19 – 4.80 (m, 7H), 4.71 – 4.56 (m, 4H), 4.54 – 4.19 (m, 10H), 4.15 – 3.82 (m, 5H), 3.76 – 3.42 (m, 10H), 3.35 – 3.19 (m, 2H), 3.04 (d, *J* = 9.4 Hz, 1H), 2.92 – 2.79 (m, 2H), 1.48 – 1.28 (m, 4H), 1.17 – 1.01 (m, 2H); ^{13}C NMR (101 MHz, $CDCl_3$) δ 168.2, 165.1, 165.1, 163.9, 156.4, 154.3, 143.3, 143.1, 141.4, 141.4, 138.6, 138.3, 138.1, 138.0, 137.3, 136.8, 133.7, 133.1, 133.0, 130.1, 130.1, 129.9, 129.8, 129.6, 129.2, 128.8, 128.7, 128.6, 128.5, 128.4, 128.4, 128.4, 128.3, 128.3, 128.2, 128.2, 128.1, 128.1, 128.0, 127.8, 127.8, 127.7, 127.3, 127.1, 125.2, 125.1, 120.2, 101.2, 100.8, 100.5, 80.6, 79.9, 79.4, 77.4, 76.0, 75.2, 74.8, 74.8, 74.7, 74.3, 73.9, 73.8, 73.7, 73.5, 73.4, 73.2, 70.2, 70.2, 69.4, 67.3, 66.6, 52.5, 46.8, 40.9, 29.5, 28.9, 23.2; HRMS calcd for $C_{103}H_{101}NO_{24}Na$ [M + Na]$^+$, 1759.6640; found, 1759.6680.

5-Amino-pentyl β-D-glucopyranosyl-(1→3)-β-D-glucuropyranosyl-(1→4)-β-D-glucopyranoside (11).

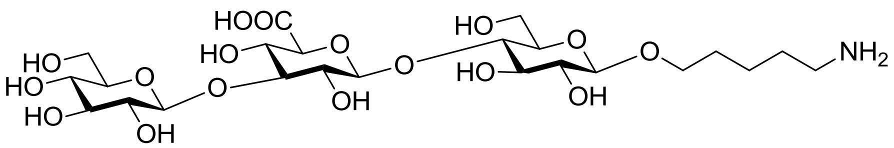

Trisaccharide **5** (7.1 mg, 4.10 µmol) was dissolved in THF (0.41 mL) and cooled to −5 °C. H_2O_2 (0.140 mmol, 14 µL) was added dropwise, and with vigorous stirring LiOH (1 M, 29 µL) was added dropwise. The solution was stirred for 2 h during which time the temperature went to 0 °C. The reaction was stirred for another hour at 0 °C and then warmed to rt and stirred overnight. The solution was then cooled to 0 °C, and NaOH (1 M, 62 µL) was added dropwise, followed by the addition of MeOH (135 µL). The reaction was warmed to room temperature and stirred overnight. The reaction was neutralized with Amberlite IR-120 (H^+ form), filtered, and the solvents were evaporated in vacuo. The residue was dissolved in a mixture of MeOH/H_2O/AcOH (50:25:1, v/v/v, 0.005 M) under argon, and $Pd(OH)_2$/C (12 mg, 20 wt. %) was added. The atmosphere was exchanged with H_2, and the reaction was stirred at rt overnight. The reaction mixture was filtered and concentrated in vacuo. The residue was purified by gel permeation chromatography (Sephadex LH-20, H_2O) and lyophilized to afford **11** (1.8 mg, 2.91 µmol) as a white solid. ^{1}H NMR (400 MHz, D_2O) δ 4.85 (d, *J* = 7.9 Hz, 1H), 4.60 (d, *J* = 8.0 Hz, 1H), 4.54 (d, *J* = 8.0 Hz, 1H), 4.05 – 3.96 (m, 3H), 3.88 – 3.82 (m, 3H), 3.80 – 3.61 (m, 7H), 3.57 (t, *J* = 9.2 Hz, 1H), 3.54 – 3.50 (m, 1H), 3.48 – 3.44 (m, 1H), 3.41 (dd, *J* = 9.4, 8.0 Hz, 1H), 3.38 – 3.34 (m, 1H), 3.09 – 3.03 (m, 2H), 1.79 – 1.67 (m, 4H), 1.56 – 1.47 (m, 2H); ^{13}C NMR (101 MHz, D_2O) δ 175.2, 102.5, 102.0, 101.9, 82.7, 78.8, 76.0, 75.7, 75.5, 74.7, 74.3, 73.4, 73.0, 72.8, 70.1, 70.0, 69.5, 60.6, 60.0, 39.3, 28.1, 26.4, 22.0; HSQC (400 MHz, D_2O) δ 102.5 ($J_{C1,H1}$ = 164 Hz, C-1), 102.0 ($J_{C1,H1}$ = 161 Hz, C-1), 101.9 ($J_{C1,H1}$ = 162 Hz, C-1); HRMS calcd for $C_{23}H_{41}NO_{17}Na$ $[M + Na]^+$, 626.2267; found, 626.2276.

References

(1) Martin, C. E.; Weishaupt, M. W.; Seeberger, P. H. *Chem. Commun.* **2011**, *47*, 10260.
(2) Hofmann, J.; Hahm, H. S.; Seeberger, P. H.; Pagel, K. *Nature* **2015**, *526*, 241.
(3) Bichard, C. J. F.; Wheatley, J. R.; Fleet, G. W. J. *Tetrahedron: Asymmetry* **1994**, *5*, 431.
(4) Koeppen, B. H. *Carbohydr. Res.* **1972**, *24*, 154.
(5) Lenagh-Snow, G. M. J.; Araújo, N.; Jenkinson, S. F.; Martínez, R. F.; Shimada, Y.; Yu, C.-Y.; Kato, A.; Fleet, G. W. J. *Org. Lett.* **2012**, *14*, 2142.
(6) Takeo, K.; Kitamura, S.; Murata, Y. *Carbohydr. Res.* **1992**, *224*, 111.
(7) Polat, T.; Wong, C. H. *J. Am. Chem. Soc.* **2007**, *129*, 12795.
(8) Wang, C. C.; Kulkarni, S. S.; Lee, J. C.; Luo, S. Y.; Hung, S. C. *Nat. Protoc.* **2008**, *3*, 97.
(9) Wang, C.-C.; Lee, J.-C.; Luo, S.-Y.; Kulkarni, S. S.; Huang, Y.-W.; Lee, C.-C.; Chang, K.-L.; Hung, S.-C. *Nature* **2007**, *446*, 896.
(10) de Jong, A. R.; Hagen, B.; van der Ark, V.; Overkleeft, H. S.; Codee, J. D. C.; van der Marel, G. A. *J. Org. Chem.* **2012**, *77*, 108.

Compound **S2**: ^{1}H NMR

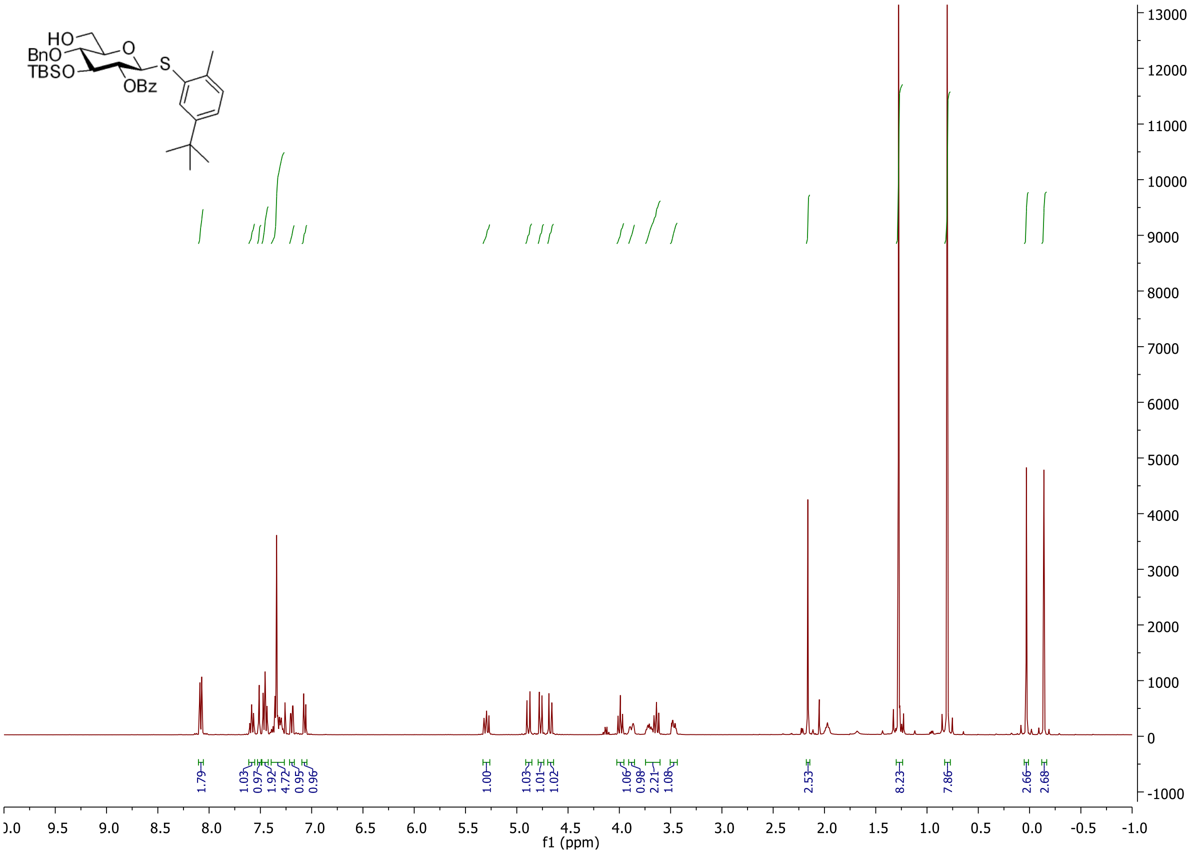

Compound **S2**: HH-COSY NMR

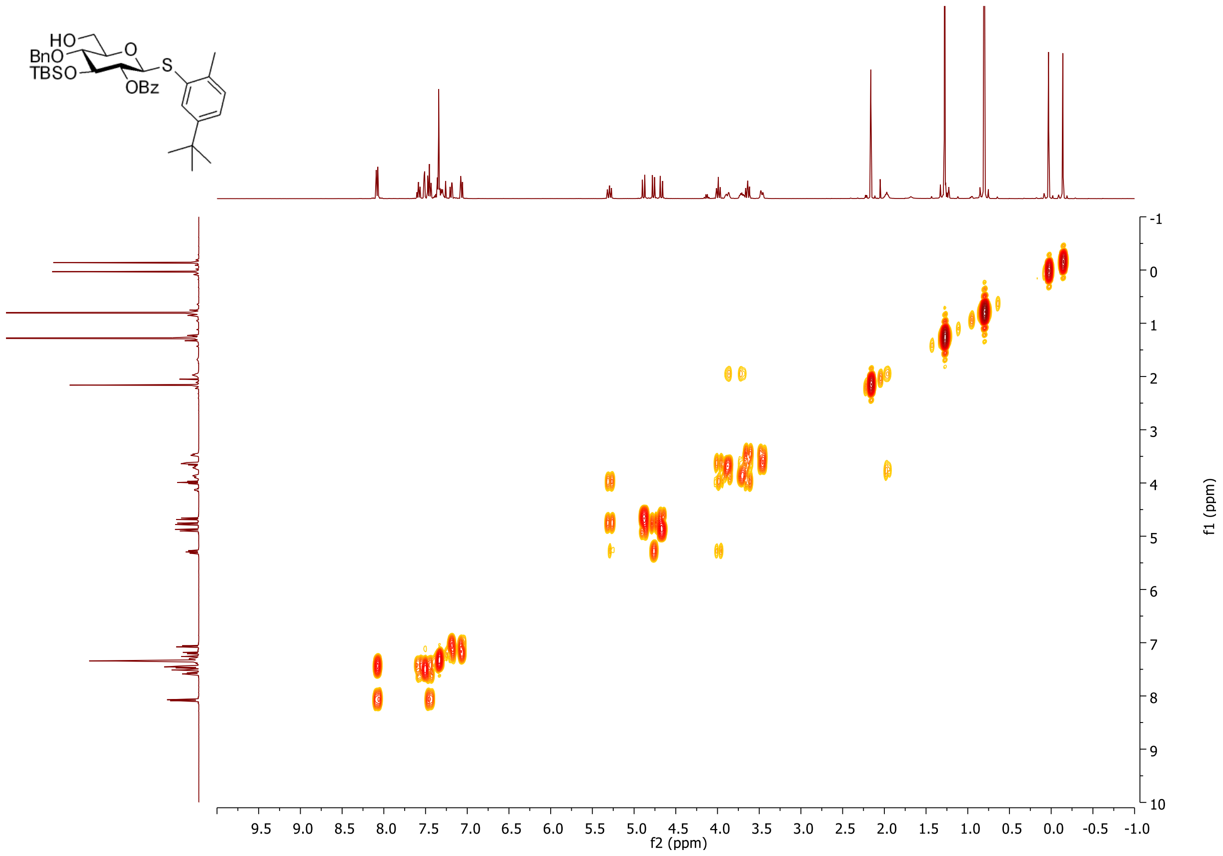

Compound **S2**: HSQC NMR

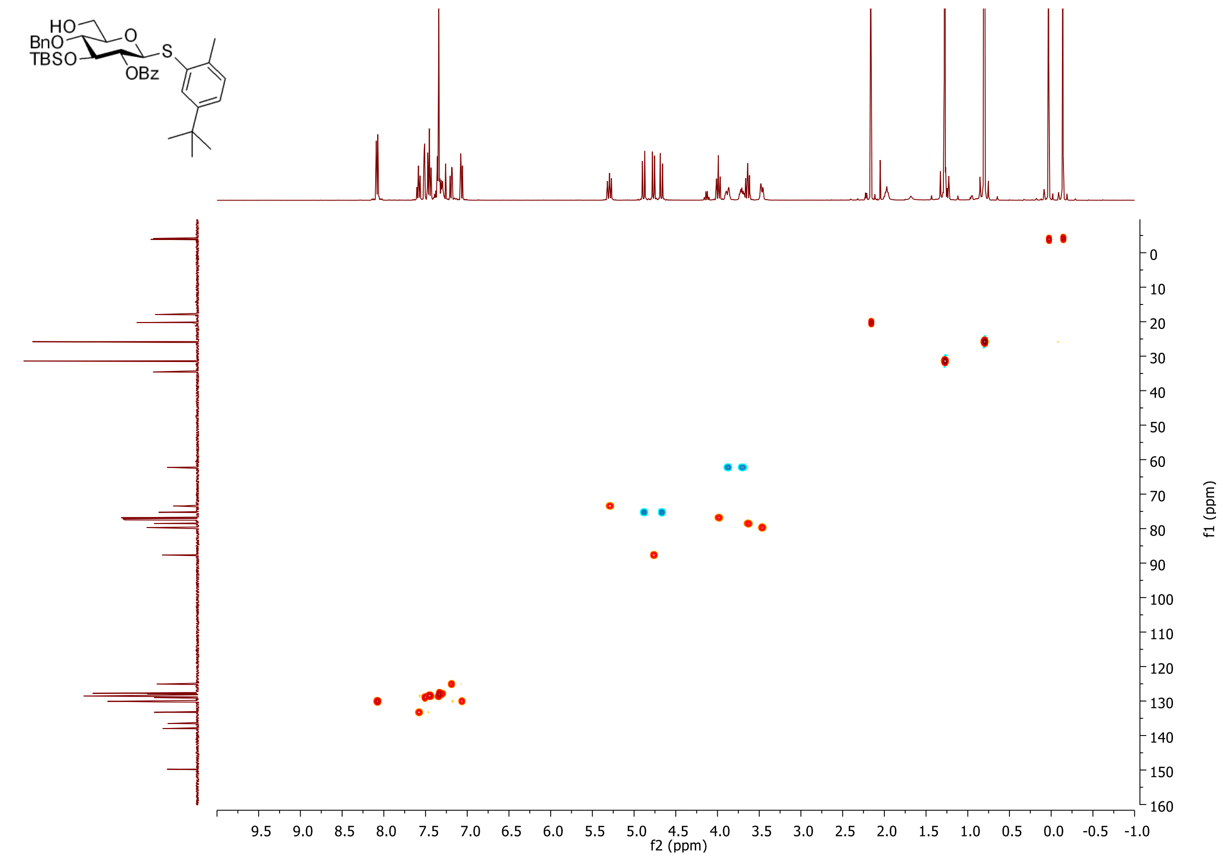

Compound **S2**: ^{13}C NMR

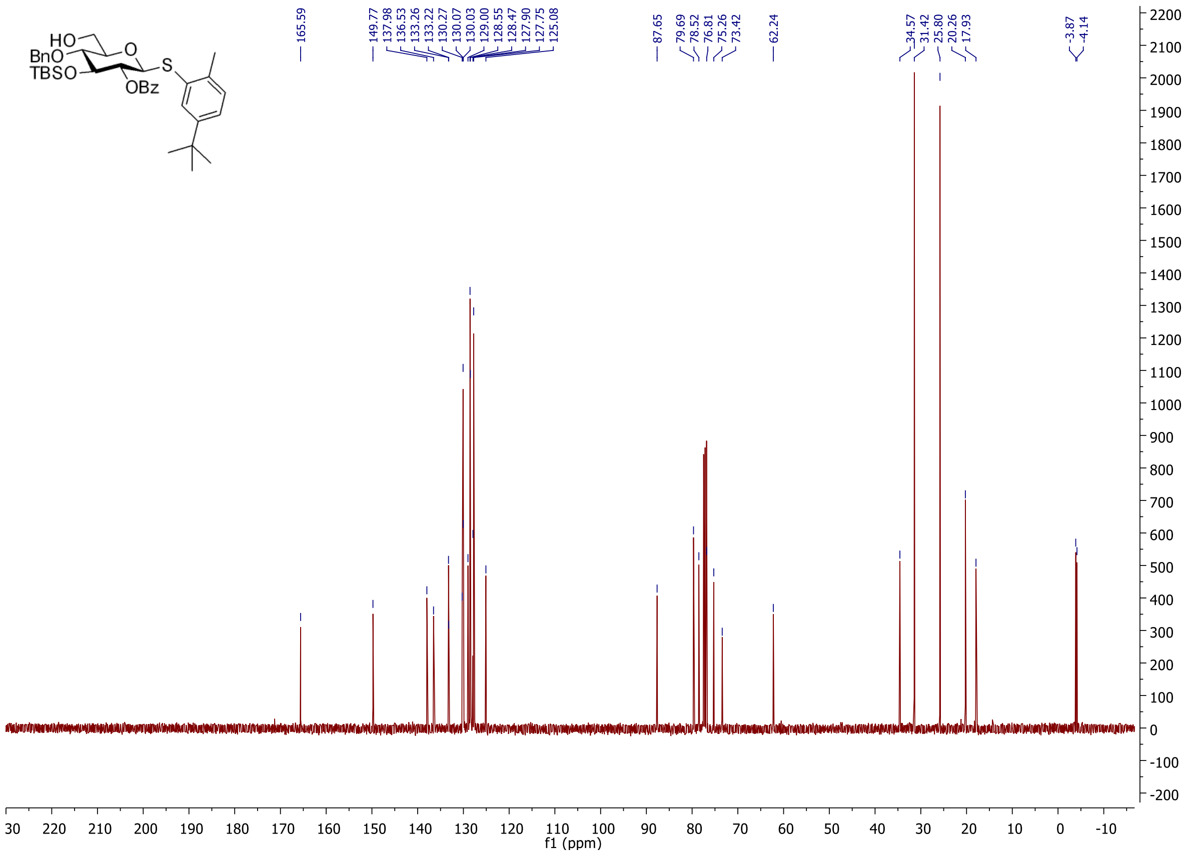

Compound **S3**: ^{1}H NMR

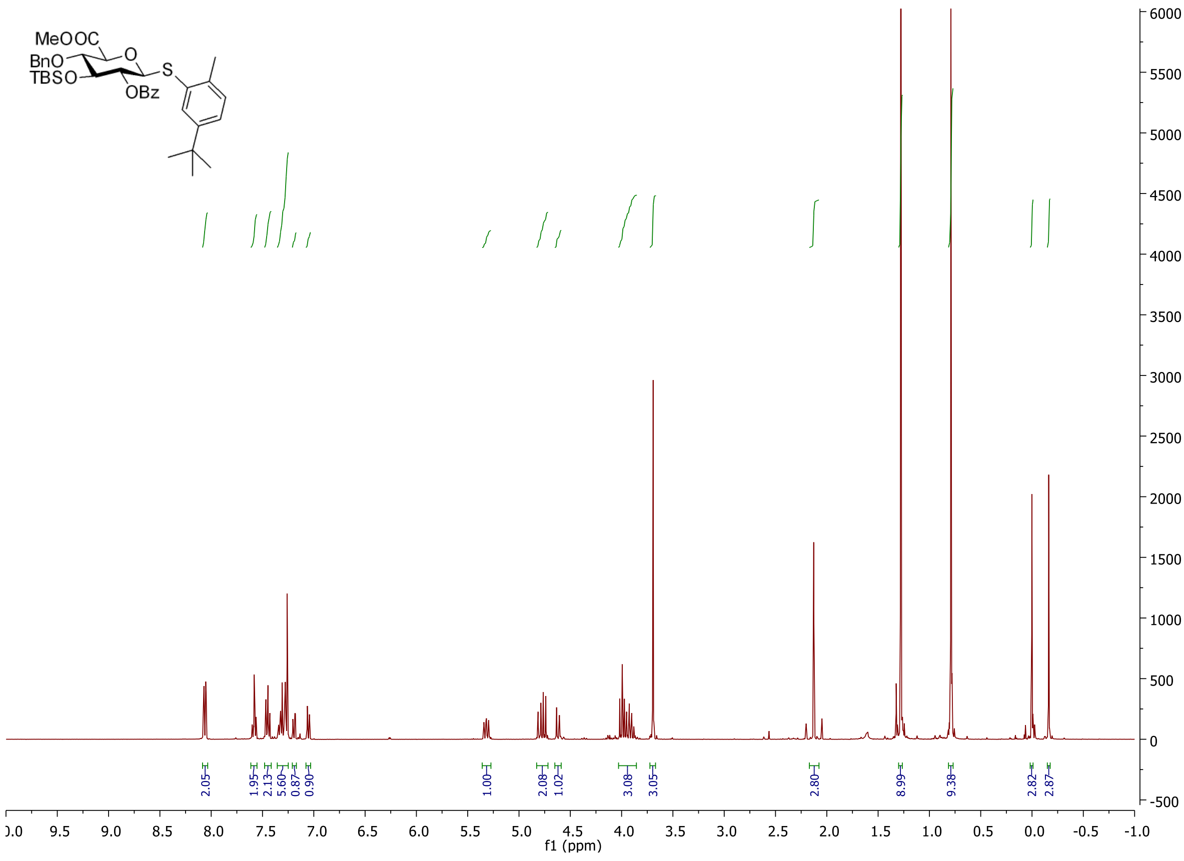

Compound **S3**: HH-COSY NMR

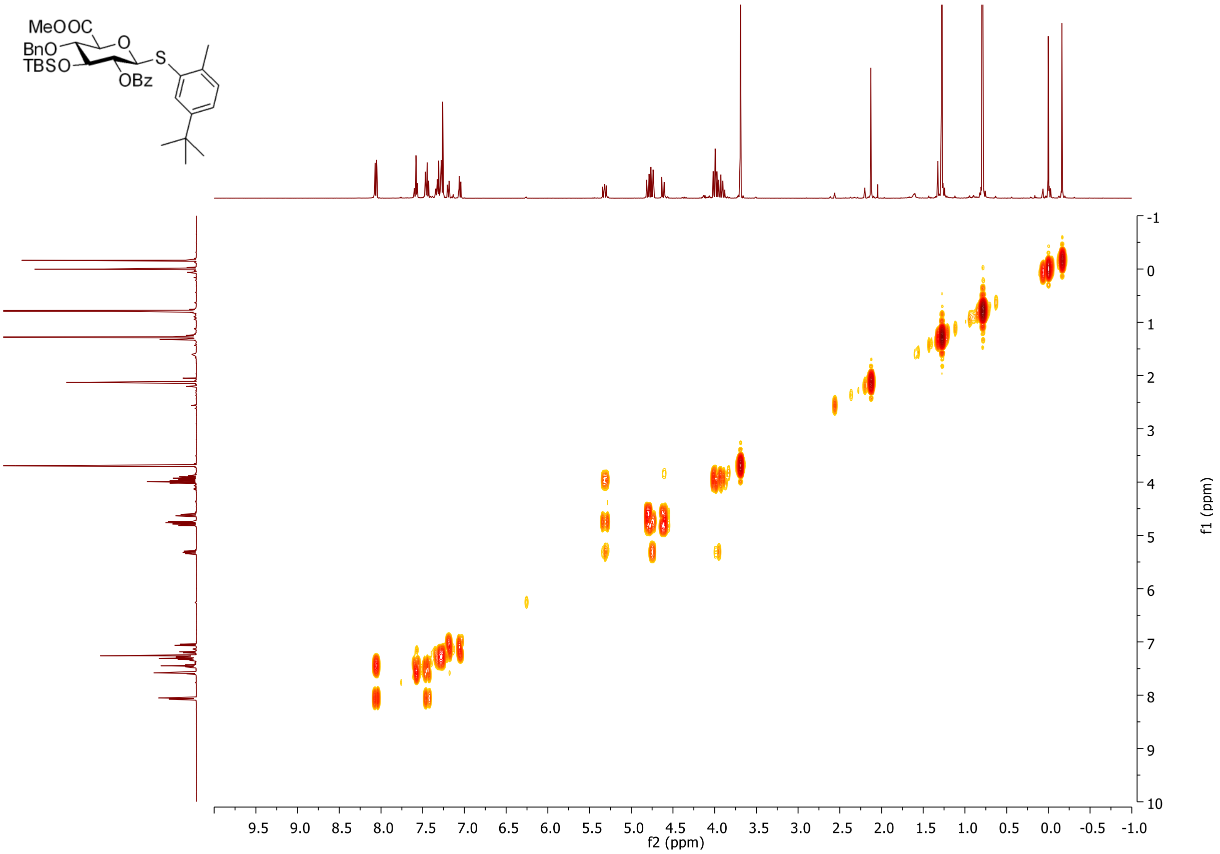

Compound **S3**: HSQC NMR

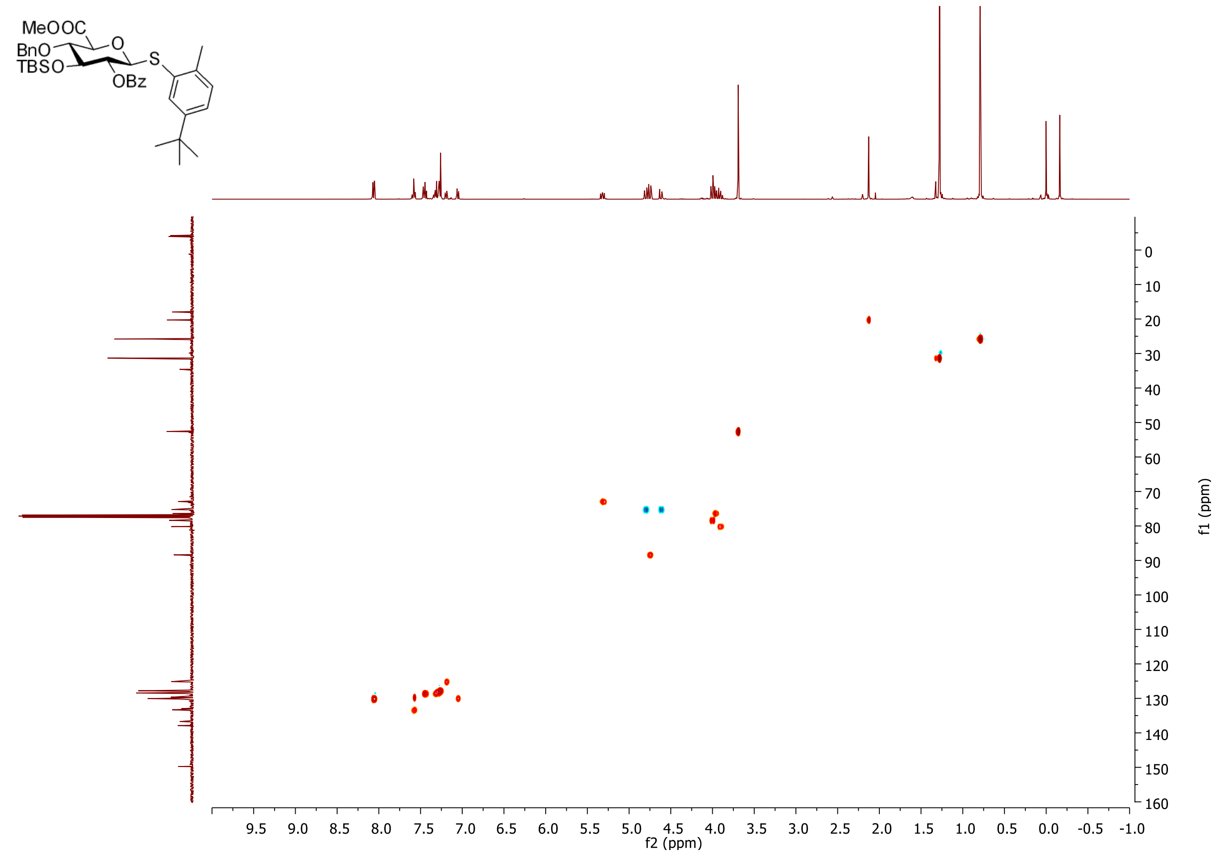

Compound **S3**: ^{13}C NMR

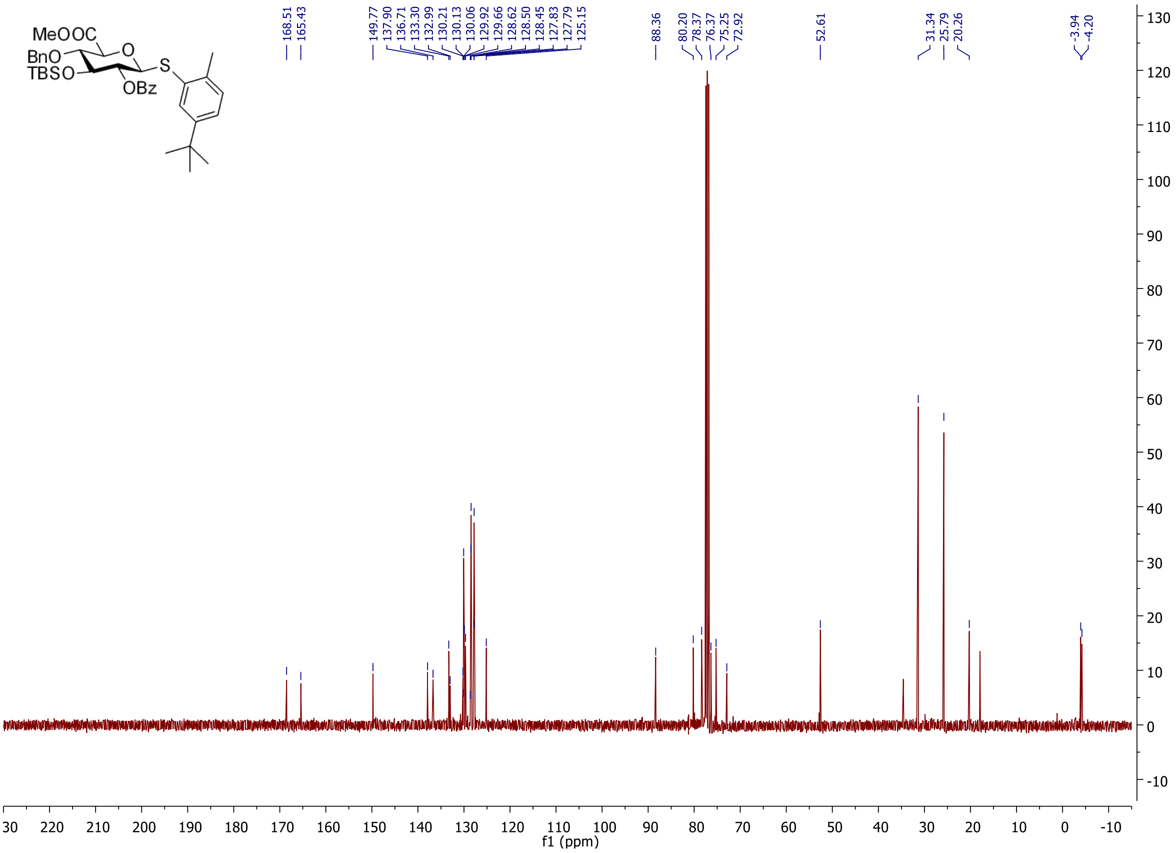

Compound **S4**: ^{1}H NMR

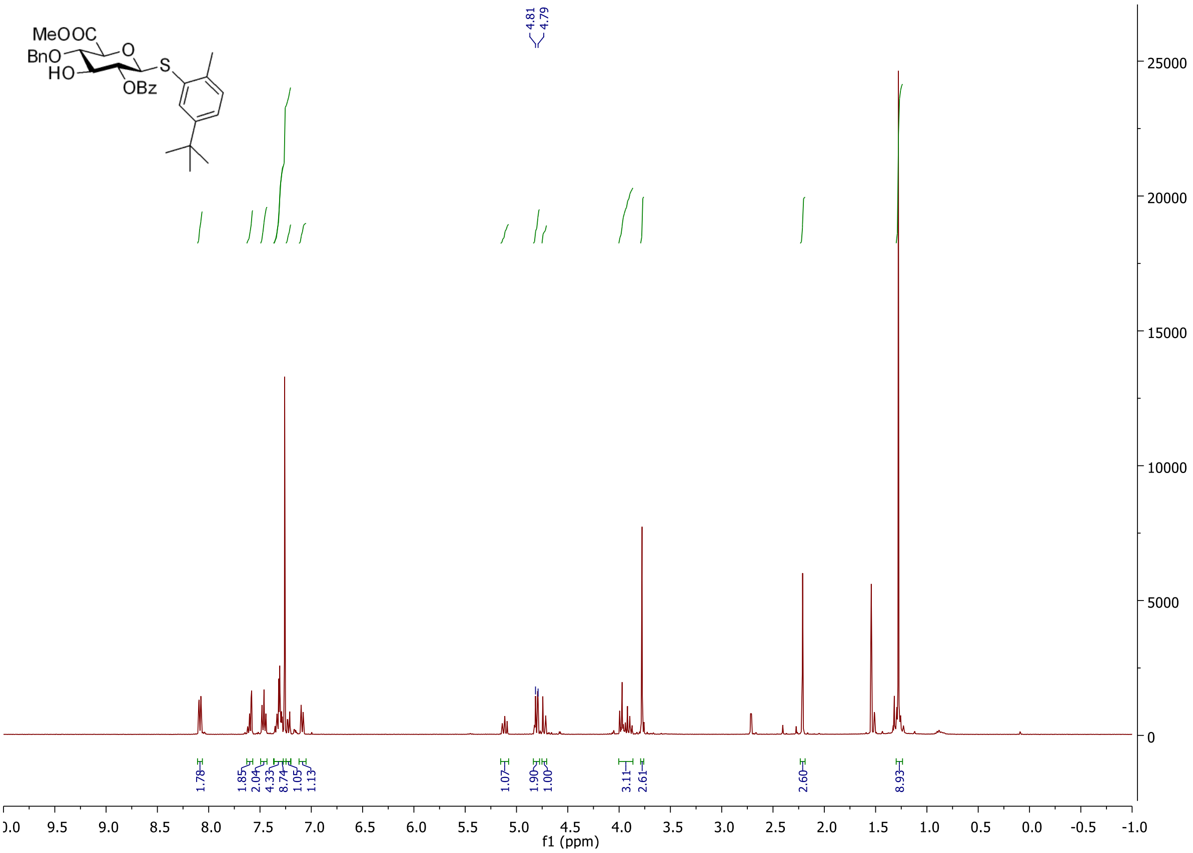

Compound **S4**: HH-COSY NMR

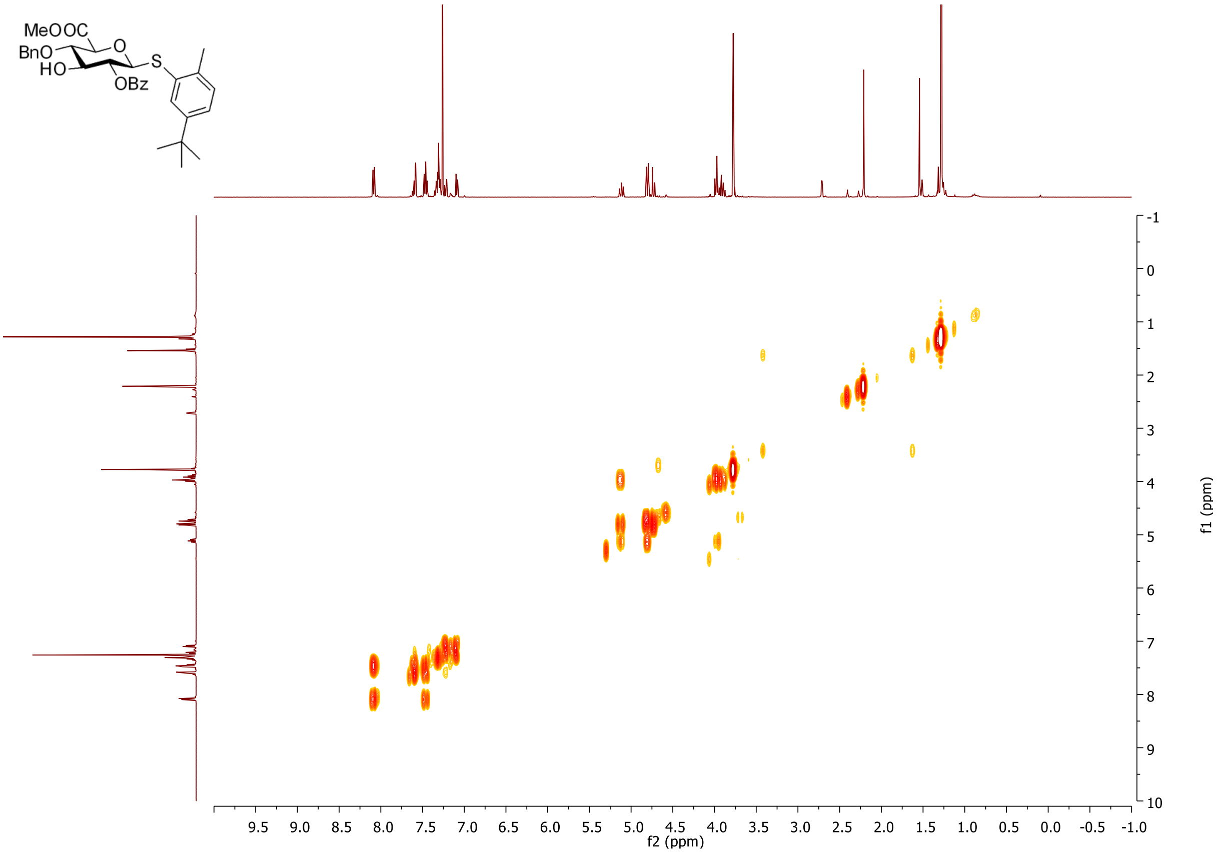

Compound **S4**: HSQC NMR

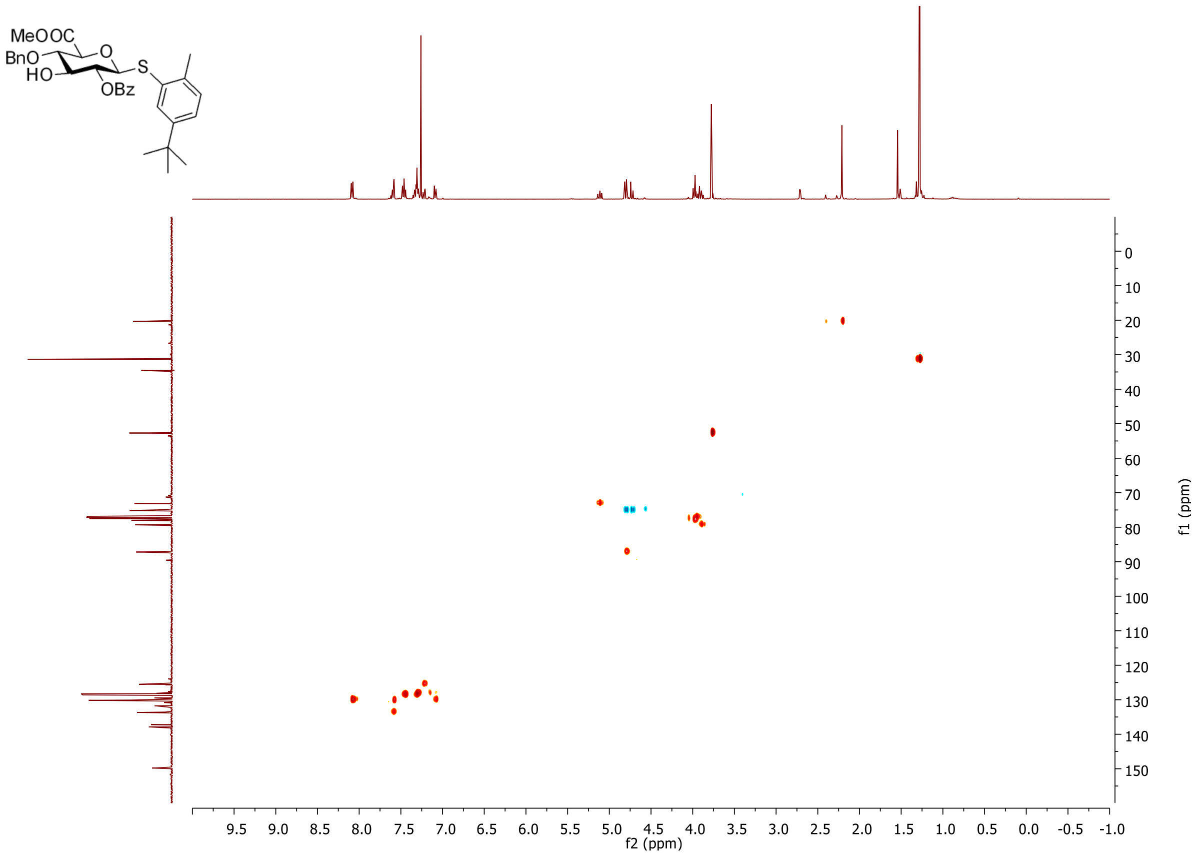

Compound **S4**: ^{13}C NMR

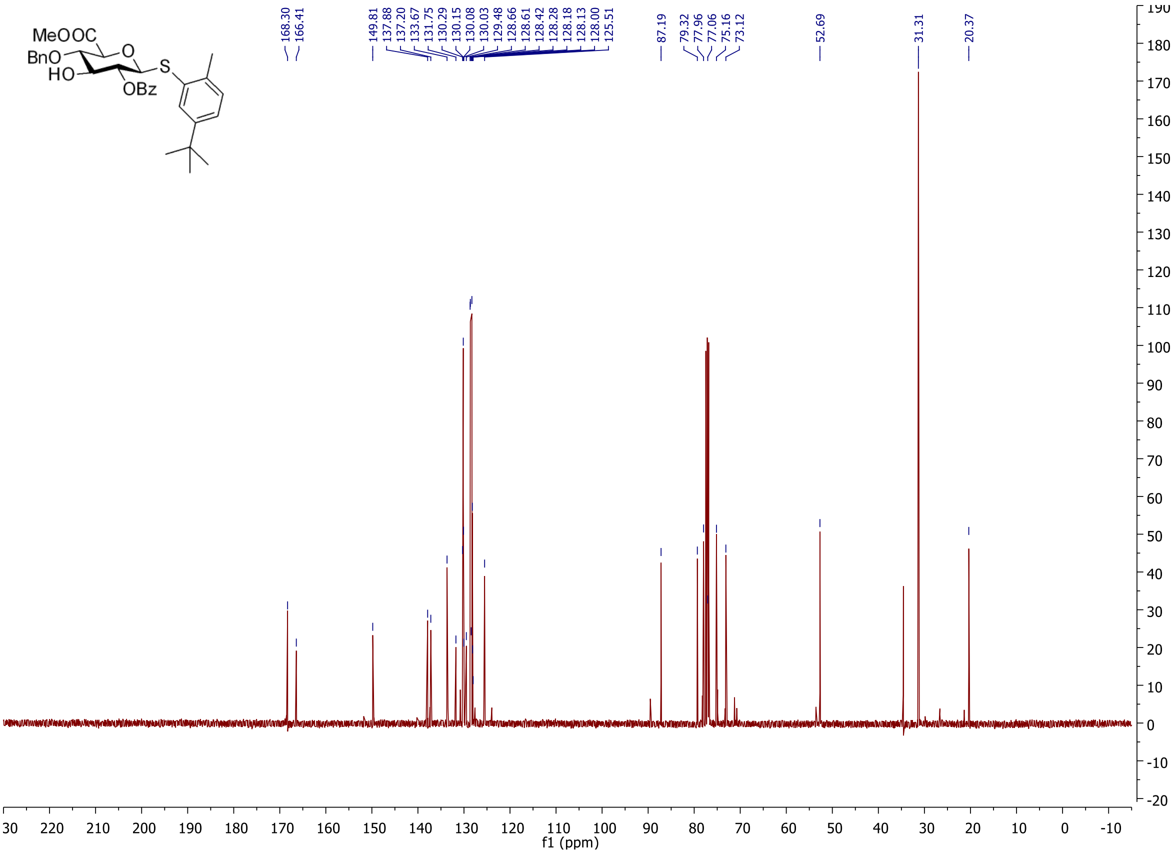

Compound **S5**: ^{1}H NMR

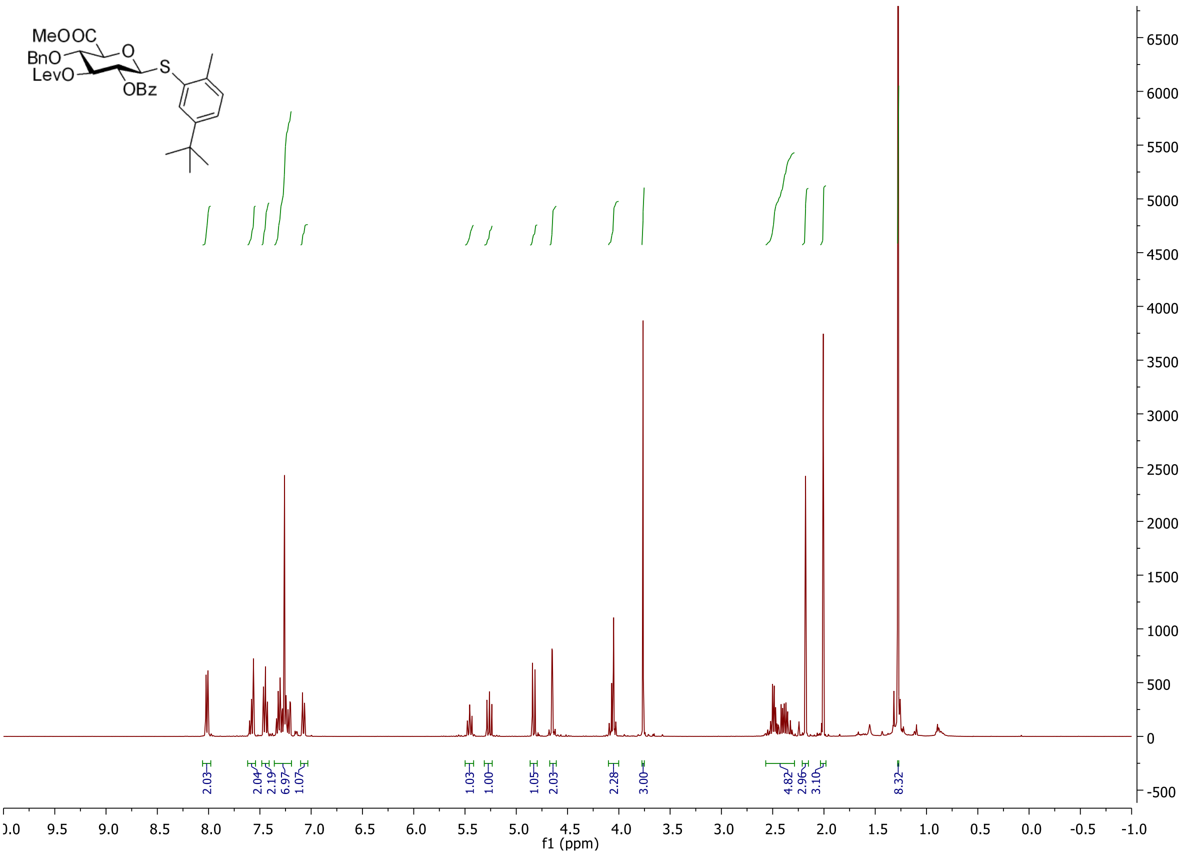

Compound **S5**: HH-COSY NMR

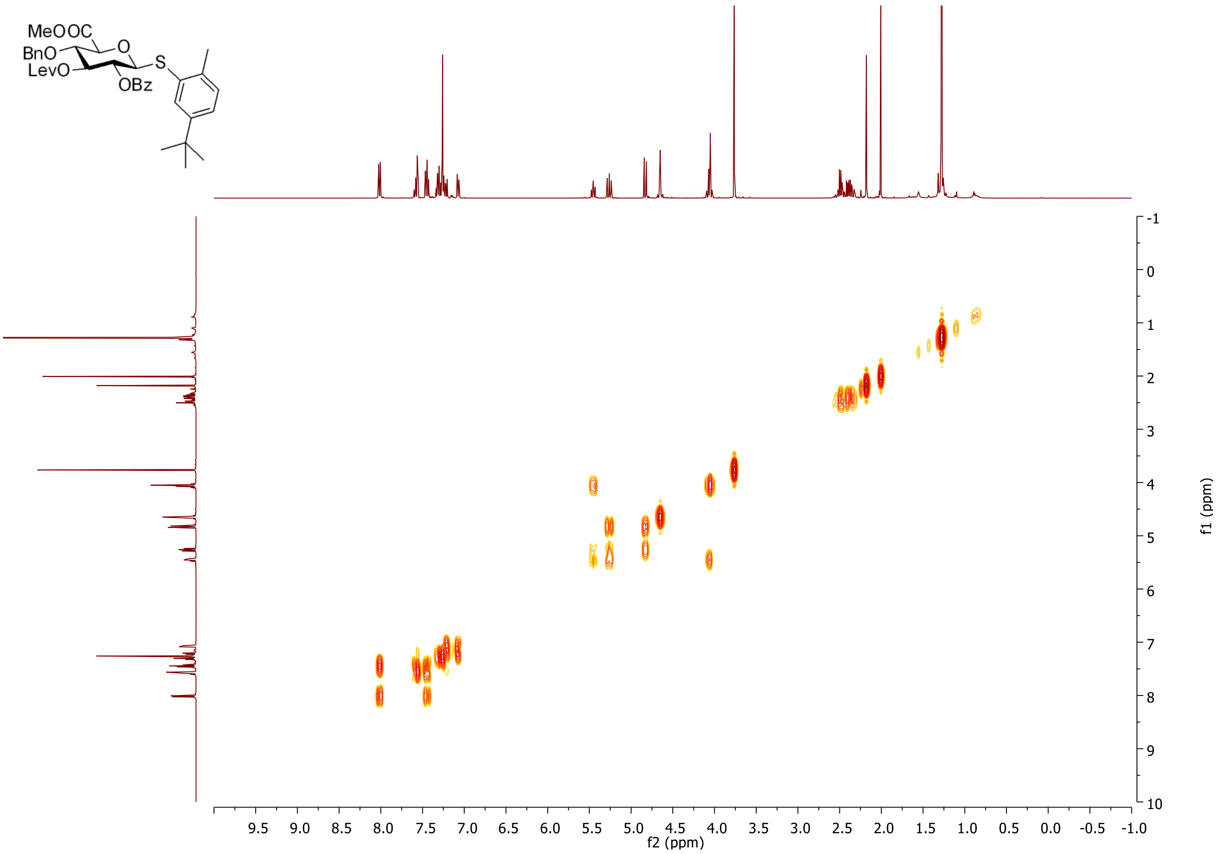

Compound **S5**: HSQC NMR

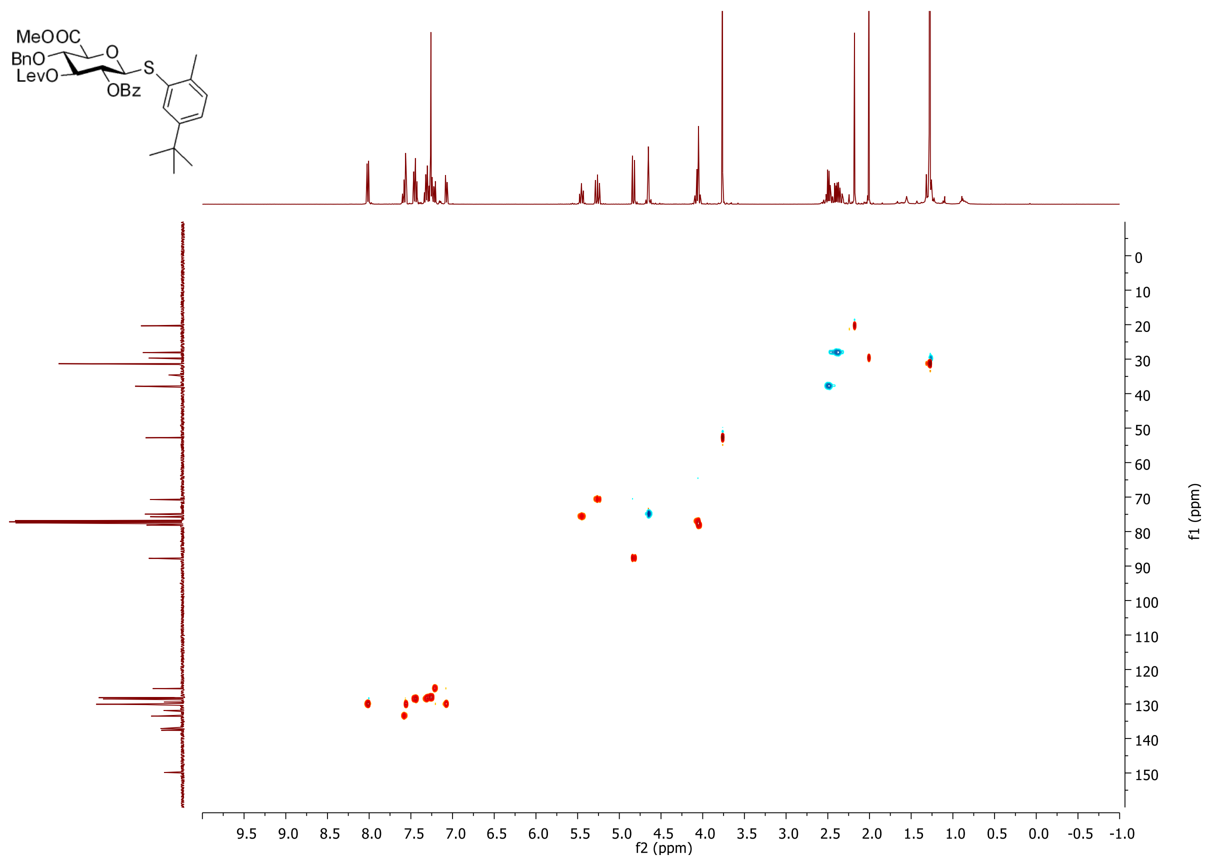

Compound **S5**: ^{13}C NMR

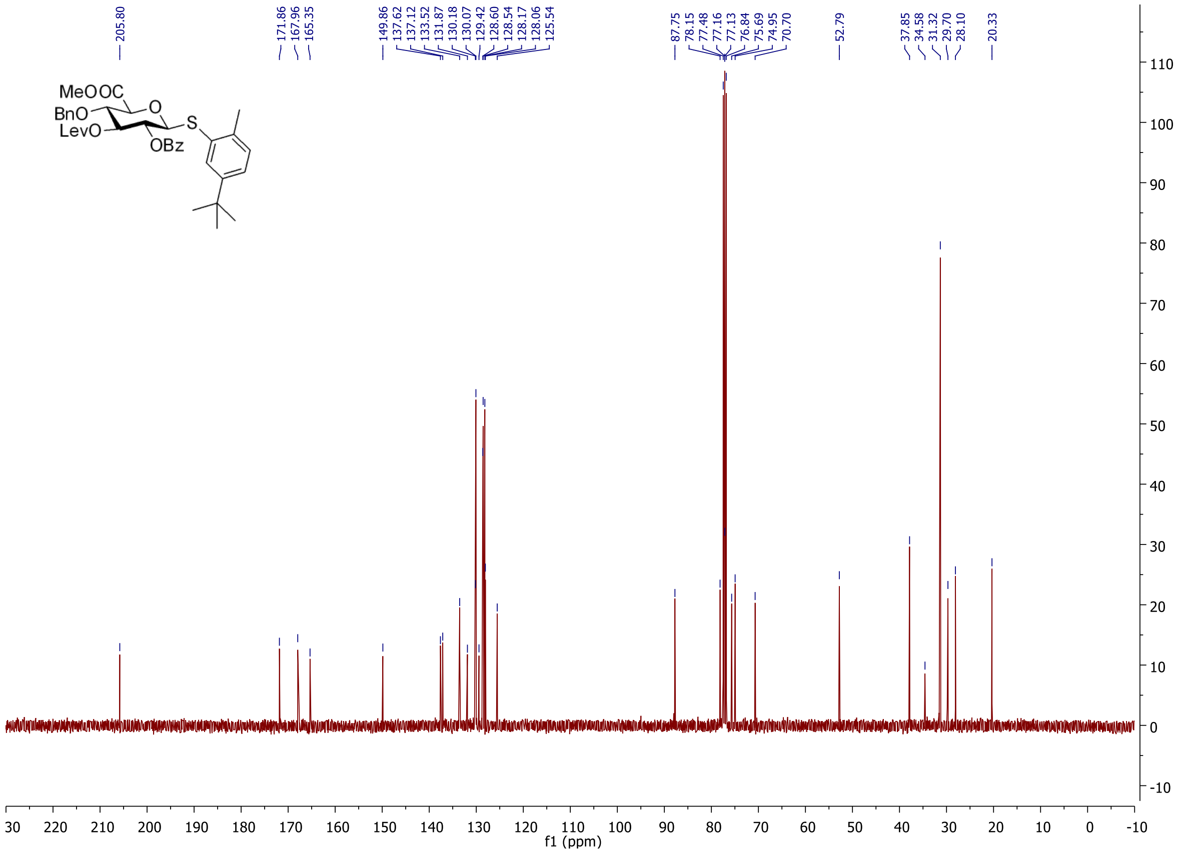

Compound **1**: ^{1}H NMR

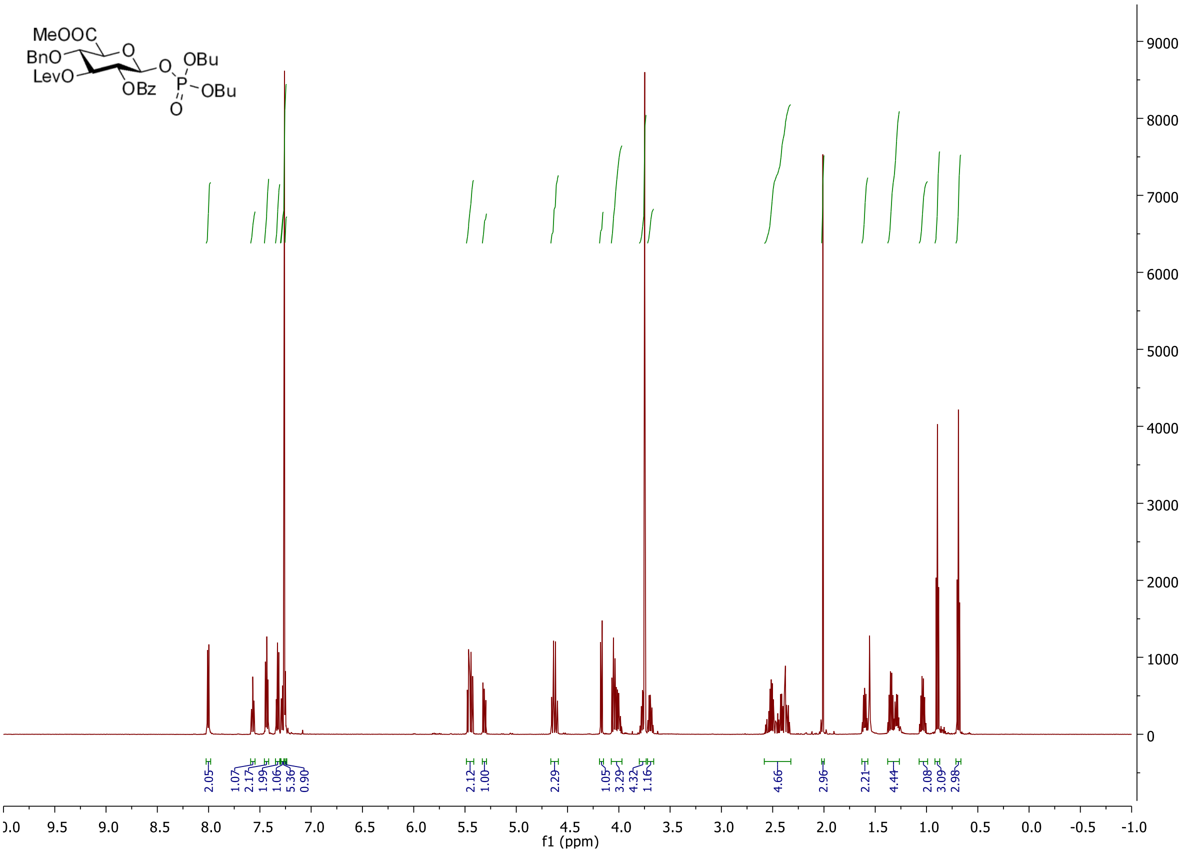

Compound **1**: HH-COSY NMR

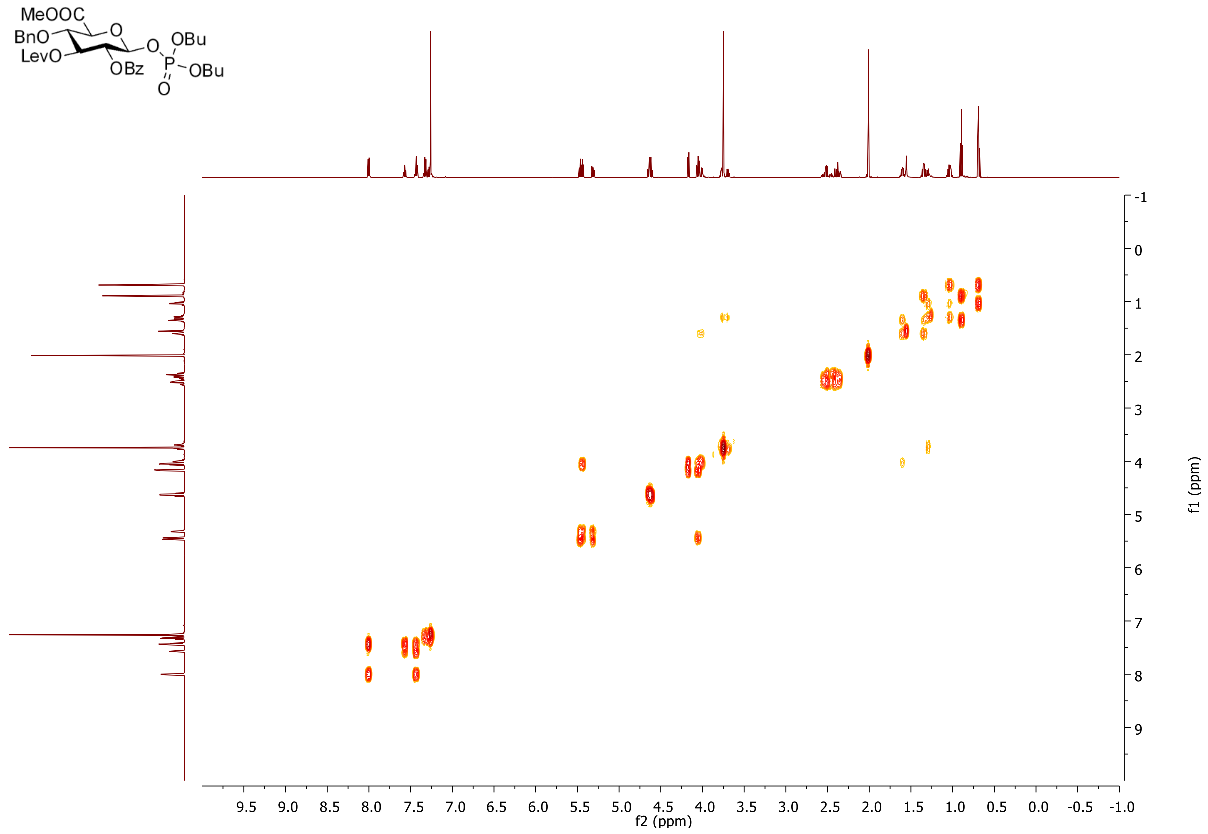

Compound **1**: HSQC NMR

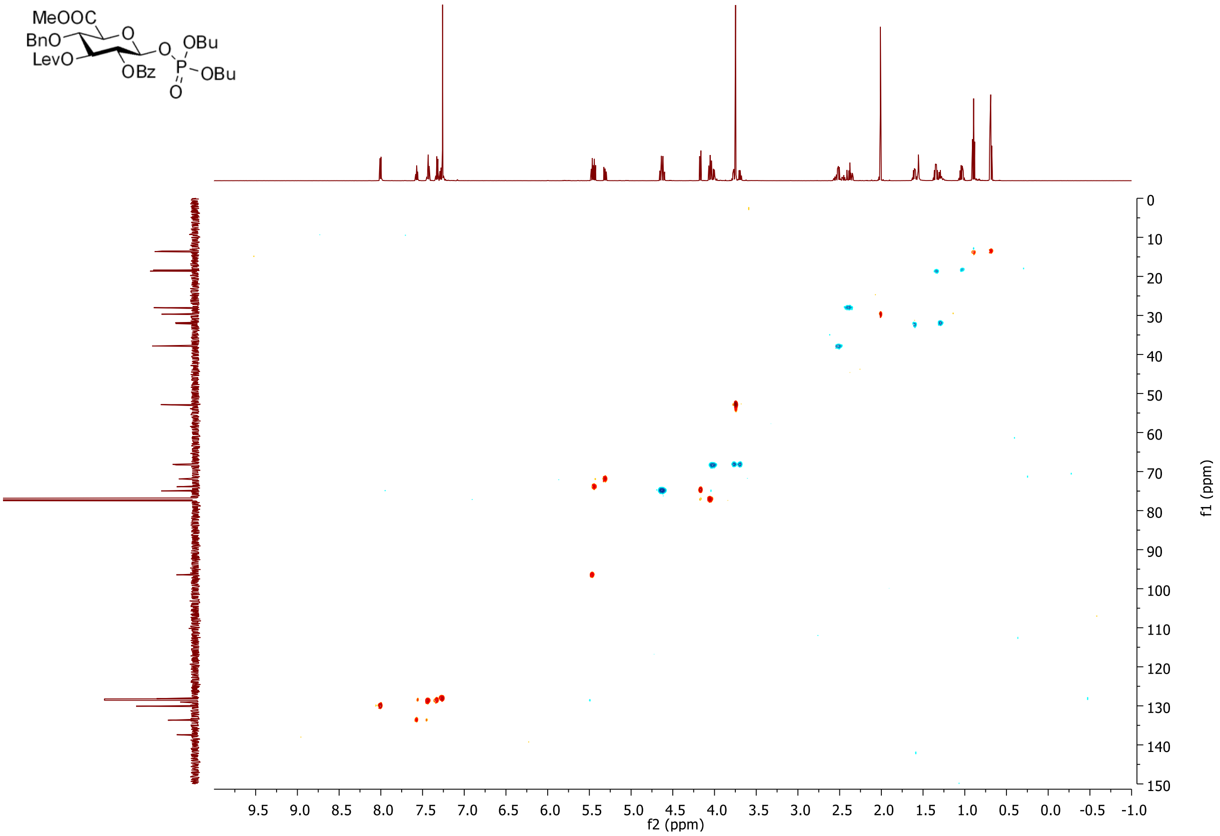

Compound **1**: ^{13}C NMR

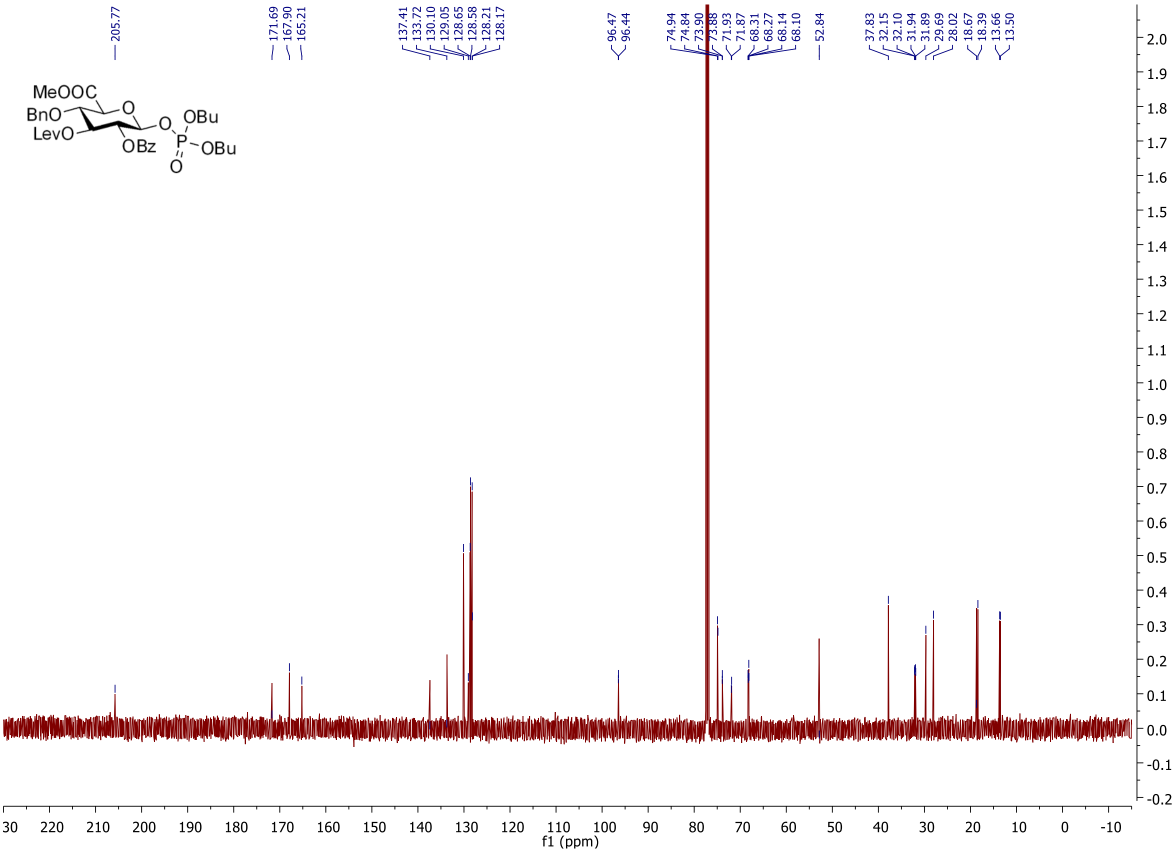

Compound **1**: ^{31}P NMR

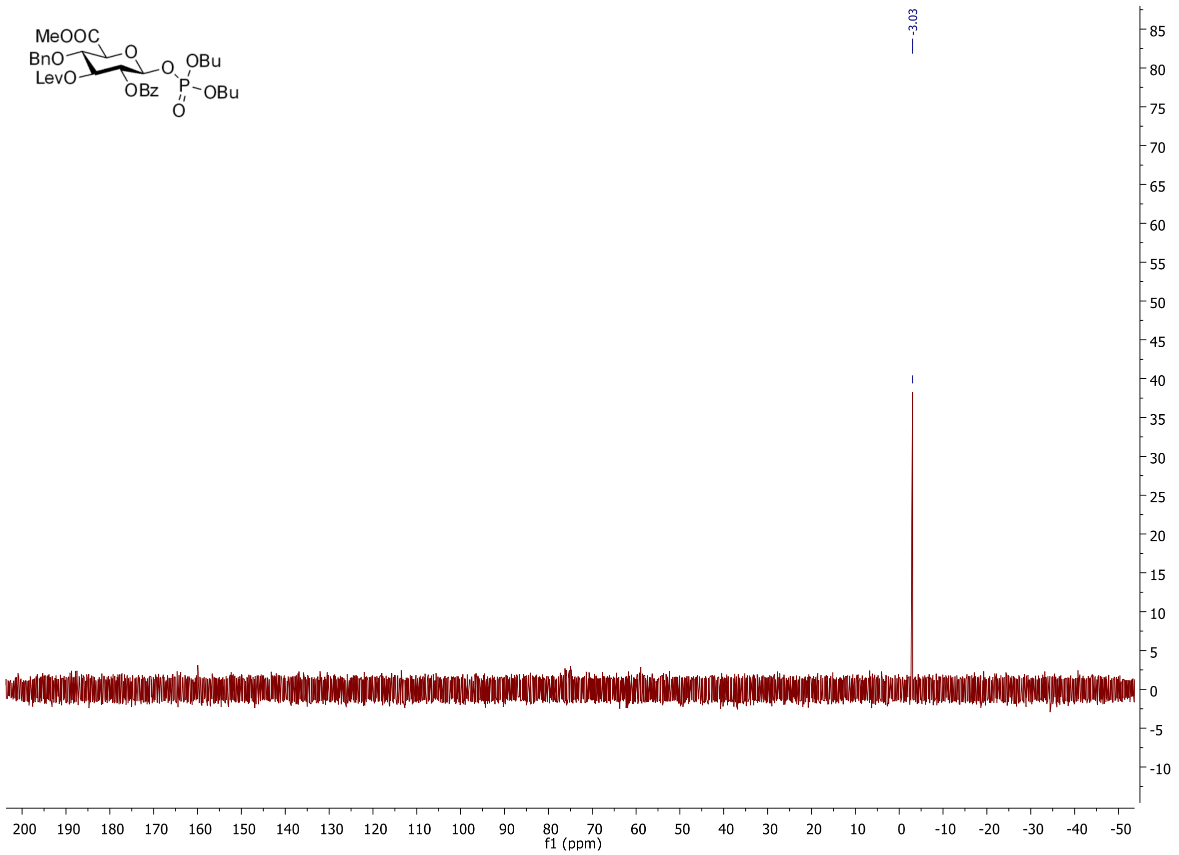

Compound **2**: ^{1}H NMR

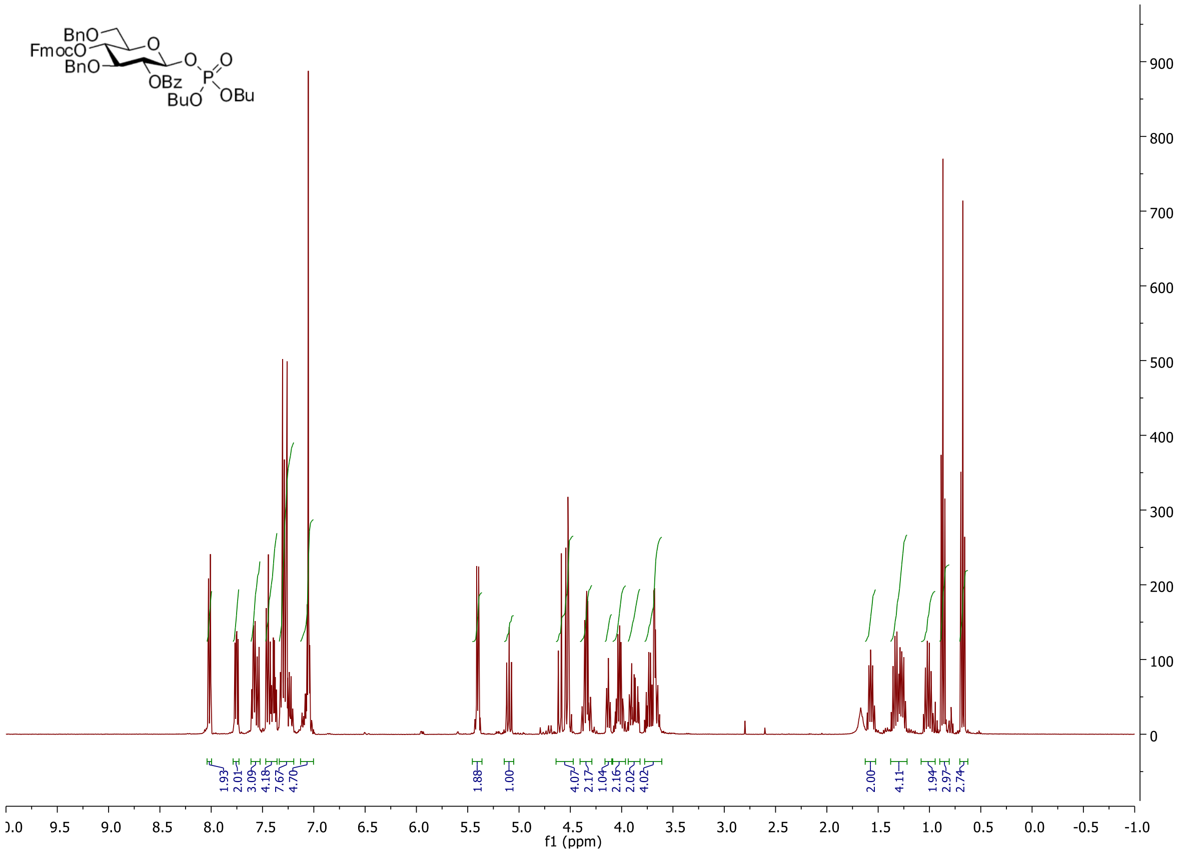

Compound **2**: HH-COSY NMR

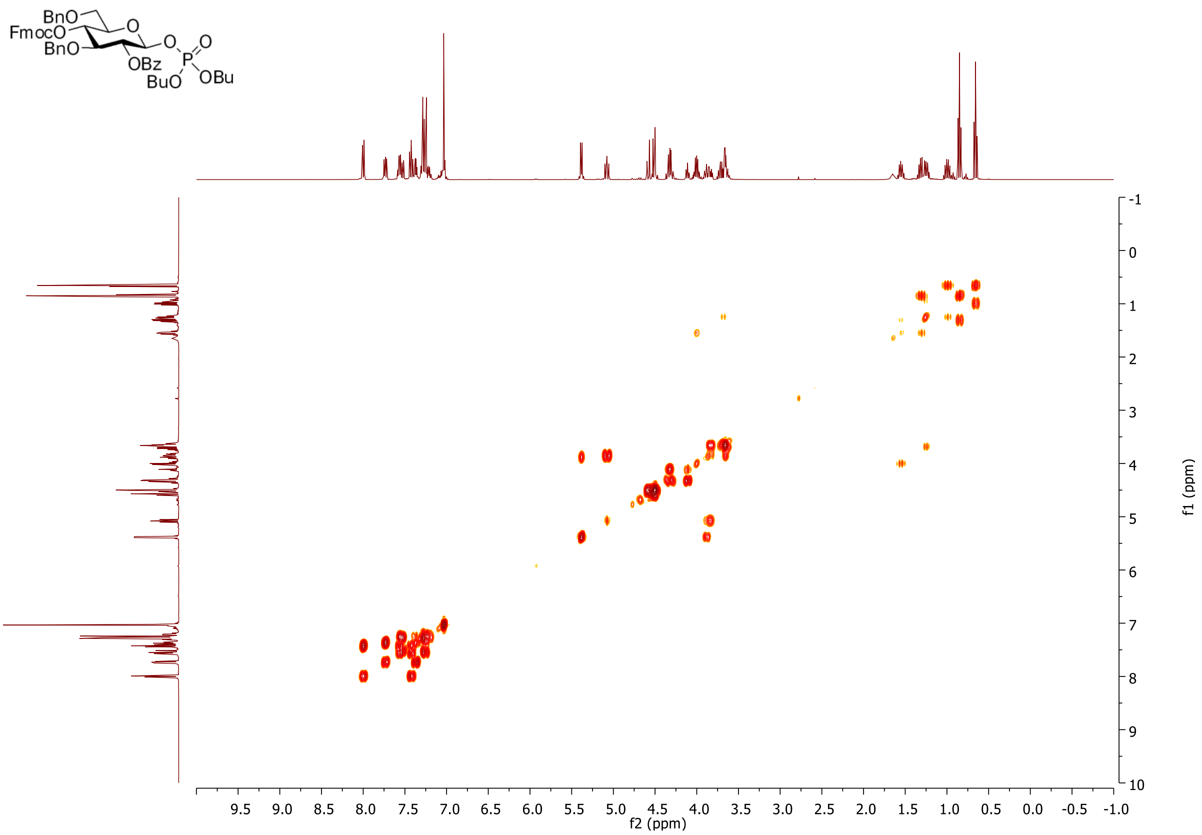

Compound **2**: HSQC NMR

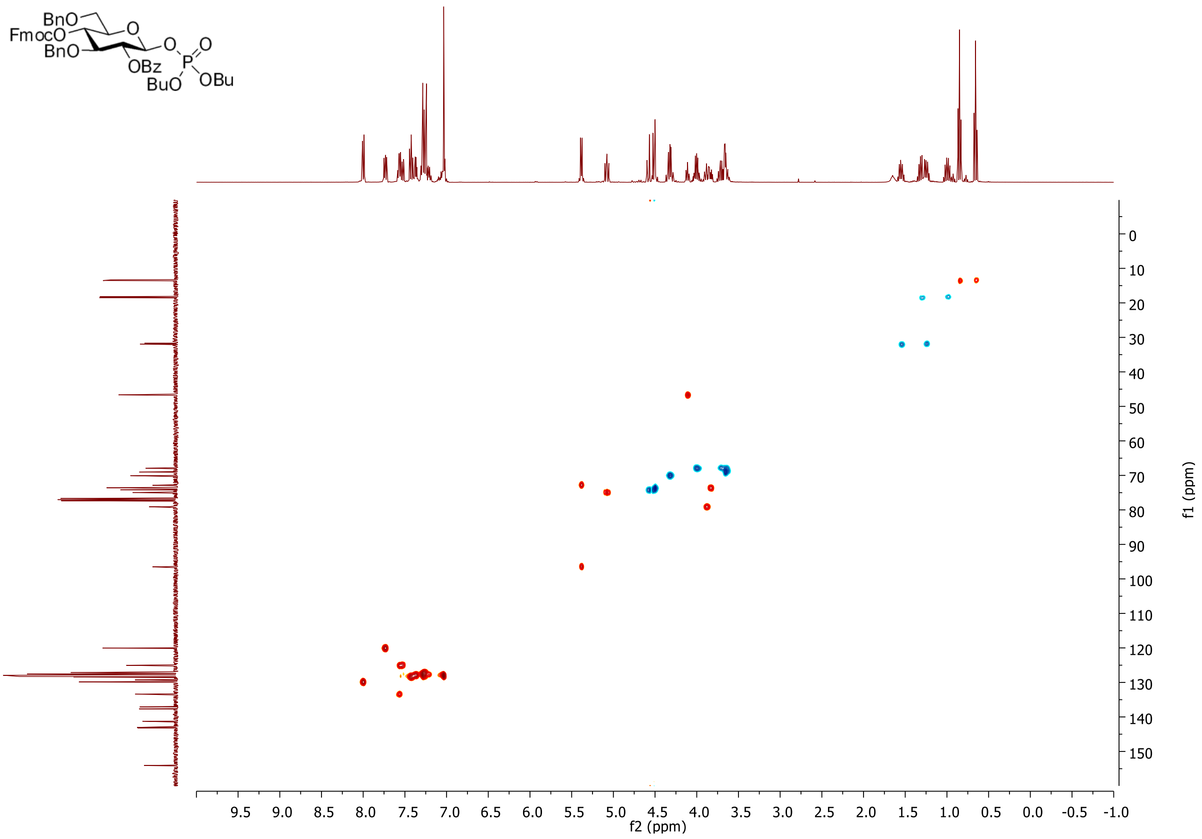

Compound **2**: ^{13}C NMR

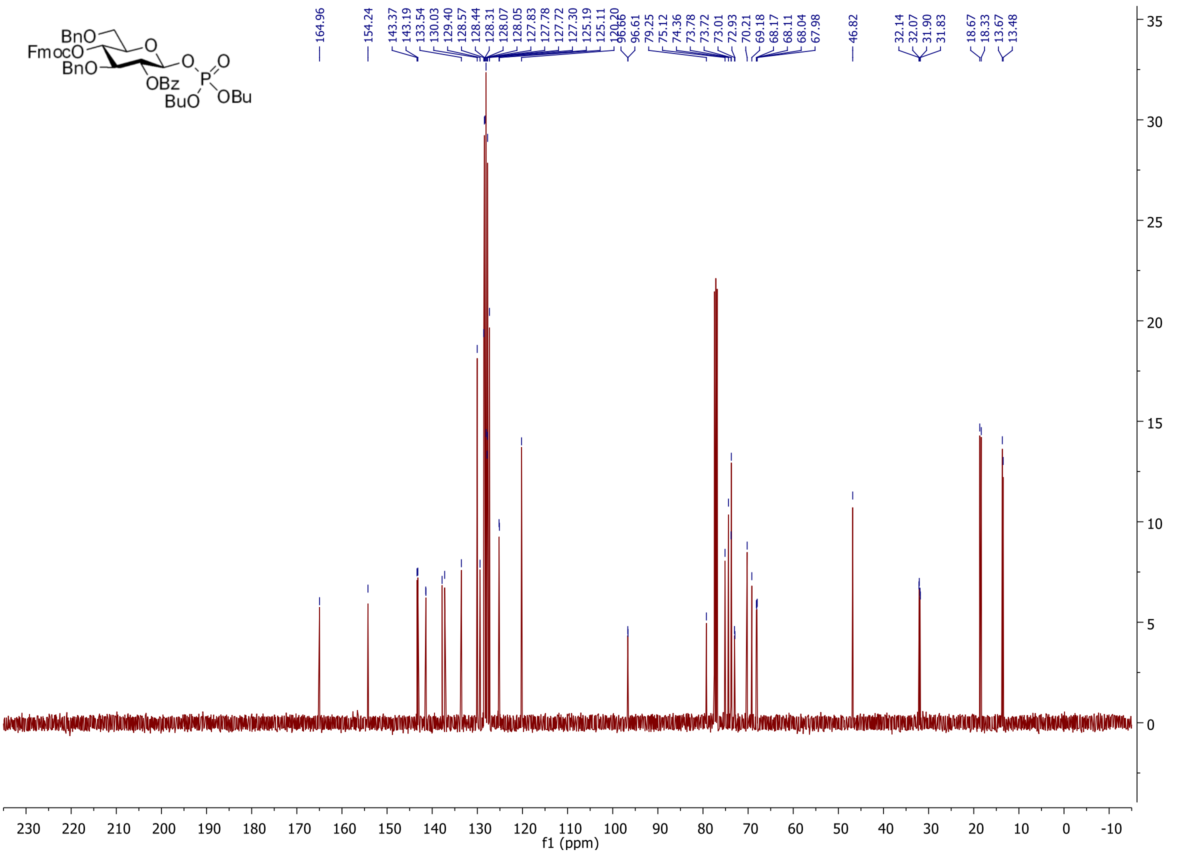

Compound **2**: ^{31}P NMR

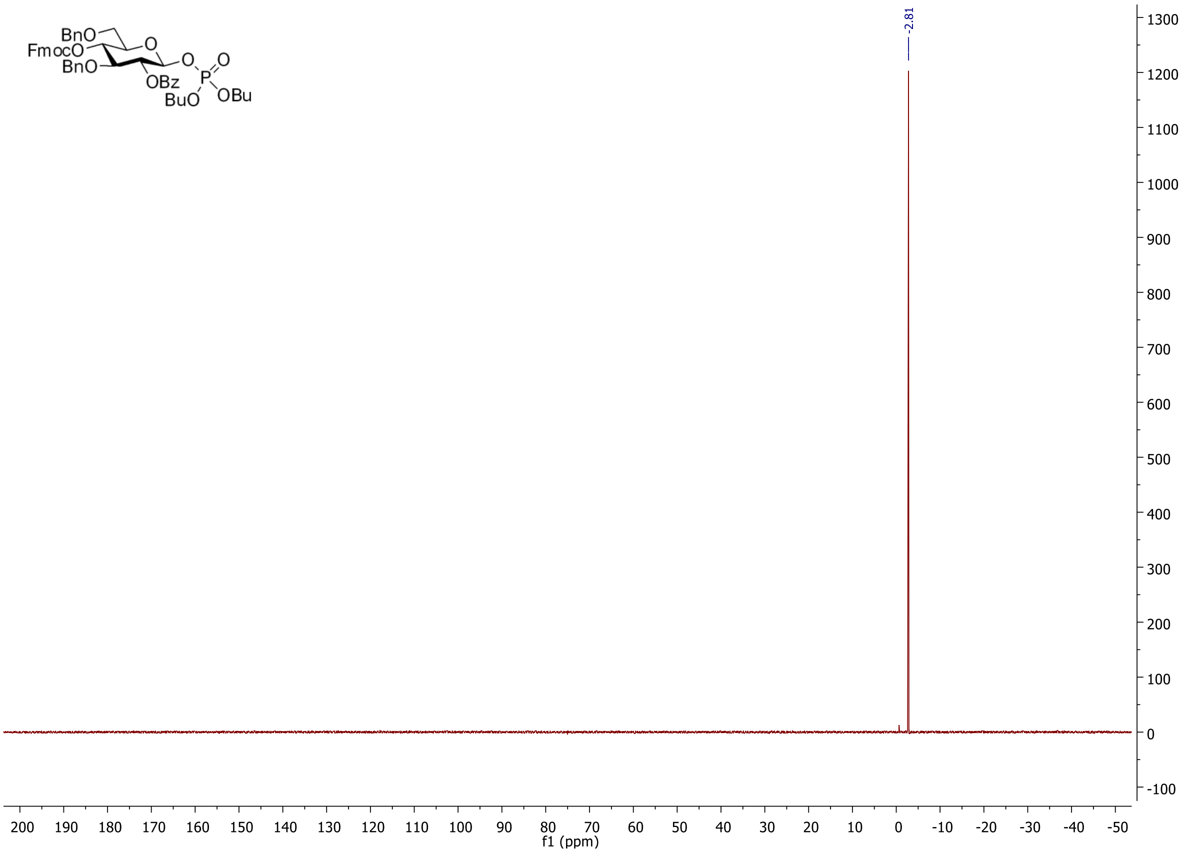

Compound **3**: ^{1}H NMR

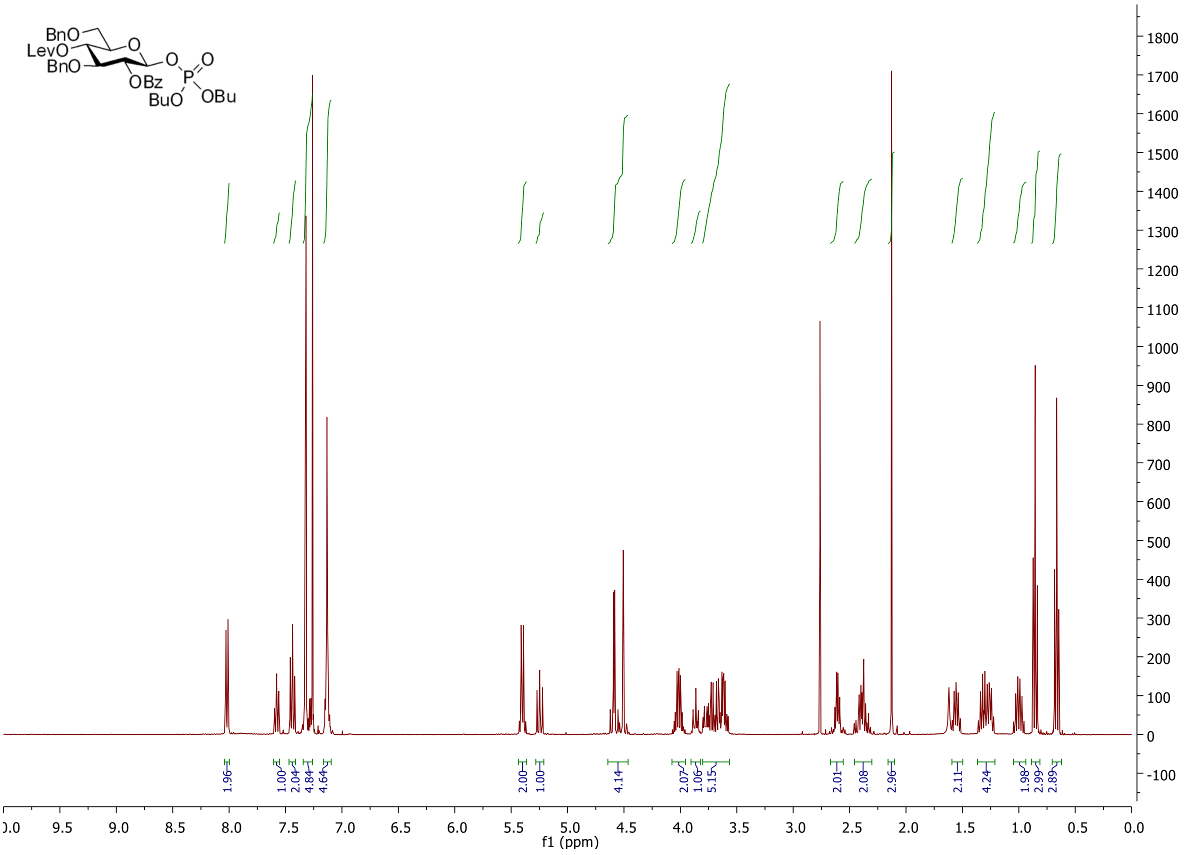

Compound **3**: HH-COSY NMR

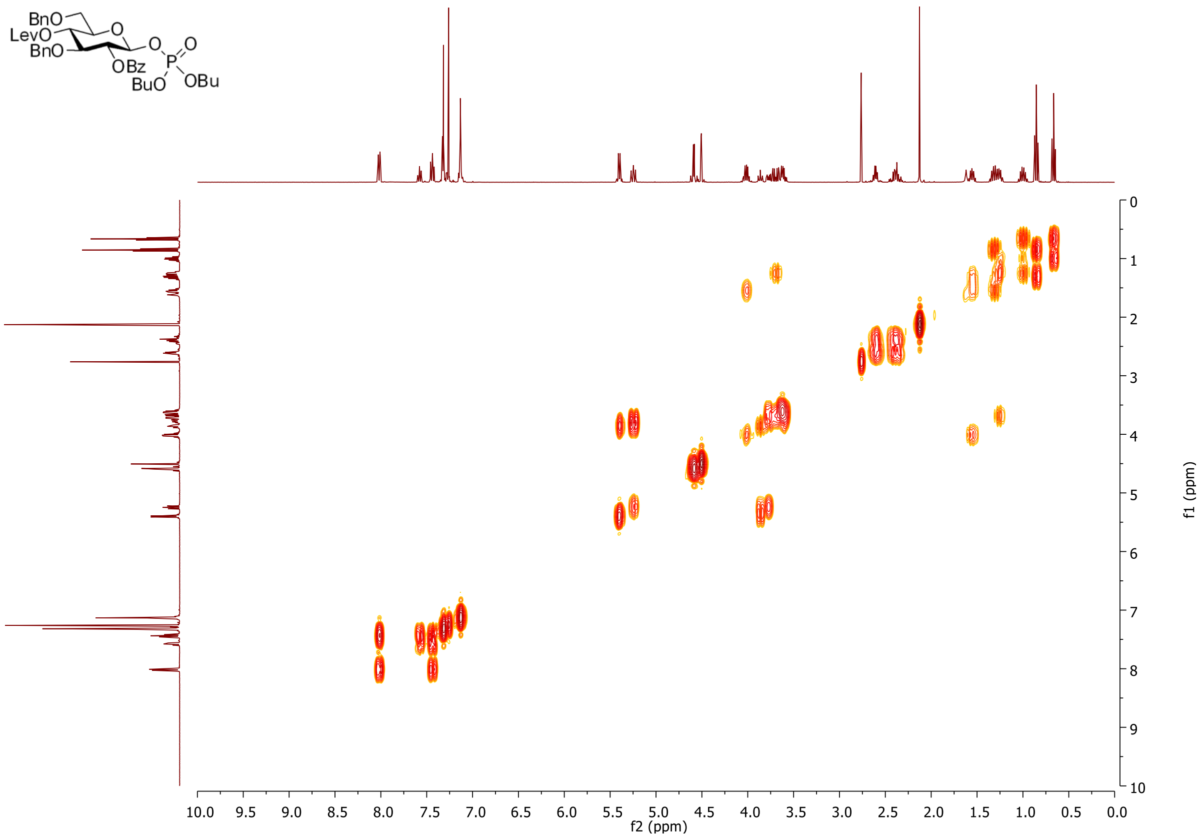

Compound **3**: HSQC NMR

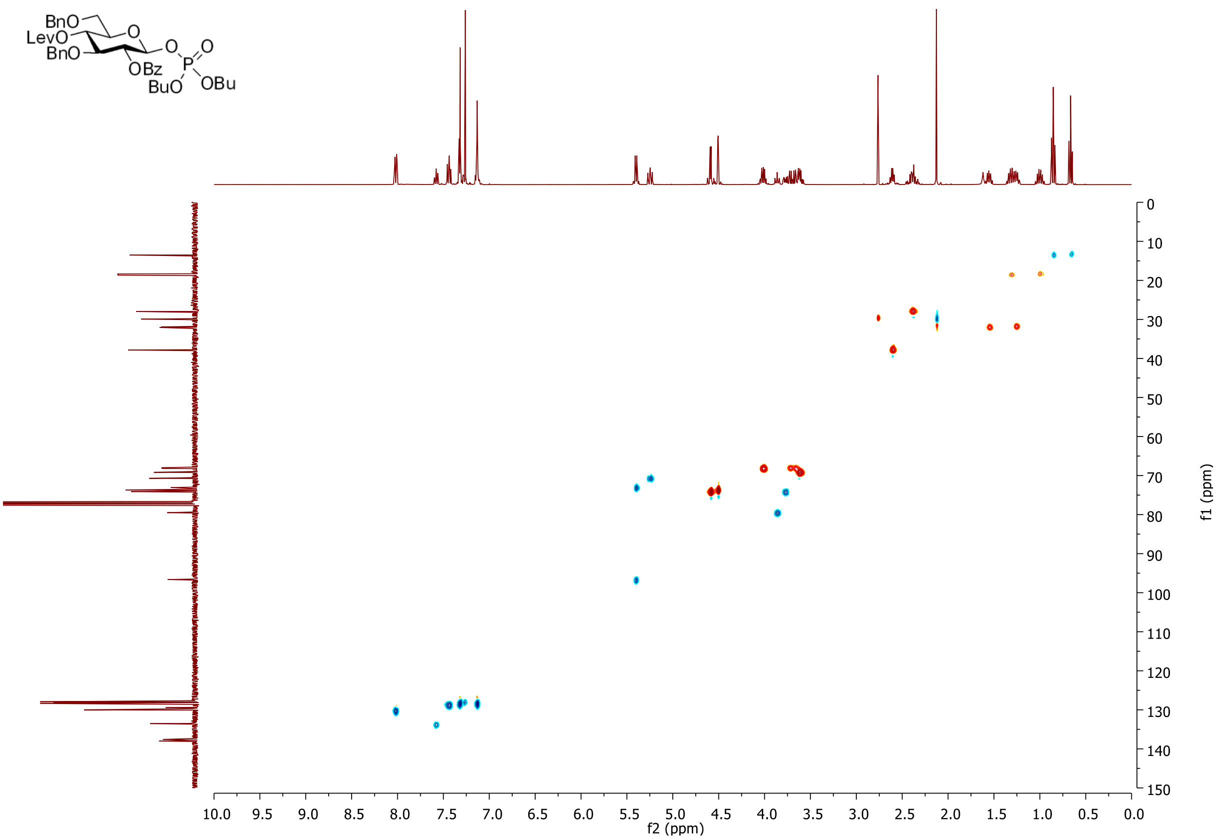

Compound **3**: ^{13}C NMR

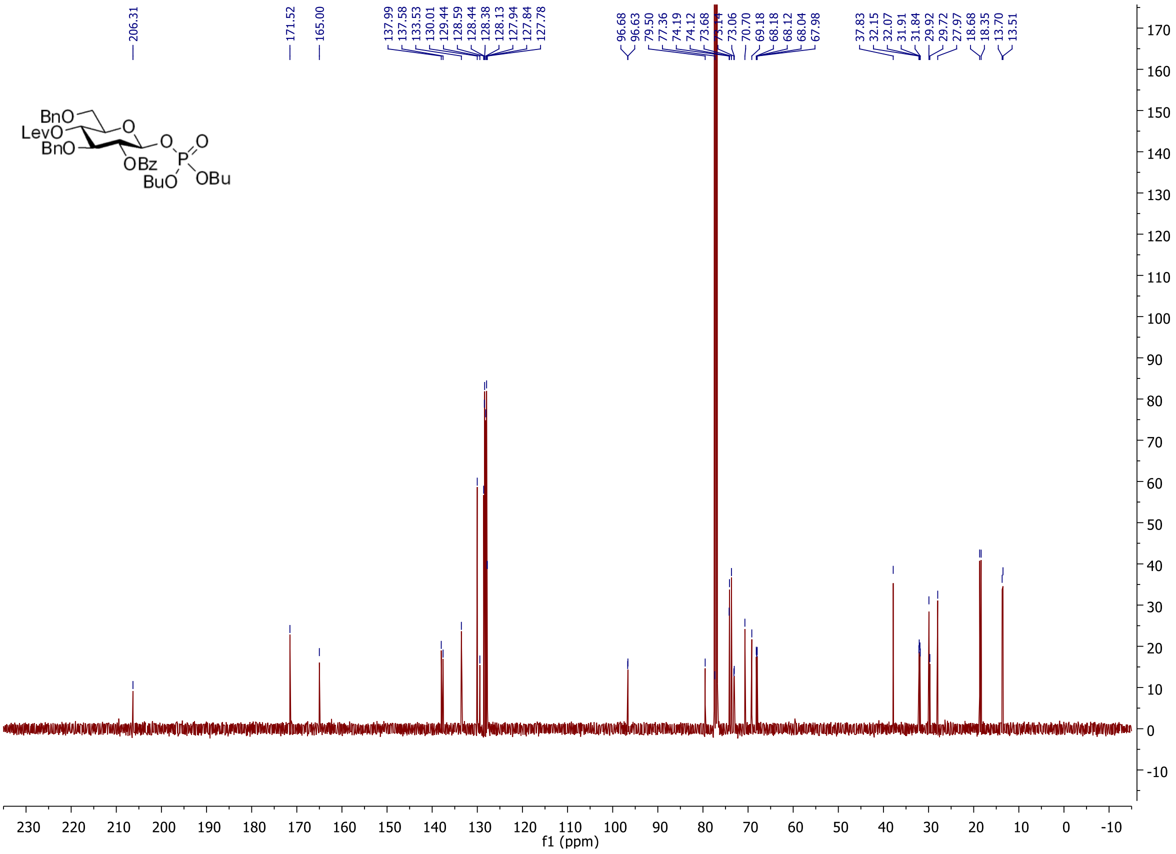

Compound **3**: ^{31}P NMR

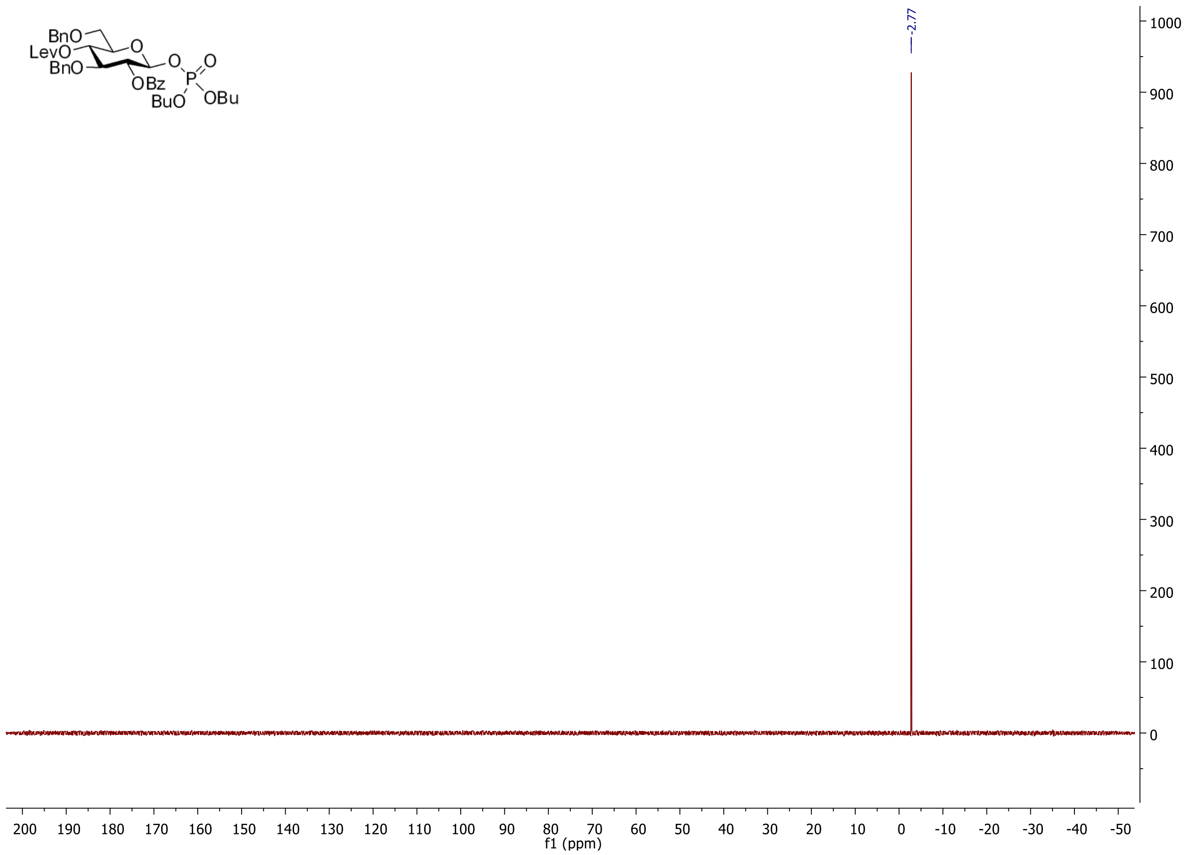

Compound **5**: ^{1}H NMR

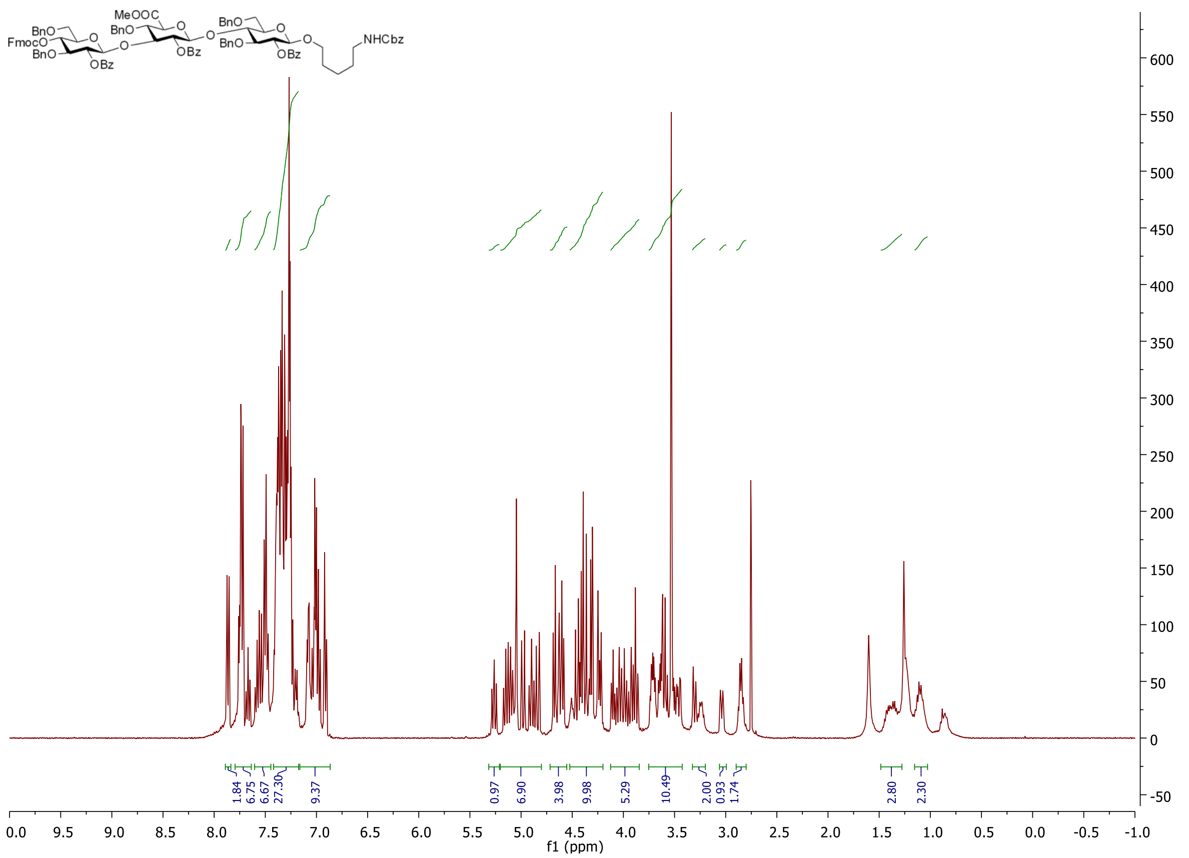

Compound **5**: HH-COSY NMR

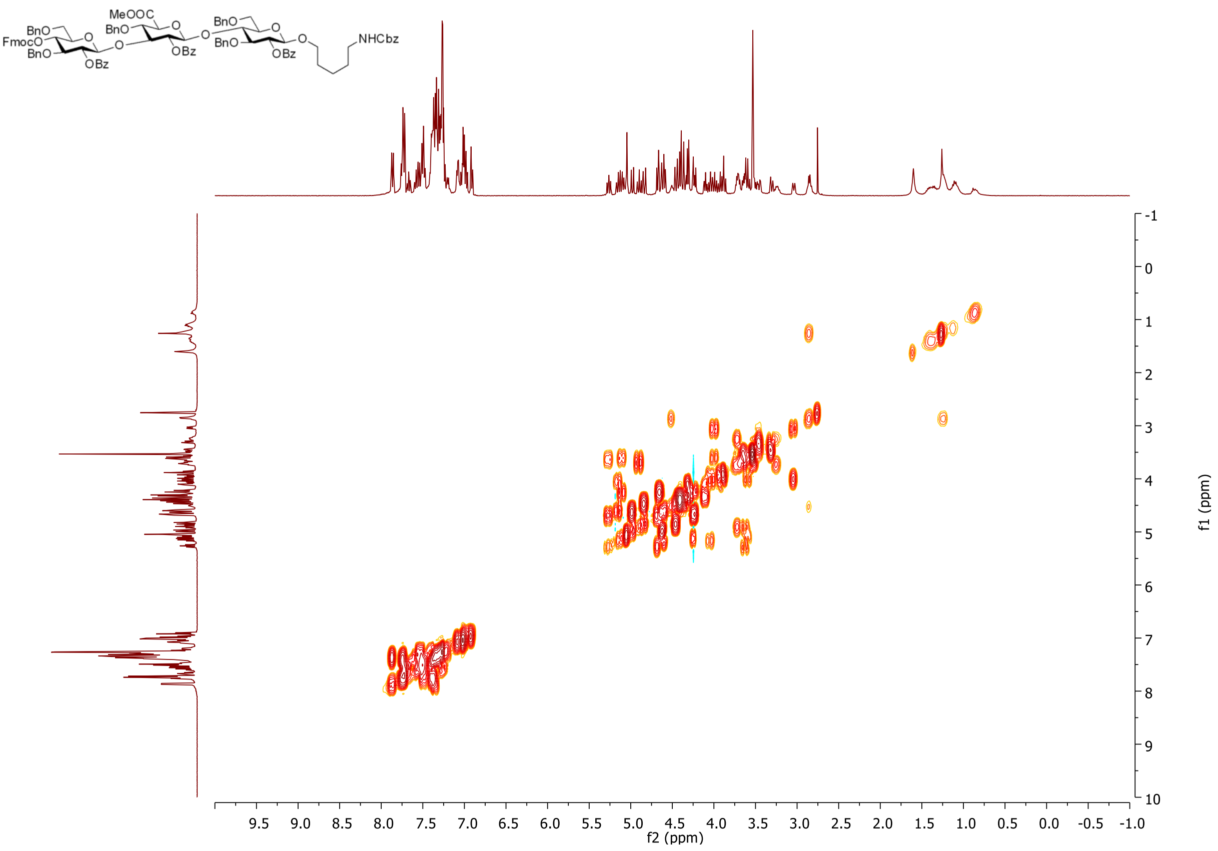

Compound **5**: HSQC NMR

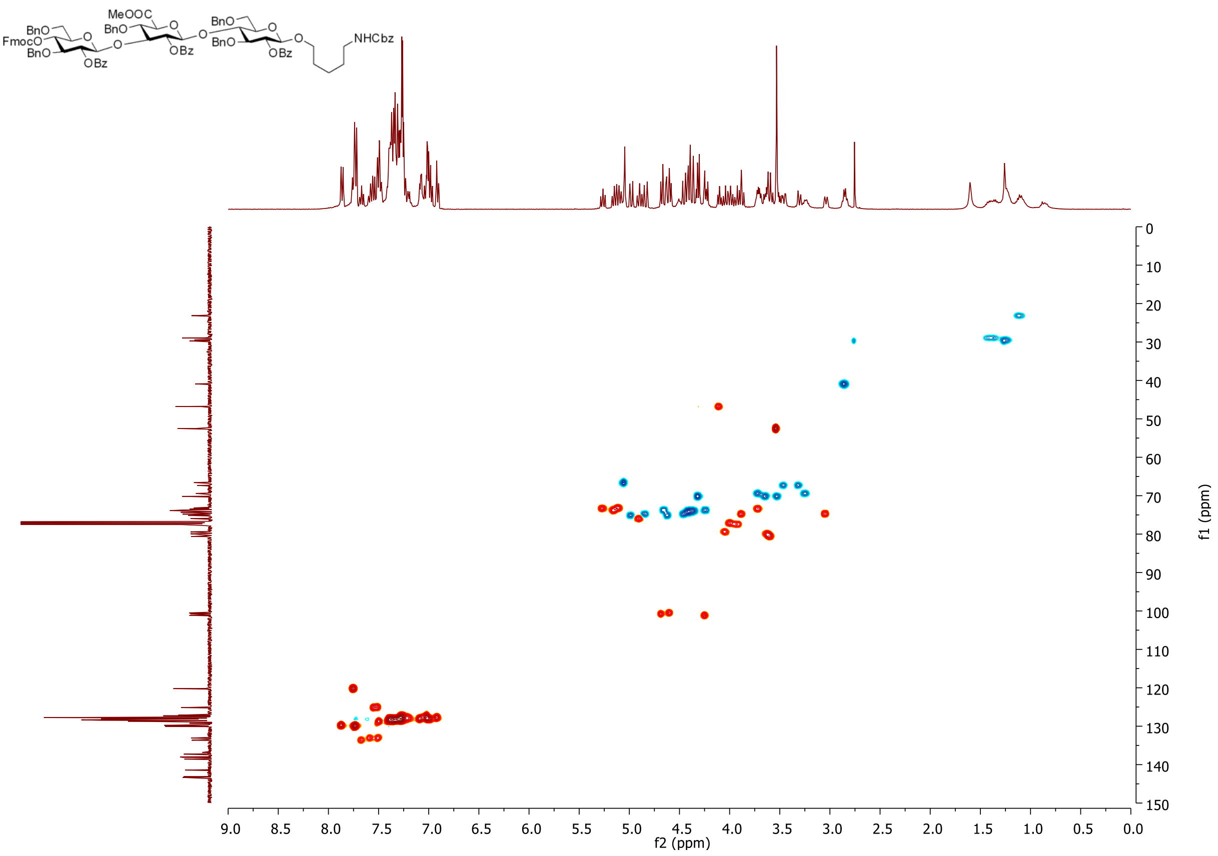

Compound **5**: ^{13}C NMR

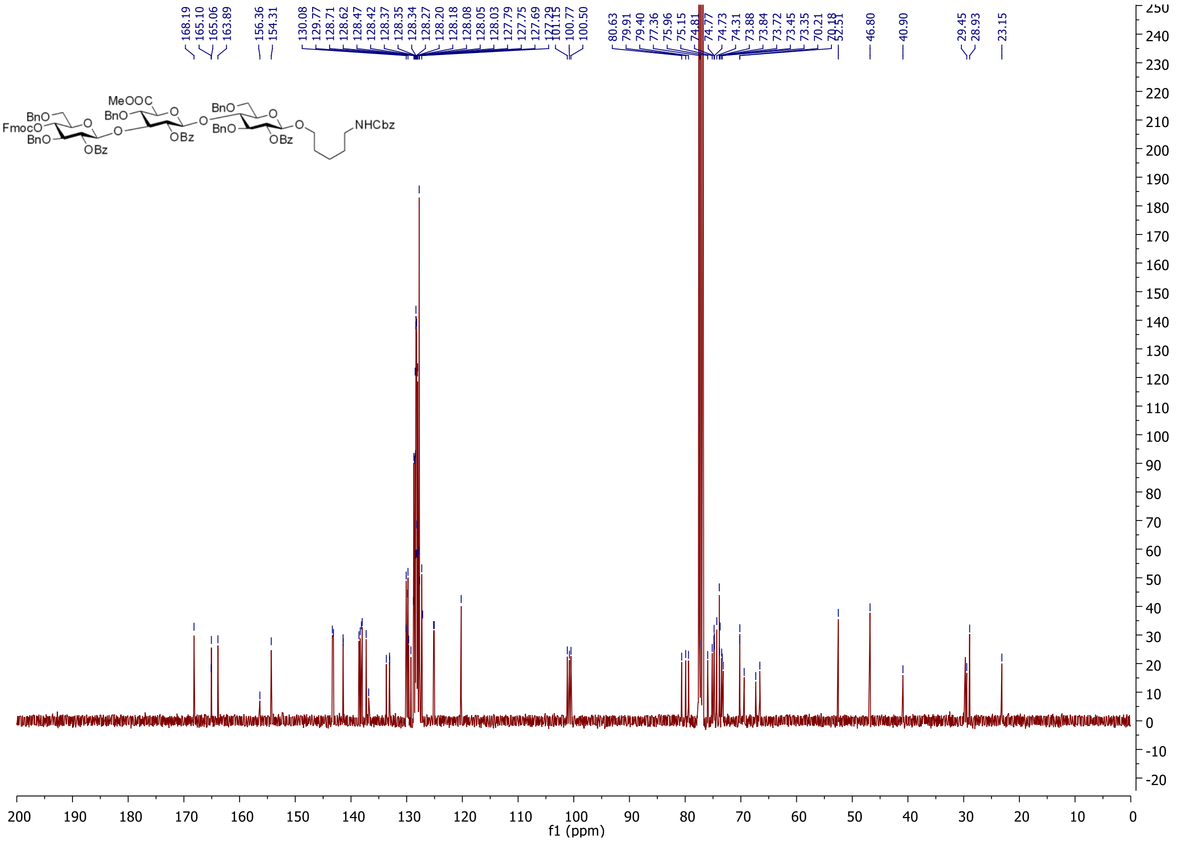

Compound **11**: ^{1}H NMR

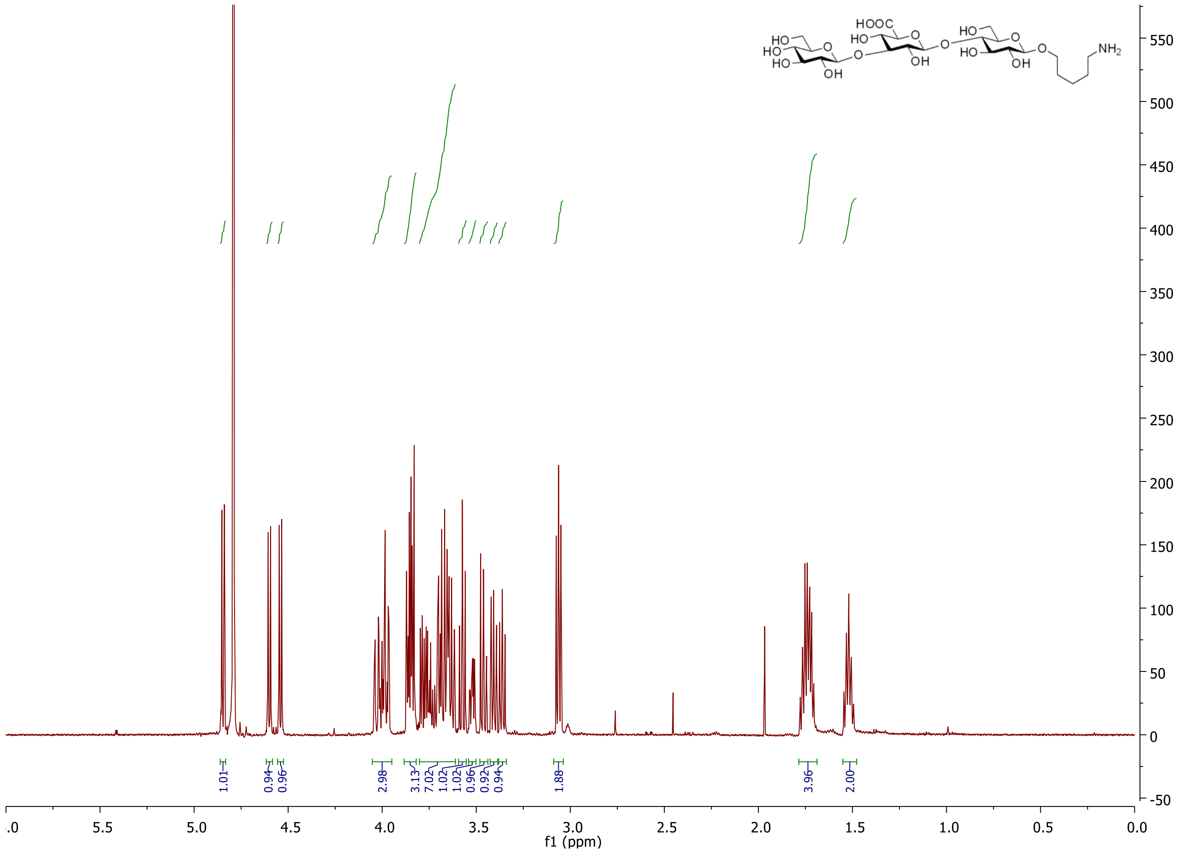

Compound **11**: HH-COSY NMR

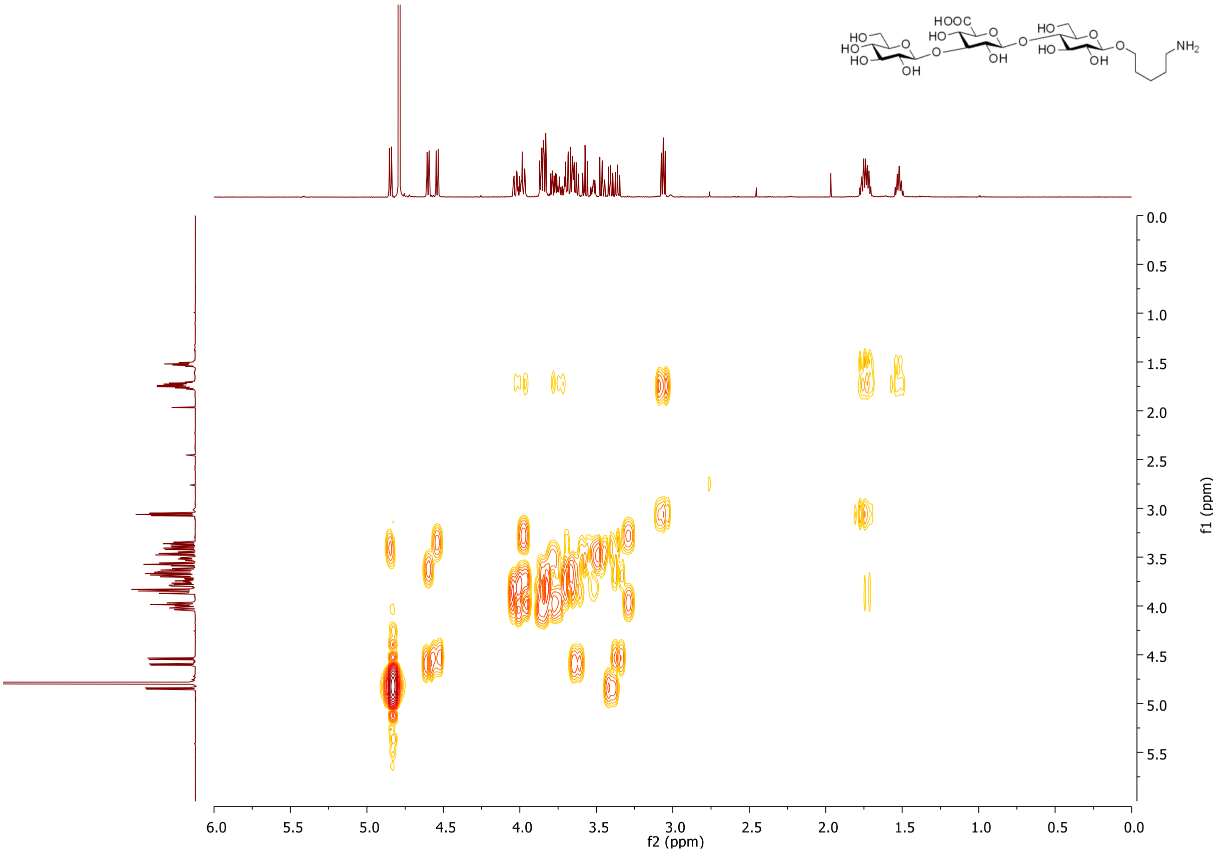

Compound **11**: HSQC NMR

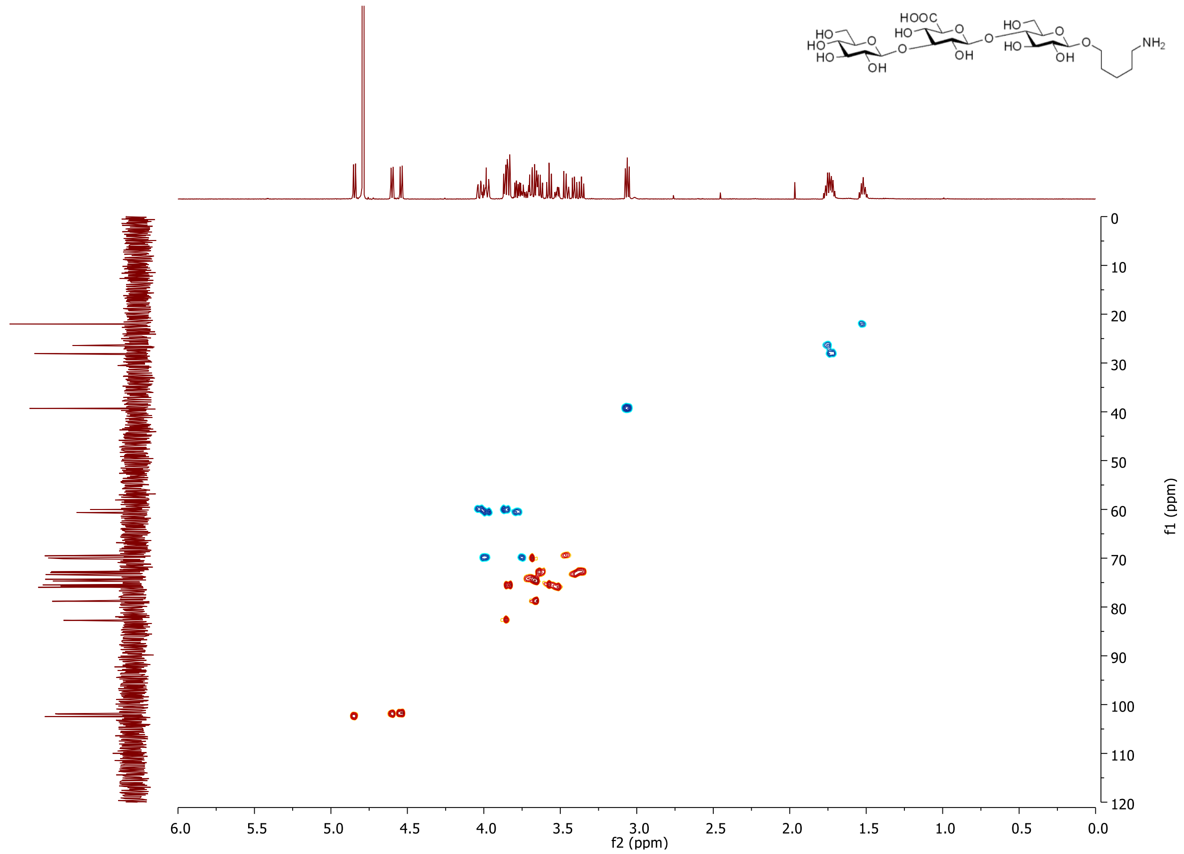

Compound **11**: Coupled HSQC NMR

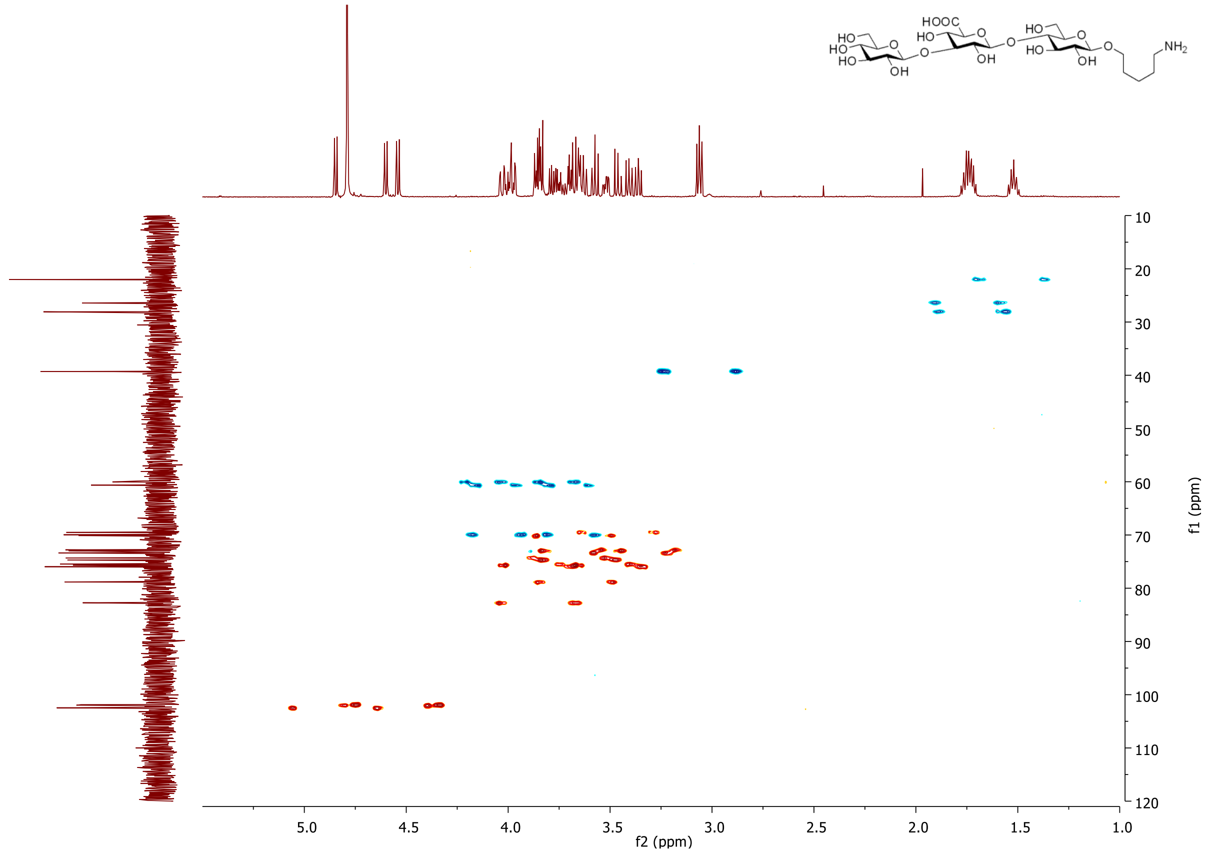

Compound **11**: ^{13}C NMR

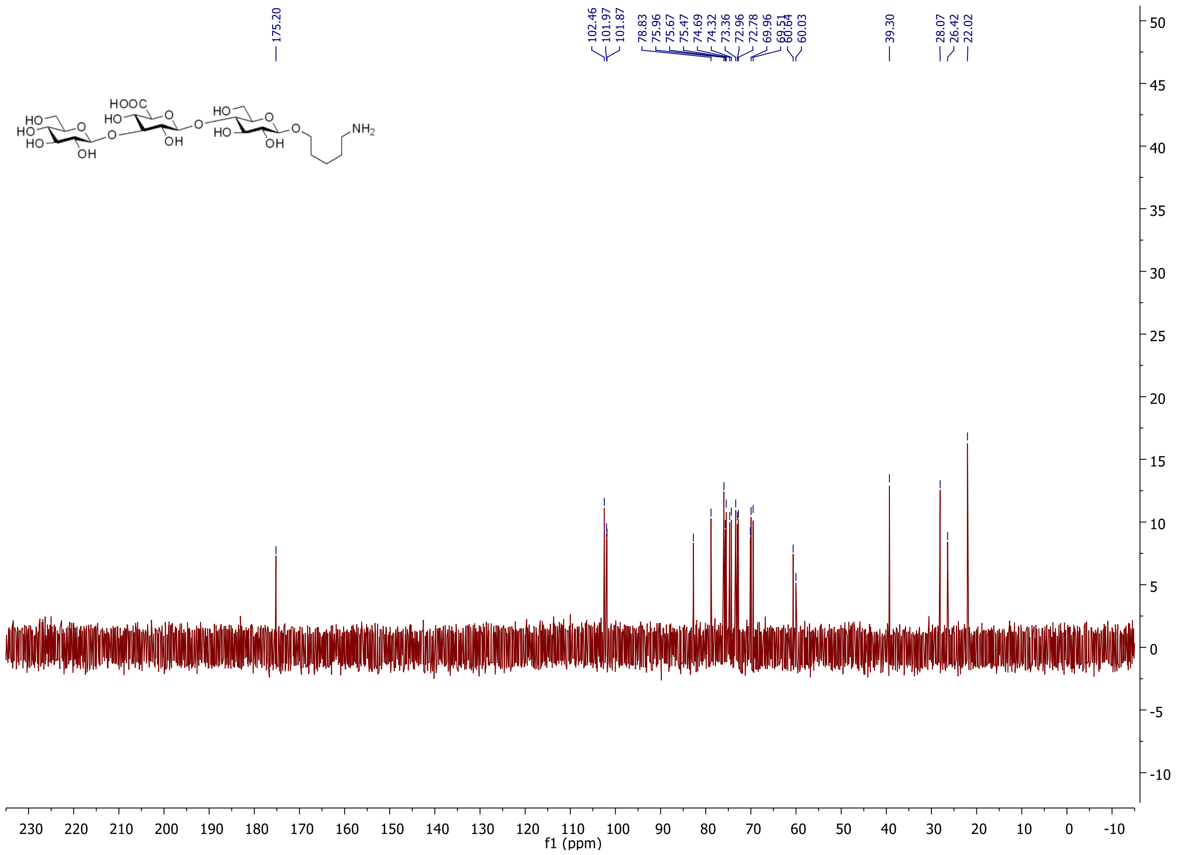

Supplement: File 1 — Experimental details as well as full characterization of all new compounds. [file Beilstein_J_Org_Chem-12-1440-s001.pdf]
